# Exploring optimal drug targets through subtractive proteomics analysis and pangenomic insights for tailored drug design in tuberculosis

**DOI:** 10.1038/s41598-024-61752-6

**Published:** 2024-05-13

**Authors:** Muhammad Fayaz Khan, Amjad Ali, Hafiz Muzzammel Rehman, Sadiq Noor Khan, Hafiz Muhammad Hammad, Maaz Waseem, Yurong Wu, Taane G. Clark, Abdul Jabbar

**Affiliations:** 1 Department of Medical Laboratory Technology, The University of Haripur, Haripur, KP Pakistan; 2 Atta-ur-Rahman School of Applied Biosciences, National University of Sciences and Technology, Islamabad, Pakistan; 3 School of Biochemistry and Biotechnology, University of the Punjab, Lahore, Punjab Pakistan; 4 Department of Chemistry, The Hong Kong University of Science and Technology, Kowloon, Hong Kong China; 5 London School of Hygiene and Tropical Medicine, Keppel Street, London, UK

**Keywords:** Drug target identification, Molecular docking and MD simulation, Drug discovery, Computational biology and bioinformatics, Drug discovery

## Abstract

Tuberculosis (TB), caused by *Mycobacterium tuberculosis*, ranks among the top causes of global human mortality, as reported by the World Health Organization’s 2022 TB report. The prevalence of *M. tuberculosis* strains that are multiple and extensive-drug resistant represents a significant barrier to TB eradication. Fortunately, having many completely sequenced *M. tuberculosis* genomes available has made it possible to investigate the species pangenome, conduct a pan-phylogenetic investigation, and find potential new drug targets. The 442 complete genome dataset was used to estimate the pangenome of *M. tuberculosis*. This study involved phylogenomic classification and in-depth analyses. Sequential filters were applied to the conserved core genome containing 2754 proteins. These filters assessed non-human homology, virulence, essentiality, physiochemical properties, and pathway analysis. Through these intensive filtering approaches, promising broad-spectrum therapeutic targets were identified. These targets were docked with FDA-approved compounds readily available on the ZINC database. Selected highly ranked ligands with inhibitory potential include dihydroergotamine and abiraterone acetate. The effectiveness of the ligands has been supported by molecular dynamics simulation of the ligand–protein complexes, instilling optimism that the identified lead compounds may serve as a robust basis for the development of safe and efficient drugs for TB treatment, subject to further lead optimization and subsequent experimental validation.

## Introduction

Tuberculosis (TB) proved a challenging global challenge, claiming 1.6 million lives, including 187,000 individuals grappling with TB and HIV. TB was ranked the world’s second-most severe infectious disease after COVID-19 and ultimately the thirteenth most prevalent cause of mortality. Significantly, throughout the year, there were 1.4 million deaths from TB among individuals without HIV and 187,000 deaths among those with HIV, adding to the total fatalities. This figure represents a rise compared to previous years, in line with the levels observed in 2017, underscoring the ongoing urgency of addressing TB within the broader context of global health^1^.

TB primarily spreads through airborne transmission, with *M. tuberculosis* infiltrating the human body and subverting host immune defenses. Within the host, the bacteria adeptly invade macrophages, establishing a prolonged presence that leads to chronic infection, a manifestation of compromised host immunity^2^. *M. tuberculosis* strains are increasingly becoming multi and extensively drug resistant (MDR & XDR) as the genes of the bacteria naturally evolve, making them more challenging against the medicines typically employed for tuberculosis treatment. Consequently, these mutated strains emerge as dominant players in the population, outcompeting their more susceptible counterparts. This phenomenon underscores the critical role of genetic adaptation in the evolution of drug resistance, presenting a substantial challenge in managing and controlling TB. Consequently, the efficacy of conventional anti-TB medications such as isoniazid and rifampicin has been compromised^3^.

Advancements in genomics and other fields have made it possible to more fully comprehend the functional importance of many distinct proteins encoded within the *M. tuberculosis* genome since the first genome sequencing of *M. tuberculosis* in 1998^4,5^. The pangenome concept can now be thoroughly investigated, especially to identify the core genome, denoting the set of genes consistently identified across all strains within the dataset^6^. Utilizing these genes presents an opportunity to develop comprehensive therapeutic interventions targeting pathogenic species across a broad spectrum^2^.

Bioinformatics tools are essential in systematically analyzing pathogen data for potential drug targets in drug development. Similarly, advances in cheminformatics, like those shown by resources like DrugBank, allow for efficient virtual screening of ligand-target binding affinities. Computer-aided drug discovery (CADD) accelerates precision in identifying therapeutic candidates within the complex landscape of pathogenic biology^7,8^. Scientists have made significant efforts to find promising compounds for TB by utilizing these invaluable resources^8,9^. Hence, for the efficient prioritization of potential drug targets in the *M. tuberculosis* genome, an integrative genomics approach was adopted in this study that included pangenome analyses, subtractive proteomics, and CADD. Moreover, using molecular docking, the structural interactions between these potential targets and FDA-approved drugs were investigated. Employing this method led to the identification of a group of promising lead molecules, marking notable progress in the effort to develop drugs against TB.

The traditional drug development method requires at least ten years of in-depth study and significant funds. However, integrating computer-aided analyses during the initial phases can significantly reduce this endeavor’s time and financial investments^10,11^. In drug discovery, in silico drug screening is a valuable technique, allowing for the focused selection of compounds with the most significant relevance for subsequent experimental investigations. This approach streamlines the research process by eliminating biomolecules that do not meet the necessary criteria, contributing to the refinement and enhancement of drug discovery efforts as a whole^12^. All the identified high-ranking potential targets were analyzed rather than focusing exclusively on a single drug target. This study can potentially establish a solid basis for developing innovative therapies for the challenging bacterial pathogen *M. tuberculosis*.

## Materials and methods

### Collection of genomic data

The genome sequences of all strains of *M. tuberculosis* currently in existence, along with their corresponding proteomes, were obtained from the PATRIC database (accessed on May 26, 2023; accessible at https://www.bv-brc.org/)^13^. These strains were collected from different parts of the world. Pan-genome analysis was made possible by the subsequent integration of these genomic data.

### Pangenome analysis of *M. tuberculosis* strains

The bacterial pan-genome analysis (BPGA) tool was used to perform pangenome analysis on retrieved genomes to find highly conserved proteins from *M. tuberculosis*. The default 50% identity threshold and the USEARCH algorithm were used to produce orthologous protein clusters. BPGA computed the pan and core proteome sizes by taking into account 20 permutations and gradually calculating median values as each genome was added. A ratio of the total number of unique and common gene families to the total number of genomes was used to establish the core and pan-genome curves graphically. The results also gave insights into how each genome’s inclusion led to the emergence of new genes^14^. To identify potential therapeutic targets, the analysis was further focused on protein sequences associated with the core genome.

### Identification of non-host homologous, essential, and virulence-associated proteins

PanRV was used to identify new therapeutic targets within the core proteome. This strategy includes several screening stages, such as filters for non-homology, virulence, and essentiality^15^. Ensuring the safety of drug therapy in humans and minimizing potential adverse effects necessitates identifying drug targets distinct from human proteins. The core genome was compared to the human genome using the NCBI BLASTp tool (with an e-value 1e−5)^16^. Essential genes were determined from the subset of non-host homologous conserved proteins by conducting BLASTp searches against the database of essential genes (DEG) with a focus on stringent criteria (e-value < 0.0001 and a bit score > 100)^17^. DEG compiles data from eukaryotes, bacteria, and archaea that have been experimentally verified. This information contains essential genomic components required for a cell’s survival. The selected proteins were then subjected to BLASTp searches within the virulence factor database (VFDB) to find potential proteins linked to virulence^18^. The VFDB is a substantial online database that details bacterial pathogens’ virulence characteristics. Virulence factors are gene products that enhance a bacterium’s ability to cause disease^19^.

### Identification of putative *M. tuberculosis* drug targets

Physiochemical properties were used to refine filtered proteins further and find potential drug targets. The molecular weight, isoelectric point, GRAVY (grand average of hydropathicity) value, instability index, and aliphatic index were considered using the ProtParam tool (found at https://web.expasy.org/protparam/)^20^. Proteins with low molecular weight are particularly favorable as drug targets, given their susceptibility to drug interactions^21^. A higher aliphatic index value indicates enhanced thermostability, whereas a lower GRAVY value signifies that proteins are hydrophilic^22^. After being physiochemically characterized, a comparative pathway analysis was used to analyze the filtered proteins further. The KEGG Automatic Annotation Server (KAAS) version 2.1 was used to identify proteins linked to pathogen-specific pathways. This strategic method improves the possibility of precisely targeting the pathogen^23^.

Finally, the selected proteins druggability was evaluated using BLASTp with default parameters against the Drug Bank database (https://go.drugbank.com/)^24^. The draggability of possible targets is another vital screening stage that assesses the likelihood that a drug will regulate selected targets^25^. Moreover, all the FDA-approved ligands from the ZINC database (accessed at https://zinc.docking.org/ on 23, August 2023) were retrieved in SDF format and checked against identified proteins which were found druggable earlier in druggability analysis^26^.

### Molecular docking of putative drug targets with drugs

The sequences of the identified drug targets were examined for the presence of corresponding crystal structures in the RCSB Protein Data Bank (accessible at https://www.rcsb.org/)^27^. The relevant PDB files were obtained from the RCSB protein databank, and any attached ligands were taken out of the crystal structures if they were there. The structural configurations of the drug targets were molecularly docked with the drugs previously identified in the draggability analysis using PyRx’s AutoDock Vina tool^28,29^. The grid box was modified during the molecular docking process to enclose the protein space entirely, and blind docking was conducted, selecting a value of 32 for exhaustiveness. The targets and the corresponding ligands were prepared in the PDBQT format for molecular docking analysis. The visualization of docked complexes and the interactions formed was performed in Discovery Studio Visualizer. To evaluate the effectiveness of the drug, both the binding affinity $E$ (in kcal/mol) from AutoDock Vina and the fit quality score are used^30^, where the fit score is defined as follows:

$$LE=\frac{E}{HA}$$

$$FQ=\frac{LE}{LE\_Scale}$$

$$LE\_Scale=-0.064+0.873{e}^{-0.026HA}$$

In these equations, $HA$ is the number of heavy (non-hydrogen) atoms, $LE$ is the ligand efficiency, $LE\_Scale$ is the scaling factor, and $FQ$ is the fit quality score. An $FQ$ of near or larger than 1 indicates that the ligand binding is optimal, while a small $FQ$ represents a sub-optimal binding. A comparative study of the docked complexes with the lowest binding energies was performed to identify ligands strongly linked to the drug targets. As a result, these preferred ligands were chosen for later molecular dynamic simulations.

### Molecular dynamics simulation

Molecular dynamics (MD) simulation is a powerful computational technique used to study the behavior and properties of molecules and materials at the atomic level. Desmond software package was used for MD simulations of biological systems, including proteins, nucleic acids, and membranes.

The Desmond module of Schrodinger was exploited to conduct the MD simulation studies. The dynamic behavior and stability of the protein–ligand complex were investigated using its docked poses. The protein–ligand complex was preprocessed using the Protein Preparation Wizard of Maestro, which included complex optimization and minimization. All of the systems were prepared using the System Builder tool. The solvation of the complexes was performed with the simple point-charge (SPC) water model with an orthorhombic box, along with a 10-Å distance from the edge of the box, and the system was neutralized with Na^+^/Cl^−^ ions. To mimic the physiological conditions, 0.15 M sodium chloride (NaCl) was added. The potential energy of the protein complex was minimized by employing the NPT ensemble. The molecular dynamics simulations were performed at 300 K temperature and 1 atm pressure for 100 ns, and NPT production ran under the OPLS4 force field. The models were relaxed before the simulation. The short-range electrostatic interactions were calculated using the particle mesh Ewald method^31^. The cutoff radius in the Coulomb interactions was 9.0 Å. The water molecules were explicitly described using the simple point charge model^32^. The Martyna–Tuckerman–Klein chain coupling scheme with a coupling constant of 2.0 ps was used for the pressure control, and the Nosé–Hoover chain coupling scheme^33^ was used for the temperature control. The trajectories were saved for examination after every 100 ps, and the simulation’s stability was verified by comparing the root mean square deviation (RMSD) of the protein complex over time. The projected changes in their conformation from the initial structure over the entire simulation period were expressed as root mean square deviation (RMSD) and root mean square fluctuation (RMSF) for MD simulations. A detailed description of the methodology can also be found elsewhere^34–37^. The radius of gyration (Rg) is calculated using GROMACS^38^. The Desmond trajectory is first converted to XTC (GROMACS) format via MDTraj^39^, and the calculation is conducted using the gmx gyrate command.

#### Principal component analysis (PCA)

Finding coordinated motions in a group of conformational structures obtained through experimental or molecular simulation data has been shown to be a useful application of principal component analysis (PCA). This method has been extensively employed to investigate the impact of conformational modifications on receptor function. We created the covariance matrix for PCA using the C-alpha atomic coordinates that we had collected from the molecular dynamic simulations. After that, a set of eigenvalues and eigenvectors (principal components) is obtained by diagonalizing this matrix. The coordinates of atoms are mapped to the principal components. GROMACS^38^ is utilized for this purpose using the XTC format of trajectory. The generation of covariance matrices and their diagonalization is achieved by the gmx covar command. The projection of the atomic coordinates to the principal components is done by the gmx anaeig command with the -proj parameter.

#### Free energy landscape (FEL)

Free energy landscape (FEL) is used to measure the change in energy during MD simulation of the protein as a function of principal components generated in PCA. This allows us to identify the metastable conformations of the protein in different systems and their relative energies. The energy function used is the Gibbs free energy. In this work, we used GROMACS^38^ for the analysis of FEL. The Gibbs free energy as a function of the first two principal components, PC1 and PC2, is evaluated using the gmx sham command. Locally estimated scatterplot smoothing (LOESS) regression is employed to smooth the FEL plots. The FEL plots are also verified by kernel density estimation (KDE) plots generated from the PCA results.

#### Dynamic cross-correlation matrix (DCCM)

Dynamic cross-correlation matrix (DCCM) can capture the correlation in motions of different residues. The motions between two residues can be correlated or anticorrelated, so a network of cross-correlation among residues can be formed^40^. We used the motions of the C-alpha atoms to represent those of the residues. The correlation between two residues $i$ and $j$ is measured by the cross-correlation coefficient $C\left(i,j\right)$:

$$C\left(i,j\right)=\frac{<{\varvec{\Delta}}{{\varvec{r}}}_{{\varvec{i}}}\cdot{\varvec{\Delta}}{{\varvec{r}}}_{{\varvec{j}}}>}{<{\left|{\varvec{\Delta}}{{\varvec{r}}}_{{\varvec{i}}}\right|}^{2}{>}^\frac{1}{2}<{\left|{\varvec{\Delta}}{{\varvec{r}}}_{{\varvec{j}}}\right|}^{2}{>}^\frac{1}{2}}$$

where ${\varvec{\Delta}}{{\varvec{r}}}_{{\varvec{i}}}$ is the displacement vector of the C-alpha atom of the $i$th residue, and the angle brackets represent the average values of the quantities inside them. $C\left(i,j\right)>0$, $C\left(i,j\right)<0$ and $C\left(i,j\right)=0$ are the indication of positive correlation, negative correlation (anti-correlated) and no correlation (uncorrelated), respectively.

In this work, we converted the Desmond trajectory to DCD format with MDTraj^39^ and we used the dccm function in the Bio3D package^41,42^ in R language to calculate DCCM.

#### MM/GBSA binding free energy of complex

Molecular mechanics/generalized Born surface area (MM/GBSA) is for the calculation of free energy of binding ($\Delta {G}_{bind}$) for the docked postures. It is defined as follows:

$$\Delta {G}_{bind}= {G}_{complex}-{G}_{ligand}-{G}_{receptor}$$

where the energy estimates of the optimized complex (complex), optimized free ligand (ligand), and optimized free receptor (receptor) are represented by the ${G}_{complex}$, ${G}_{ligand}$, and ${G}_{receptor}$, respectively.

Since MM/GBSA is more accurate than most molecular docking scoring systems, it is without a doubt one of the most used techniques for predicting binding free energy. Since MM/GBSA requires less computing power than other chemical free energy scoring methods, it is frequently employed in bimolecular studies involving protein folding, protein–ligand binding, and protein–protein interaction, among other topics. The accurate computation of the free energy, which powers molecular motions, is one of the most important issues in bimolecular studies. Using the MM/GBSA approach, it is possible to calculate the binding free energy by utilizing the solvation free energy differential upon binding and the interaction energy between the ligand and receptor complexes.

In this work, we used Uni-GBSA^43^ for the calculation of MM/GBSA binding energy. Uni-GBSA uses gmx_MMPBSA^44^ and MMPBSA.py in Amber^45^. After removing water molecules and ions, the frames in Desmond trajectory are split into separate PDB files. The pdbsplit function in Bio3D^41^ is used to separate the protein and the ligand into two PDB files. The ligand PDB files are converted to MOL2 format using Open Babel^46^. The protein PDB and the ligand MOL2 files are used as input for Uni-GBSA. The default configuration file (default.ini) is used except that the input pose process method is set as “input”, which means that the original input pose is used without energy minimization.

## Results and discussion

### Pangenome and pan-phylogeny analysis of *M. tuberculosis* genomes

**Figure 1:**
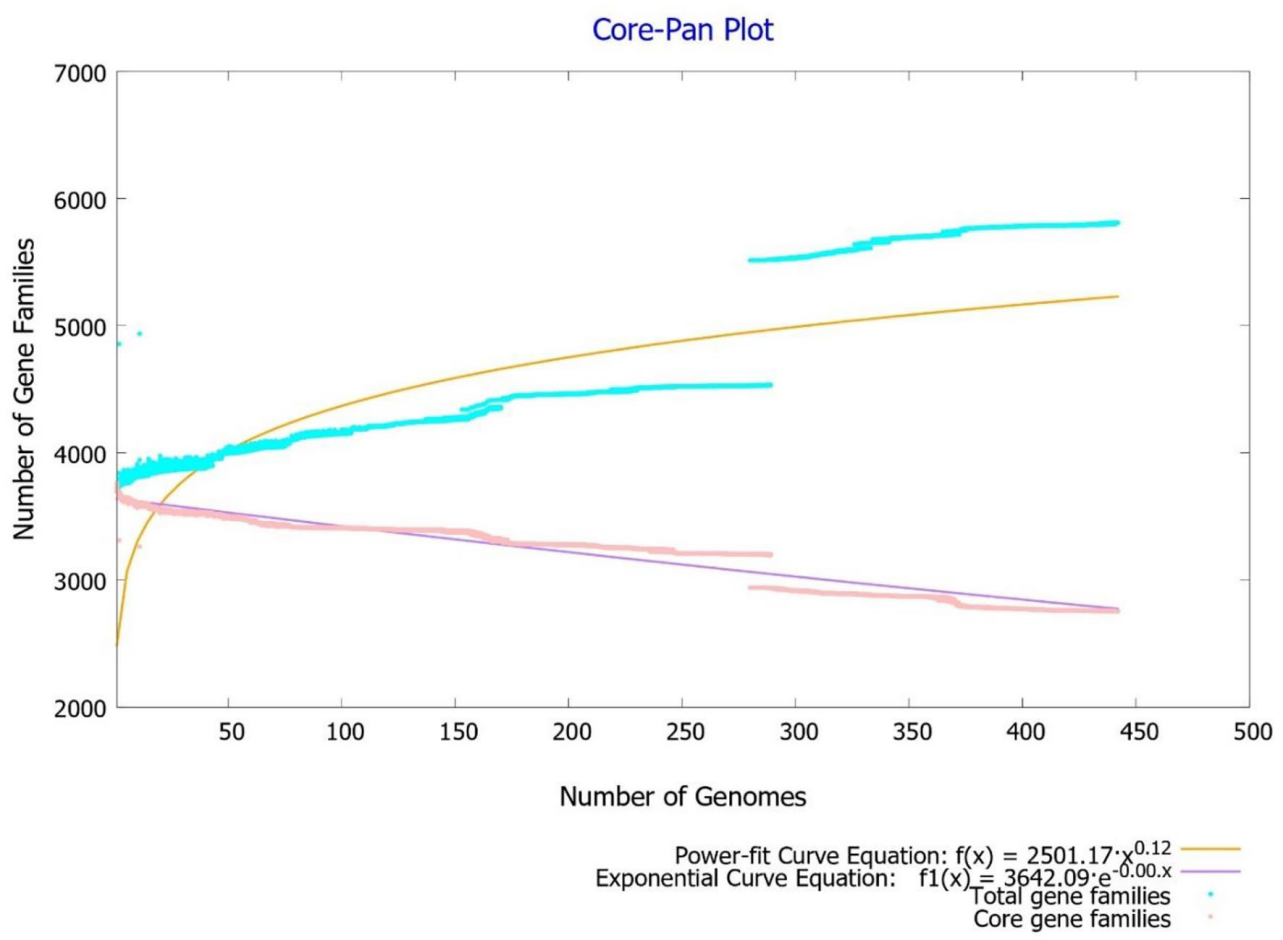
The pan-core plot, constructed using 442 *M. tuberculosis* genomes, demonstrates how the pangenome size increases while the core genome size decreases as genomes are added. It is essential to highlight that the pangenome curve reaches a level (in brown), signaling that significant alterations in the overall gene pool of this species are improbable in the future, suggesting that the pangenome is nearly closed, with limited potential for substantial genetic variations in *M. tuberculosis.*

A total of 442 complete *M. tuberculosis* genomes and their associated proteomes were obtained from the PATRIC database. Supplementary Table S1 provides comprehensive data, including accession numbers, strain names, genome statistics, isolation sources, the countries of isolation, and other information. A comprehensive pangenome analysis of these 442 complete *M. tuberculosis* genomes determined 5809 genes within the pangenome. Among these, 2754 proteins were identified as a core genome, representing genes consistently present across all studied genomes. The calculated pangenome size to core genome size ratio was 0.474, indicating that the core genome constitutes 47.4% of the entire pangenome. This observation underscores the limited genetic diversity observed among *M. tuberculosis* strains. This trend is graphically illustrated by the pan-core genome plot (Fig. 1), which also suggests that the global gene repertoire of this species is likely to remain relatively stable and resistant to significant changes in the future.

**Figure 2:**
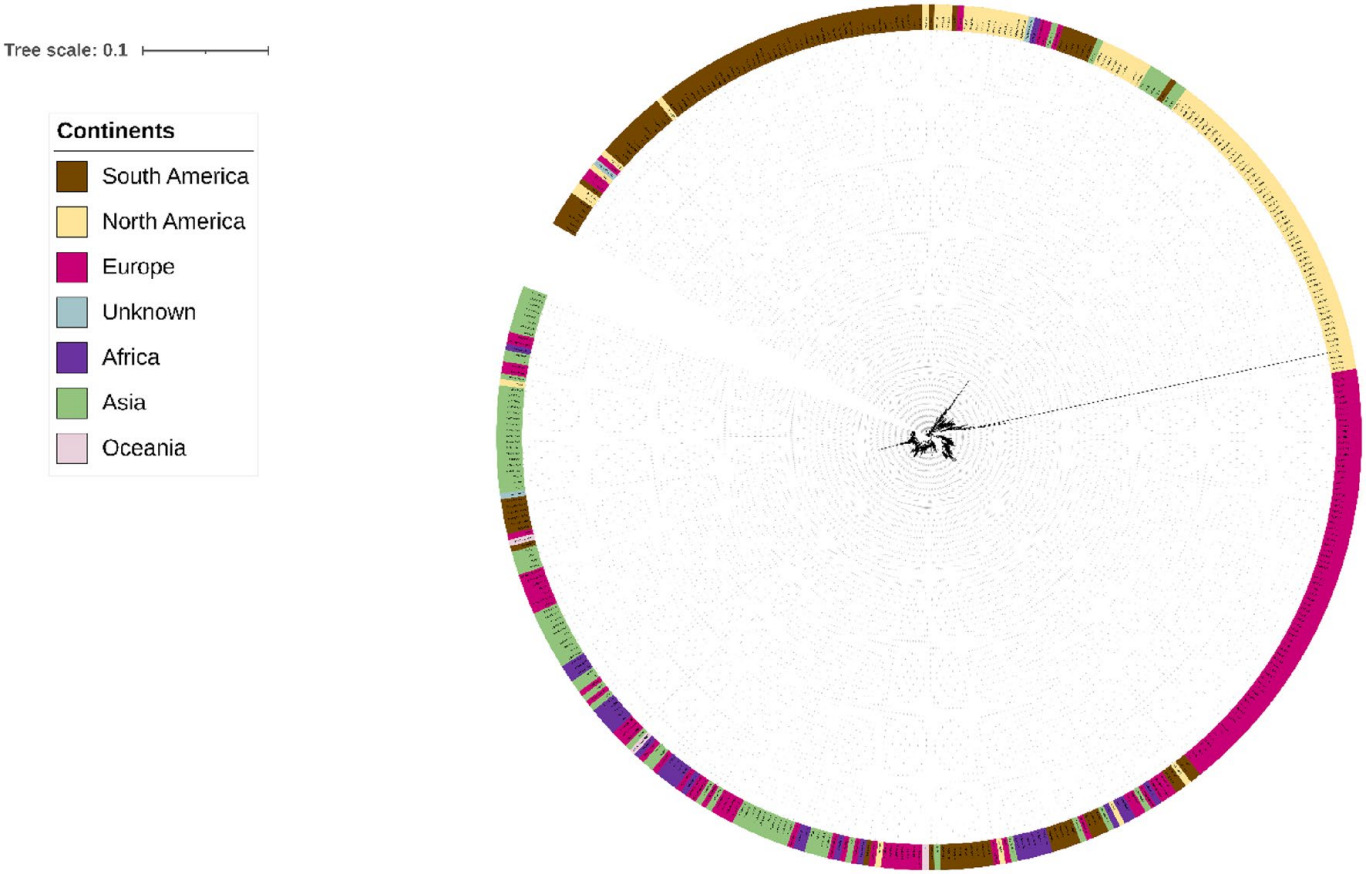
The pan-phylogeny tree, showcasing 442 *M. tuberculosis* genomes, is color-coded to represent the continents of origin for these strains. Interestingly, strains from different global regions tend to cluster into distinct branches within the tree. This tree is based on the presence or absence of accessory genes. It offers insights into the genetic relationships and evolutionary history of these *M. tuberculosis* strains, emphasizing the influence of geography on their genomic characteristics.

The power-law regression model’s “b” value of 0.121 shows that the pangenome is open. However, this value also suggests it may gradually transition towards a closed pangenome soon. Each genome, on average, contained approximately 3727 protein-encoding genes. The core genome, consisting of 2754 genes, accounted for a significant percent of the average genome size, 73.9%. Strain M0018684.2 had the lowest number of protein-encoding genes (3680), while strain MYC004 had the highest number (4462). Pan-matrix data was used to create the pan phylogeny, and the associated pangenome tree was produced using the neighbor-joining method with a default iteration value of 20. Figure 2 includes the pan-phylogeny-based phylogenetic tree and geographic source data to provide more information about the connections between these genomes.

### Subtractive proteomics revealed putative *M. tuberculosis* drug targets

Finding potential therapeutic targets started with subtractive proteomics performed on *M. tuberculosis* core proteins. This analysis involved assigning Rv numbers (gene identifiers specific to *M. tuberculosis*) and gene annotations to these core proteins. Supplementary Table S2 contains the dataset associated with the core genomes. A careful screening process was implemented to ensure the exclusion of proteins from human homologs, as human homologs could potentially interfere with host metabolism. Among the identified proteins, 2386 were classified as non-human homologous. These non-human homologs were subjected to further scrutiny, specifically assessing their essentiality. Among this subset, 685 proteins emerged as essential components for the pathogen’s survival. When essential proteins also exhibit functional characteristics associated with virulence, they become particularly noteworthy. Such proteins are pivotal in enabling bacteria to manipulate or break down host defense mechanisms, potentially contributing to pathogenesis^47^.

Consequently, the focus shifted to identifying genes linked to pathogenicity within the pool of 685 essential proteins. The VFDB has identified 113 virulence-related proteins necessary for *M. tuberculosis* pathogenicity (Supplementary File S1). Surprisingly, all 113 proteins are essential components linked to virulence and lack human homologs. This dataset has much potential and can be investigated for projects like creating a TB vaccine and novel therapeutic therapies. To specifically meet the objectives of this study, the size of the dataset was further reduced to pinpoint a few potential drug targets. This procedure makes designing drugs specifically suited to combat *M. tuberculosis* easier.

After extensive physiochemical analyses that included criteria like low molecular weights, a high aliphatic index, and a negative GRAVY score, 38 proteins were identified (Supplementary File S2). This group’s only six proteins were pinpointed through a comparative pathway analysis as part of distinct bacterial metabolic pathways. These six proteins were then subject to further scrutiny. Interestingly, among these proteins, those that played roles in multiple pathways (specifically, more than three pathways) were deliberately selected as potential drug targets for *M. tuberculosis*. This selection approach was taken to minimize the risk of inadvertently targeting any human pathways during drug therapy, as detailed in Table 1.

**Table 1:** Metabolic pathway analysis of potential drug targets.

Protein name	Rv number	K0 num	KEGG pathway
Pantothenate synthetase	Rv3602c	K01918	map00410 beta-Alanine metabolism
			map00770 Pantothenate and CoA biosynthesis
			map01100 Metabolic pathways
			map01110 Biosynthesis of secondary metabolites
Isocitrate lyase	Rv0467	K01637	map00630 Glyoxylate and dicarboxylate metabolism
			map01100 Metabolic pathways
			map01110 Biosynthesis of secondary metabolites
			map01120 Microbial metabolism in diverse environments
			map01200 Carbon metabolism

In the final stage of our analysis, the top 2 proteins (isocitrate lyase and pantothenate synthetase) were focused. The similarity of these targets with drugs in the DrugBank database was evaluated. This step aimed to determine whether these proteins are potentially druggable. Our investigation revealed that the selected targets were druggable with experimental drugs available in the drug bank database but not with FDA-approved drugs. This finding suggests that these proteins could serve as promising novel drug targets. All FDA-approved drugs (*n* = 1613) were retrieved from the ZINC database, and docking experiments were conducted with the selected proteins to explore their druggability further. The ligands with the lowest binding scores were then determined, and their fit quality scores were also evaluated (Table 2). The fit quality scores are near or exceed 1, which is indicative of optimal binding.

**Table 2:** Drug targets’ AutoDock Vina scores with their ligands and their fit quality scores.

Proteins	Rv number	ZINC ID	Binding affinity (kcal/mol)	Fit quality score
Isocitrate lyase	Rv0467	ZINC3978005 (Dihydroergotamine)	− 10.3	1.082
		ZINC164528615 (Glecaprevir)	− 10	1.334
		ZINC52955754 (Ergotamine)	− 9.9	1.04
		ZINC11679756 (Eltrombopag)	− 9.9	0.98
		ZINC6716957 (Nilotinib)	− 9.9	1.005
		ZINC150588351 (Elbasvir)	− 9.8	1.553
Pantothenate synthetase	Rv3602c	ZINC3797541 (Zytiga)	− 10.4	1.052
		ZINC1529323 (Methotrexate)	− 10.2	1.01
		ZINC607986 (SCHEMBL12375910)	− 10.1	1.004
		ZINC1999441 (Nebivolol)	− 10.1	1.004
		ZINC3810860 (Zetia)	− 10.1	1.001
		ZINC538564 (Casodex)	− 9.9	0.985

On these two proteins from Table 2, literature studies were done to determine their applicability as drug targets. Isocitrate lyase is a vital enzyme in the metabolism of *M. tuberculosis*. Importantly, they are absent in mammals, making them valuable targets for TB treatment^48^. Isocitrate lyase is pivotal in the glyoxylate cycle, a metabolic pathway bypassing several Krebs cycle steps. It converts isocitrate into succinic acid and glyoxylate, which generate energy^49,50^. Additionally, pyruvate is converted to alanine by the upregulated alanine dehydrogenase, while glycine dehydrogenase converts glyoxylate into glycine while oxidizing NADH to NAD^51^. Glyoxylate and pyruvate are produced by the glyoxylate, and the methyl citrate cycles, respectively, which are necessary building blocks for these reactions. These cycles are catalyzed by two isomers of isocitrate lyase, ICL1 and ICL2, encoded by the genes icl and aceA, respectively^52^. Notably, when either ICL or aceA genes are deleted, *M. tuberculosis* exhibits reduced virulence and impaired survival in activated macrophages^53^. *M. tuberculosis* cannot survive in the latent TB model without isocitrate lyase. Due to the host’s absence of these metabolic pathways, *M. tuberculosis* isocitrate lyase is a desirable drug target for treating latent bacteria*.* Consequently, isocitrate lyase has gained significant attention as a potential target for future research and drug development^54–56^.

Pantothenate synthetase in *M. tuberculosis* is a crucial enzyme in pantothenate biosynthesis. Its crystal structure reveals that AMP and β-alanine are involved in its active site, with β-alanine binding after pantoyl adenylate formation^57,58^. Pantothenate synthetase is crucial for *M. tuberculosis* survival in the host, and recent findings show that pyrazinamide, a common antitubercular drug, also inhibits pantothenate biosynthesis. Confirmation of this mechanism of action is provided by mutations in the panD gene identified in pyrazinamide-resistant MTB strains. This finding highlights the pantothenate biosynthesis pathway as a potentially effective target for developing new TB drugs^59–62^. These enzymes may be suitable for designing or selecting inhibitors because they are not found in mammals. Given these insights, Pantothenate synthetase emerges as a promising antimycobacterial target, potentially effective against non-replicating persistent forms of MTB^63,64^.

### Docking analyses of drug targets revealed potential lead compounds for drug discovery

**Figure 3:**
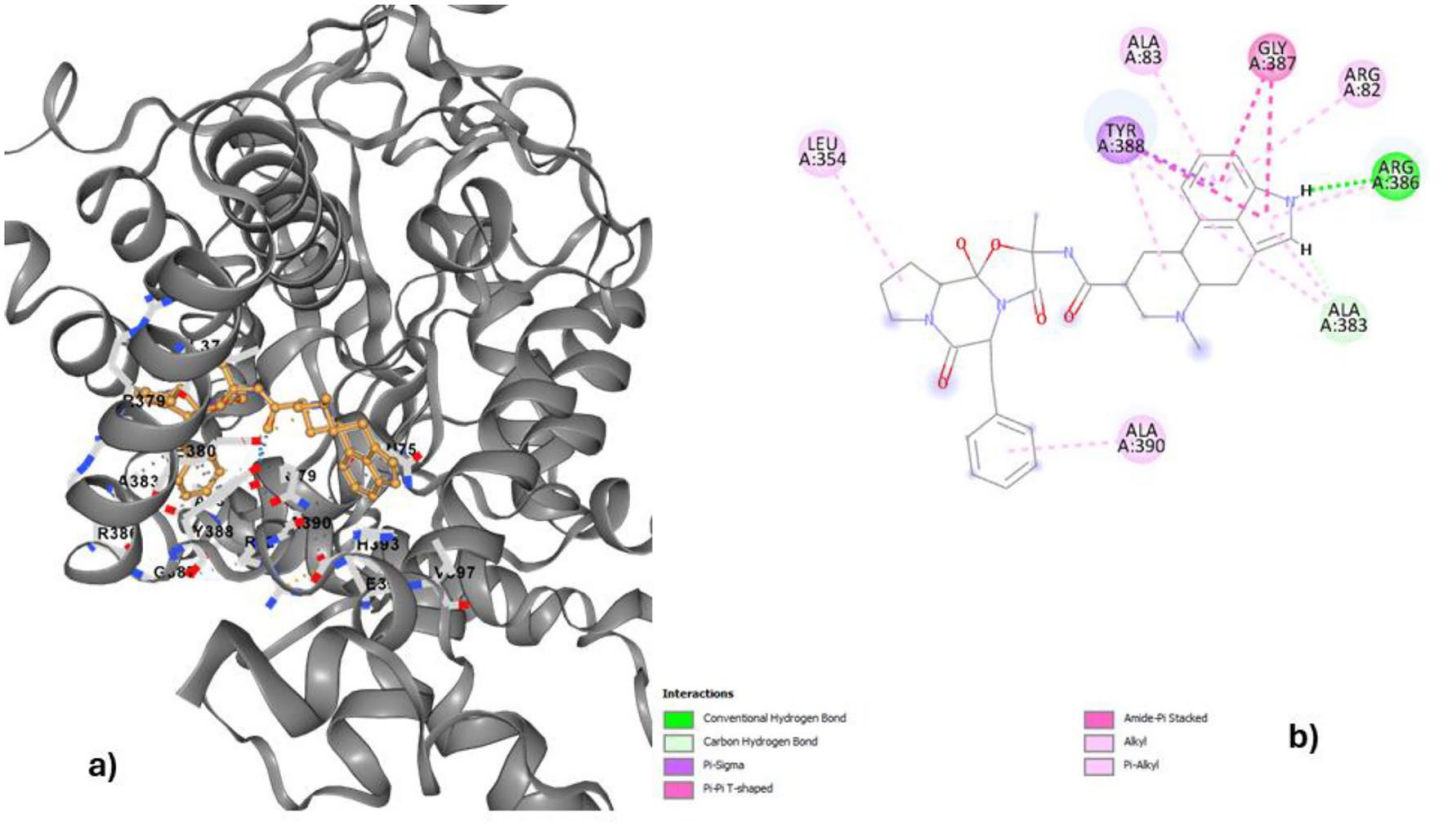
The docked complex of dihydroergotamine and isocitrate lyase.

The results of the AutoDock Vina docking of ligands with drug targets are shown in Table 2. Dihydroergotamine (ZINC000003978005) is the highest-ranked ligand for isocitrate lyase. The binding energy of this substance to isocitrate lyase was − 10.3 kcal/mol (Fig. 3a). ZINC000164528615 ligand shows the second highest binding affinity (− 10 kcal/mol) with isocitrate lyase, followed by Ergotamine (ZINC000052955754), which shows − 9.9 kcal/mol binding affinity.

Dihydroergotamine (ZINC3978005) exhibited the highest binding affinity (− 10.3) for isocitrate lyase. Moreover, the docking interactions that formed for this complex demonstrated various types of bonds, such as conventional hydrogen bonds, carbon hydrogen bonds, pi-sigma, pi-pi T-shaped, amide-Pi stacked, alkyl, and pi-alkyl bonds. The aminoacids in contact with these bonds were leucine, tyrosine, alanine, glycine, and arginine (Fig. 3b). These variety of interactions suggests a strong and stable binding between dihydroergotamine and isocitrate lyase^65^. The presence of multiple types of bonds indicates a complex and intricate molecular interaction, potentially leading to a more efficient inhibition of the enzyme’s activity^66^. Overall, the detailed analysis of the docking interactions provides valuable insights into the mechanism of action of dihydroergotamine as a potential inhibitor of isocitrate lyase. By targeting this enzyme, dihydroergotamine may offer a promising approach for developing new antibiotics to address bacterial infections^67^.

Dihydroergotamine has the highest binding affinity with isocitrate lyase and is an FDA-Approved drug mainly used to treat cluster and migraine headaches. The drug belongs to the subclass of ergot alkaloids. Dihydroergotamine treats migraine and cluster headaches by constricting blood vessels in the brain to relieve pain^68^. DHE can be administered via several routes, including intravenous, intramuscular, subcutaneous, intranasal, and oral. Studies have shown that dihydroergotamine can effectively relieve migraine pain and associated symptoms like nausea and sensitivity to light and sound^69,70^. It is particularly useful in cases of severe or refractory migraines. Dihydroergotamine is contraindicated in patients with uncontrolled hypertension, coronary artery disease, peripheral vascular disease, and hepatic or renal impairment. It should not be used concomitantly with other ergot alkaloids or potent CYP3A4 inhibitors. Due to its vasoconstrictive properties, DHE should be used cautiously in patients with cardiovascular risk factors. Regular monitoring of blood pressure and cardiac function may be necessary during treatment. DHE can interact with several medications, including certain antidepressants, antifungals, and protease inhibitors^71^. These interactions can affect the metabolism and effectiveness of dihydroergotamine, necessitating dose adjustments or alternative treatments.

**Figure 4:**
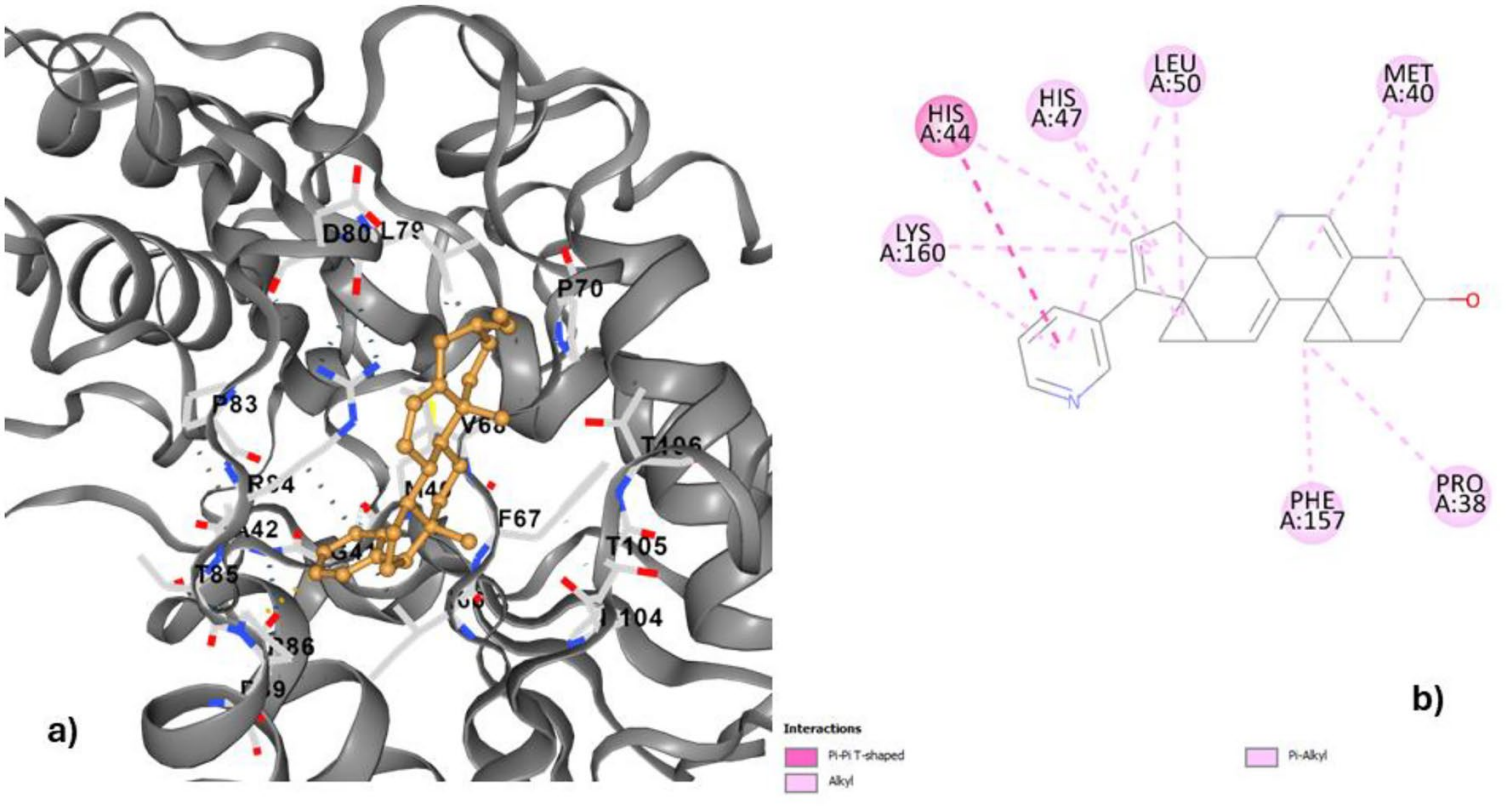
The docked complex of abiraterone acetate and pantothenate synthetase.

Analysis of drug-ligand interactions showed that Abiraterone acetate/Zytiga (ZINC000003797541) had a higher binding energy (− 10.4 kcal/mol) to pantothenate synthetase (Fig. 4a). Another ligand, Methotrexate (ZINC000001529323), shows the second highest binding affinity (− 10.2 kcal/mol), followed by ZINC000000607986, showing − 10.1 kcal/mol binding affinity.

Abiraterone acetate—pantothenate synthetase docked complex showed interactions of pi-pi-T-shaped, alkyl and pi-alkyl bonds in contacts with different amino acids of the receptor protein, such as lysine, histidine, leucine, methionine, phenylananine and proline (Fig. 4b). This suggests that Abiraterone acetate/Zytiga could be a promising lead compound for drug discovery targeting pantothenate synthetase. The many amino acids interacting indicates that the drug-ligand complex has the potential for strong and specific binding, which is crucial for effective drug design^72,73^. Further research and development on this interaction could lead to the development of new and improved treatments for diseases or conditions related to pantothenate synthetase. The diverse nature of the interactions also suggests that Abiraterone acetate/Zytiga may have a multi-targeted approach, making it a versatile candidate for therapeutic applications^74^. In addition, Alkyl bonds suggest that Abiraterone acetate/Zytiga has the potential to be stable and long-lasting within the body, which is essential for consistent and effective treatment^75^. In a similar study Rajan et al. 2023 proved the inhibition of pantothenate synthetase with seaweed metabolites showing potential inhibitory properties, however the best binding energies obtained were − 5.547 kcal/mol and − 2.73 kcal/mol, which were not as strong as the interactions observed with Abiraterone acetate/Zytiga. This further highlights the potential of Abiraterone acetate/Zytiga as a promising candidate for therapeutic use in the treatment of certain conditions. The stability, multi-targeted approach, and strong interactions observed with this compound make it a stand-out option for further research and development in the medical field.

Abiraterone acetate, which has the highest binding affinity, is an FDA-approved drug used to treat advanced prostate cancer. Abiraterone acetate is a steroidal cytochrome P450 17α-hydroxylase-17,20-lyase (CYP17) inhibitor. It inhibits androgen biosynthesis, specifically targeting androgen synthesis within the testes, adrenal glands, and prostate tumor tissue. This inhibition ultimately suppresses androgen-sensitive prostate cancer growth. Abiraterone acetate is indicated in combination with prednisone for the treatment of metastatic castration-resistant prostate cancer (mCRPC) in patients who have received prior chemotherapy containing docetaxel and for metastatic castration-sensitive prostate cancer (mCSPC). It is available in oral tablet form. Common adverse effects include hypertension, hypokalemia, fluid retention, and liver function test abnormalities. Abiraterone acetate is metabolized primarily by hepatic CYP3A4 enzymes^76–78^.

### MD simulations

Molecular dynamics simulations were performed on the top hits having high binding energies. Over the simulation period, the projected conformational changes from the initial structure were presented in terms of root mean square deviation (RMSD). Moreover, structural stability, atomic mobility, and residue flexibility at times of interaction of protein-hit were expressed with root mean square fluctuation (RMSF) values. We also analyzed the change in secondary structures of the proteins during MD simulations. Principal component analysis (PCA) and free energy landscape (FEL) are conducted for the investigation of conformational changes in the proteins. Dynamic cross-correlation matrix (DCCM) is calculated for identification of the patterns of correlation of motions of residues. Finally, molecular mechanics/generalized Born surface area (MM/GBSA) is utilized to study the binding energy of the protein–ligand complexes in the course of MD simulation.

#### Isocitrate lyase with dihydroergotamine

**Figure 5:**
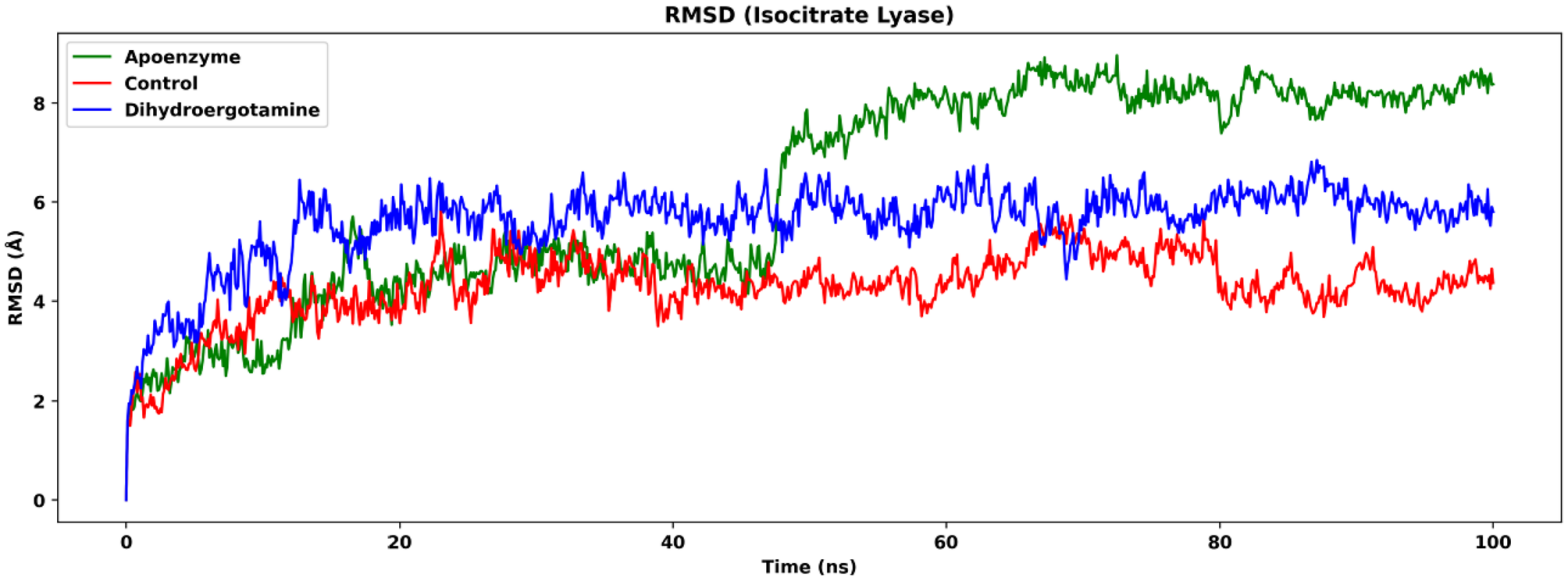
Root mean square deviation (RMSD) of isocitrate lyase in three systems, namely Apoenzyme (apoenzyme system), Control (isocitrate lyase-ZINC2111081 complex) and Dihydroergotamine (isocitrate lyase-dihydroergotamine complex). Only the C-alpha atoms are considered.

Molecular dynamics (MD) simulation is performed for isocitrate lyase for three systems, the apoenzyme, the control system and the isocitrate lyase-dihydroergotamine complex. The control system is the isocitrate lyase-ZINC2111081 complex, where ZINC2111081 was previously shown to be an effective drug that inhibits isocitrate lyase^79^. The root mean square deviation (RMSD) plot, in which only C-alpha atoms are considered, shows that all three systems attain equilibrium after 50 ns (Fig. 5). The apoenzyme system has the highest value, followed by the protein-dihydroergotamine complex system, and the control system has the lowest value (after 50 ns, apoenzyme: mean: 8.23 Å, SD: 0.275 Å; protein-dihydroergotamine: mean: 5.94 Å, SD: 0.358 Å; control: mean: 4.61 Å, SD: 0.461 Å). The difference in RMSD between the two ligand-bound protein systems is less significant than that between the apoenzyme and the ligand-bound protein systems. This indicates that the fluctuation of the protein system is the highest for the apoenzyme, and the binding of drugs lowers the degree of fluctuation of the enzyme probably due to the interactions between the protein and the drugs that hinder the motions of the residues, which in turn might affect the normal function of the enzyme.

**Figure 6:**
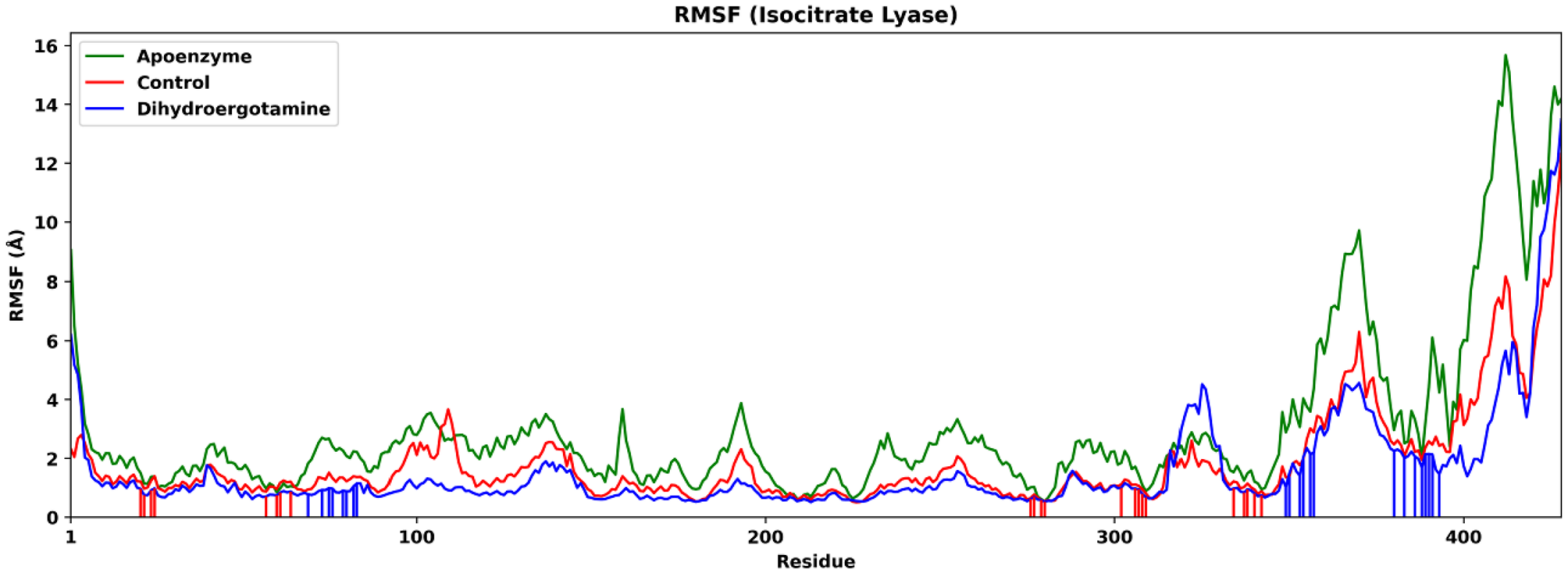
Root mean square fluctuation (RMSF) of isocitrate lyase in three systems, namely Apoenzyme (apoenzyme system), Control (isocitrate lyase-ZINC2111081 complex) and Dihydroergotamine (isocitrate lyase-dihydroergotamine complex). Only the C-alpha atoms are considered. The red and blue vertical lines represent the residues that interact with the ligand in the Control and Dihydroergotamine systems, respectively. Only the C-alpha atoms are considered.

The degree of fluctuation of various residues in the three systems is shown in the RMSF plot (Fig. 6). The regions near the C-terminus fluctuate the most for all systems. The overall RMSF is highest for the apoenzyme system and is lower for the two ligand-bound systems, the difference in RMSF between which is less significant, agreeing with the results of RMSD. For the control system, the ZINC2111081 ligand interacts with the isocitrate lyase via Pro21–Asp25, Trp57–Glu64, Ala276–Asp280, Lys302–Met309 and Lys334–Lys342. For the protein-dihydroergotamine complex system, the dihydroergotamine ligand interacts with the enzyme via Leu69–Ala83, Ala349–Ser357 and Glu380–His393. It should be noted that the decrease in the degree of fluctuation is not localized at the residues of interactions but is contributed by almost every part of the protein, which indicates that the interactions of the residues with the drugs have effects on other segments of the enzyme, probably by hindering the motions the residues of interactions, which in turn affects the movements of residues that are further away via the peptide chain or hydrogen bonds.

**Figure 7:**
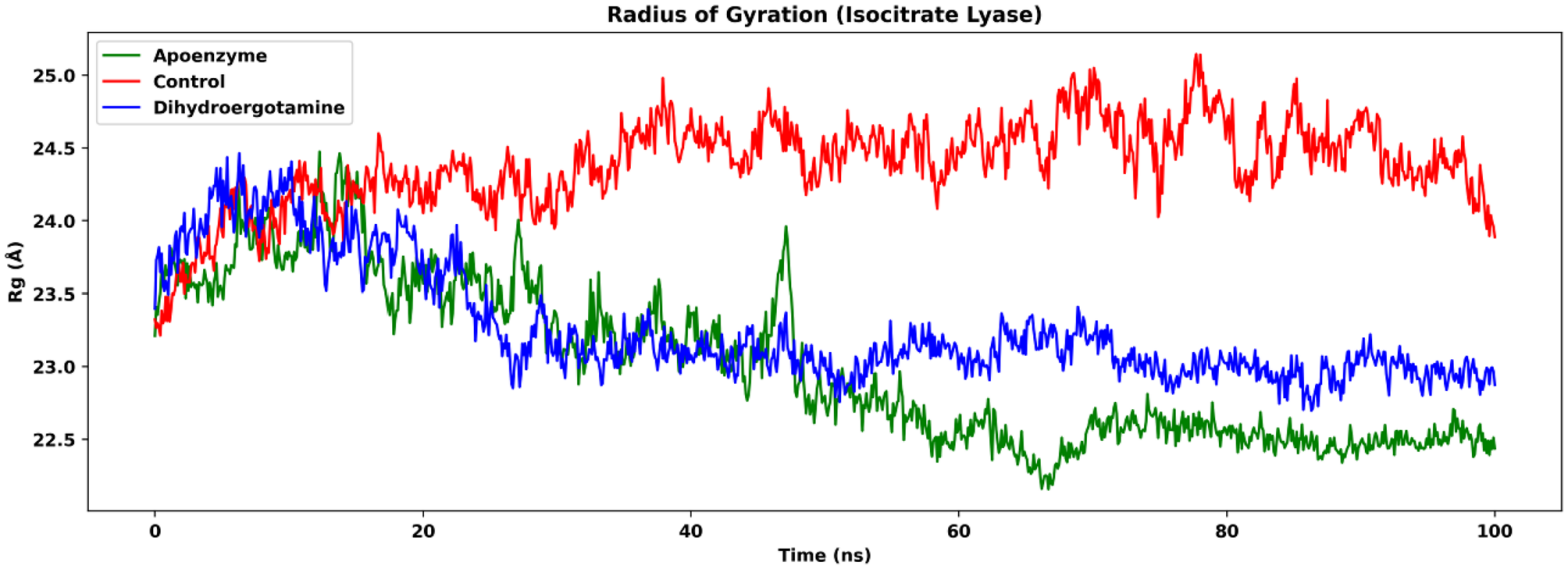
The radius of gyration (Rg) of isocitrate lyase in three systems, namely Apoenzyme (apoenzyme system), Control (isocitrate lyase-ZINC2111081 complex) and Dihydroergotamine (isocitrate lyase-dihydroergotamine complex). Only the protein backbone is considered.

We also analyzed the radius of gyration (Rg) of the simulation of the three systems, which shows the degree of compactness of the protein (Fig. 7). The apoenzyme has the lowest Rg among the three systems, which suggests that it folds the fastest during the simulation and is more compact. For the two ligand-bound protein systems, the control one has higher Rg then the dihydroergotamine one (after 50 ns, apoenzyme: mean: 22.5 Å, SD: 0.137 Å; protein-dihydroergotamine: mean: 23.0 Å, SD: 0.129 Å; control: mean: 24.5 Å, SD: 0.217 Å). This indicates that the binding of drug molecules lowers the compactness of isocitrate lyase, which could be due to the formation of hydrogen bonds between the drug and the residues which expand the protein. The lower compactness of the enzyme-drug complex might suggest a destabilization effect.

**Figure 8:**
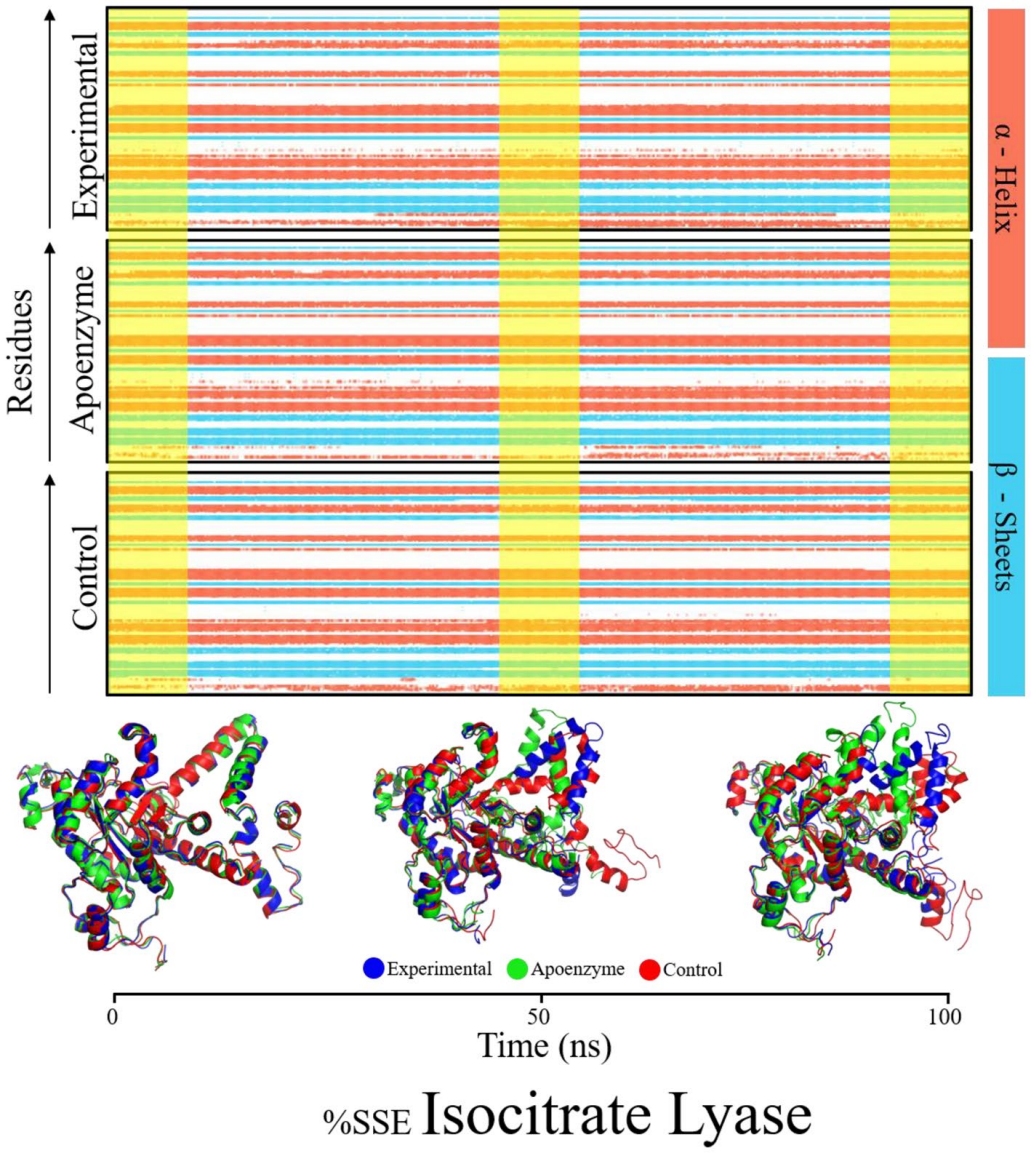
%age secondary structure timeline of isocitrate lyase in three systems, namely Apoenzyme (apoenzyme system), Control (isocitrate lyase-ZINC2111081 complex) and Dihydroergotamine (isocitrate lyase-dihydroergotamine complex).

The % secondary structure plot offers a comprehensive overview of the temporal evolution of secondary structure elements in isocitrate lyase in the three systems of the apoenzyme, the control system and the protein-dihydroergotamine complex, supported by aligned structural snapshots at 0, 50, and 100 ns (Fig. 8). All simulations showcase prevalent alpha helices, followed by beta strands, in line with typical protein structural patterns. Notably, isocitrate lyase with ligand demonstrates the highest total secondary structure elements at 50.47%, dominated by alpha helices at 40.53% and slightly lower beta strands at 9.94%, suggesting a potential stabilizing effect of the ligand on helical elements. In comparison, the apoenzyme displays slightly lower percentages of both alpha helices (39.67%) and beta strands (9.63%). This could be a reason for the higher level of fluctuation of the apoenzyme system according to the RMSD results. The control simulation exhibits a marginally lower percentage of alpha helices (39.45%) and slightly higher beta strands (10.26%). The timeline plot visually illustrates the stability of the isocitrate lyase with ligand complex, while apoenzyme and control simulations show comparable trends, emphasizing shared characteristics in the absence of the ligand.

**Figure 9:**
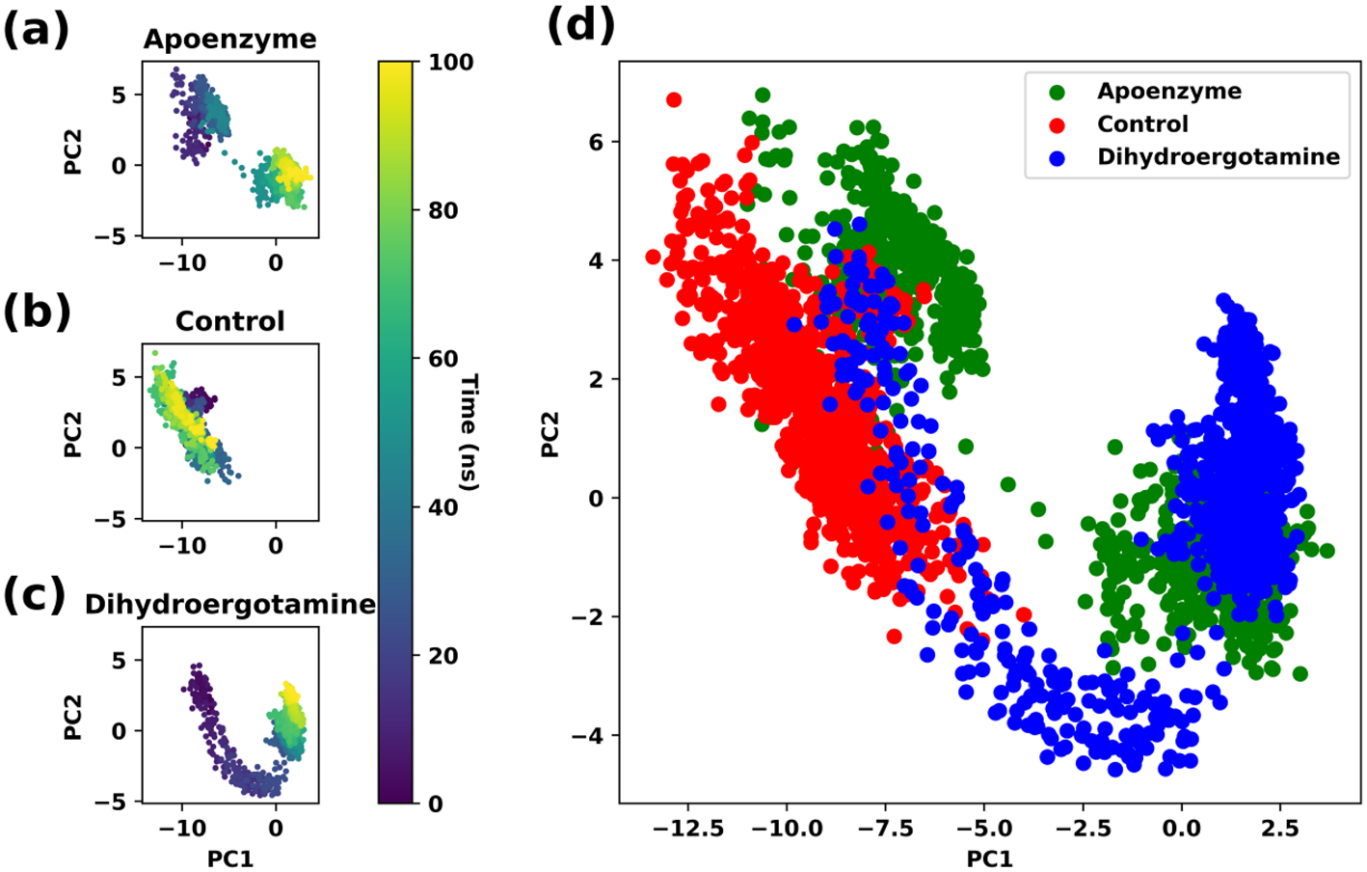
Principal component analysis (PCA) of isocitrate lyase in three systems, namely Apoenzyme (apoenzyme system, **a**), Control (isocitrate lyase-ZINC2111081 complex, **b**) and Dihydroergotamine (isocitrate lyase-dihydroergotamine complex, **c**), and (**d**) the overlaid PCA plot of three systems. Only the C-alpha atoms are considered.

Principal component analysis (PCA) is conducted for analysis of the trajectories of the three systems. The C-alpha coordinates of all protein systems are projected to the principal components generated with the isocitrate lyase-dihydroergotamine system. From the PCA plots, there are substantial differences in their trajectories and conformational changes for the three systems during simulation (Fig. 9). Considering the apoenzyme system, it occupies two regions in the PCA plot. There is some overlapping between the apoenzyme and control systems and between the apoenzyme and protein-dihydroergotamine ones. However, there are regions that are reachable by the apoenzyme system but not by the control and protein-dihydroergotamine ones, which indicates that the drugs hinder the motion and conformational changes of the protein. The overlapping between the control and protein-dihydroergotamine systems is small, suggesting that the dihydroergotamine has a different inhibition mechanism when compared to ZINC2111081.

**Figure 10:**
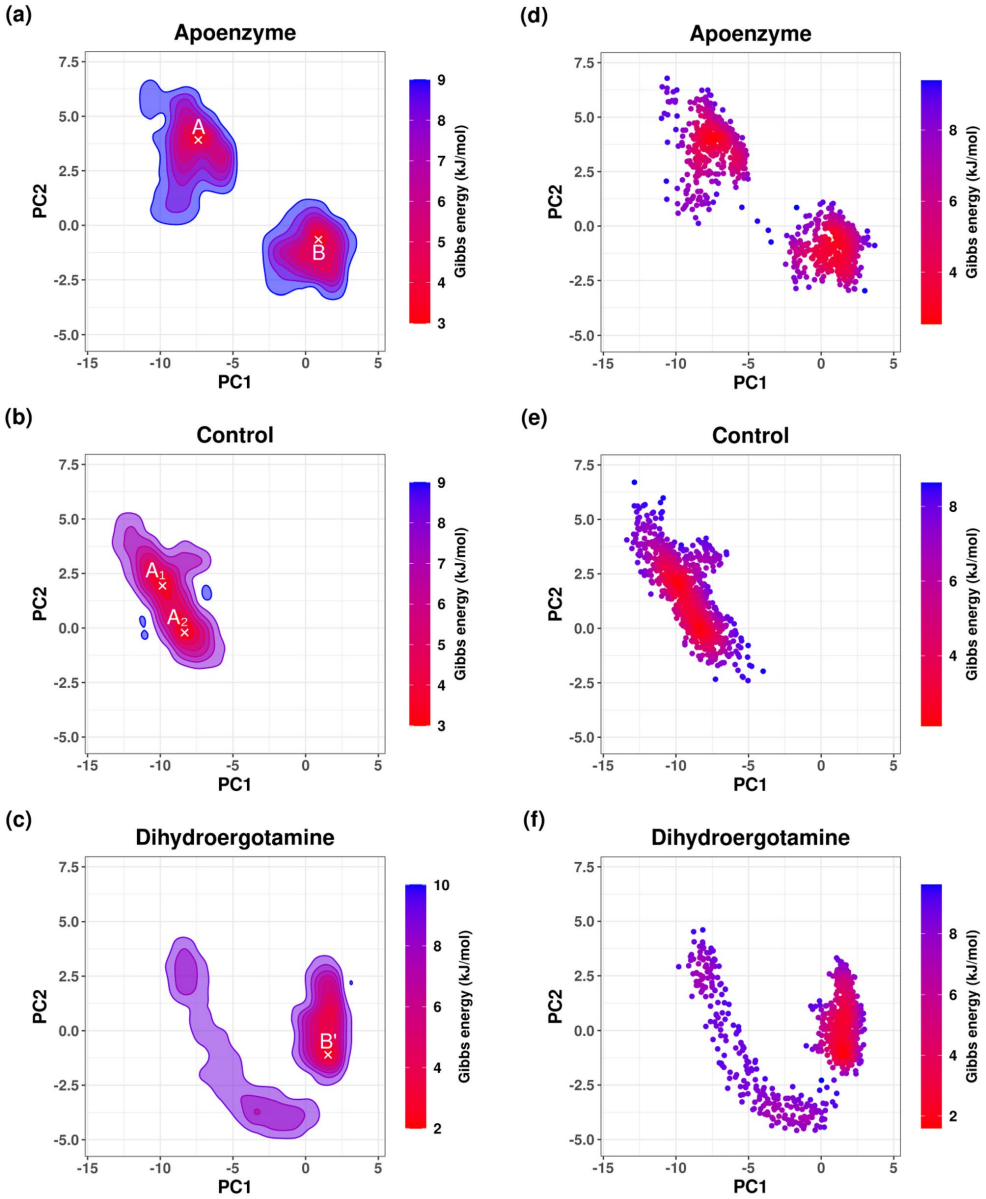

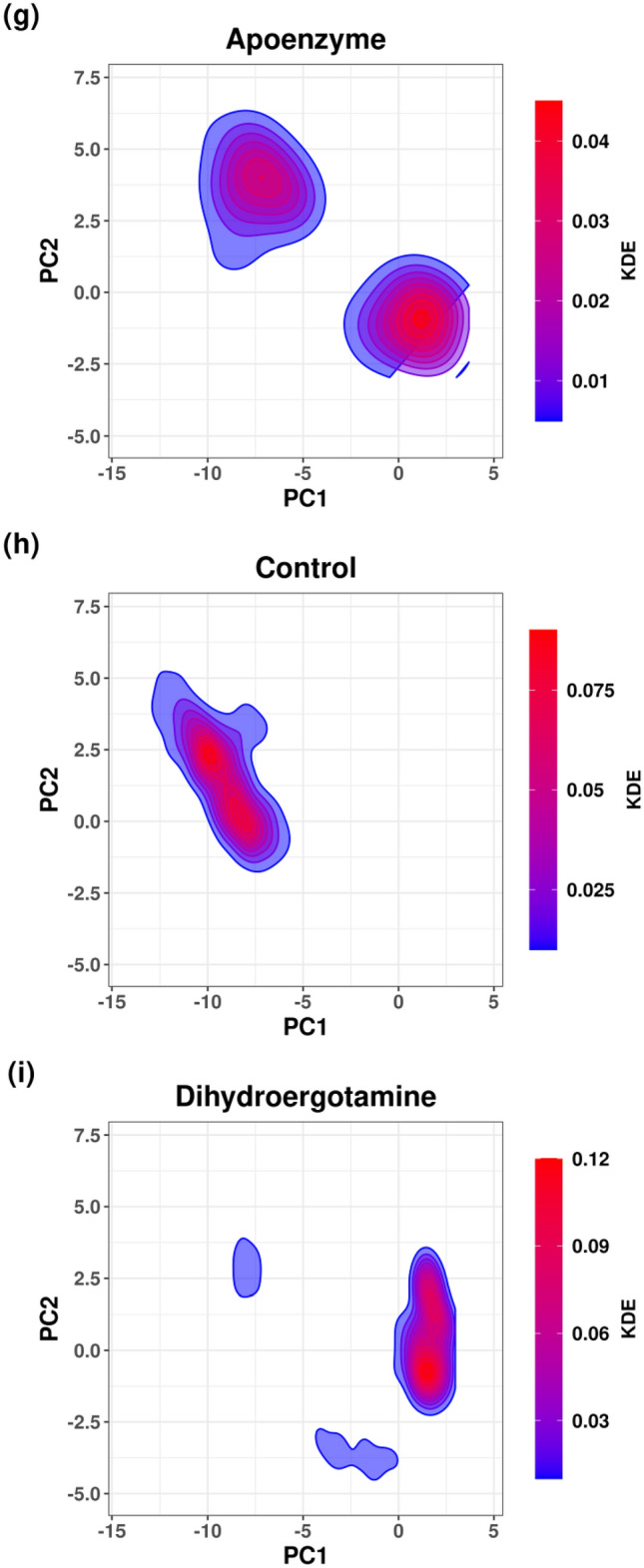
Free energy landscape (FEL) of isocitrate lyase in three systems, namely Apoenzyme (apoenzyme system, **a**), Control (isocitrate lyase-ZINC2111081 complex, **b**) and Dihydroergotamine (isocitrate lyase-dihydroergotamine complex, **c**). PCA results colored by Gibbs free energy of Apoenzyme (**d**), Control (**e**) and Dihydroergotamine (**f**). Kernel density estimation (KDE) plots of PCA results of Apoenzyme (**g**), Control (**h**) and Dihydroergotamine (**i**).

Free energy landscape (FEL) is plotted using the results of PCA. It is for the analysis of the metastable conformations of the isocitrate lyase in the three systems, and it is verified by the kernel density estimation (KDE) plots (Fig. 10). In the apoenzyme system, two energy minima can be observed, indicating the existence of two metastable conformers (conformers A and B). The control system shows two energy minima which correspond to two conformers (conformers A_1_ and A_2_). The conformers A_1_ and A_2_ are closer to conformer A than B in the apoenzyme system in terms of position, which suggests that A_1_ and A_2_ should be more similar to A. The protein-dihydroergotamine system shows one energy minimum, and there is one metastable conformer (conformer B’). The position of B’ is closer to that of B than A in the apoprotein system, which suggests that B’ is more similar to B. From these results, the functional apoenzyme has two conformers, and it can be deduced that the binding of drugs inhibits the protein by locking it in one specific conformer. It can be also shown that the inhibition mechanisms of ZINC2111081 and dihydroergotamine are different because they lock the protein at two different conformers (conformer A for ZINC2111081; conformer B for dihydroergotamine).

**Figure 11:**
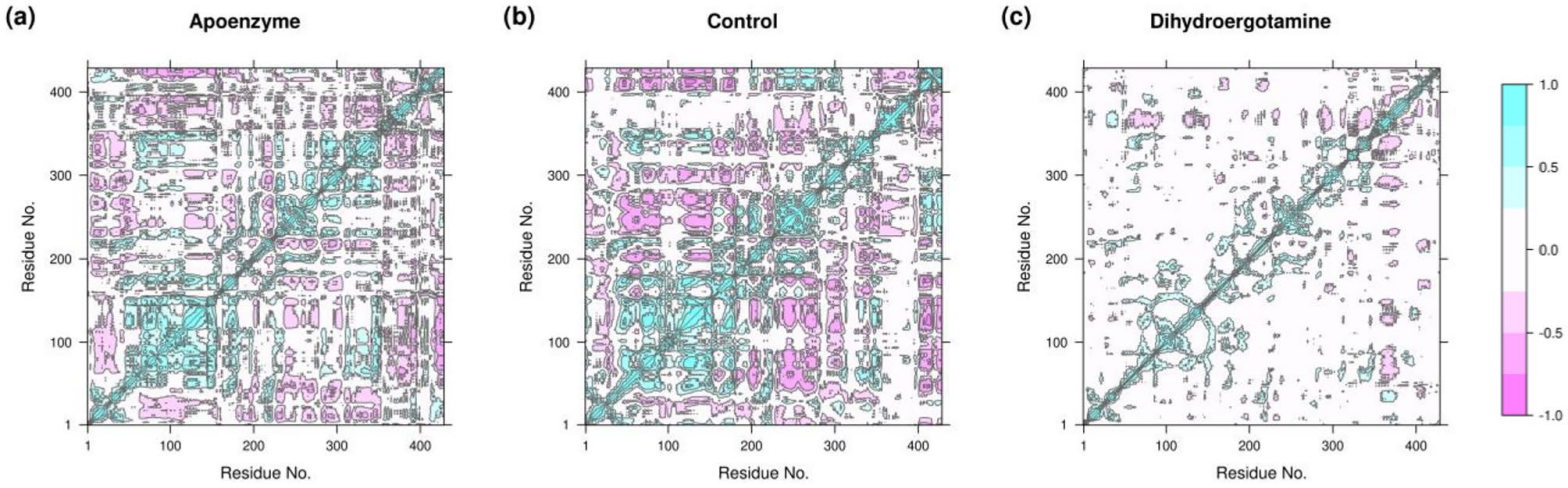
Dynamic cross-correlation matrix (DCCM) of isocitrate lyase in three systems, namely (**a**) Apoenzyme (apoenzyme system), (**b**) Control (isocitrate lyase-ZINC2111081 complex), and (**c**) Dihydroergotamine (isocitrate lyase-dihydroergotamine complex).

To analyze the correlation of motions of residues in the three protein systems, dynamic cross-correlation matrix (DCCM) is calculated. The RMSD results show that the three systems attain equilibrium after 50 ns, so we extracted those frames for DCCM analysis. It can be observed that the drug-bound protein systems show very different patterns when compared with the apoenzyme (Fig. 11). The binding of ZINC2111081 to isocitrate lyase changes its correlation pattern, but the number of correlated motions is still substantial. In contrast, the binding of dihydroergotamine to the enzyme ruins most of the correlated motions, possibly indicating that dihydroergotamine is a more effective drug. This might also indirectly suggest that the mechanisms of inhibition are not the same for both drugs, which supports the deduction from the analysis of PCA and FEL.

**Figure 12:**
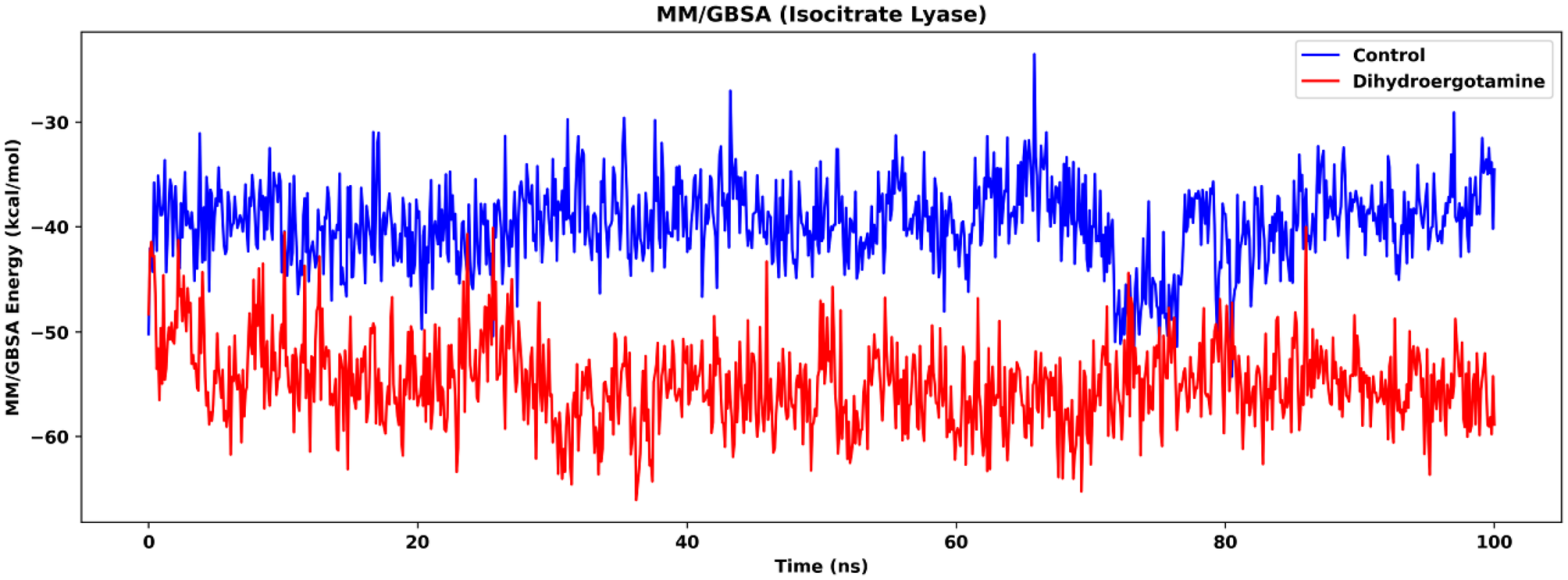
MM/GBSA energy of isocitrate lyase in the two ligand-bound systems, namely Control (isocitrate lyase-ZINC2111081 complex) and Dihydroergotamine (isocitrate lyase-dihydroergotamine complex).

Molecular mechanics/generalized Born surface area (MM/GBSA) is employed to evaluate the binding energy of isocitrate lyase and the two drugs (ZINC2111081 and dihydroergotamine). The MM/GBSA results (Fig. 12) show that the isocitrate lyase-dihydroergotamine complex is lower than the isocitrate lyase-ZINC2111081 complex in terms of binding energy during simulation (after 50 ns, isocitrate lyase-dihydroergotamine complex: mean: − 55.4 kcal/mol, SD: 3.59 kcal/mol; isocitrate lyase-ZINC2111081 complex: mean: − 39.8 kcal/mol, SD: 4.21 kcal/mol), suggesting that the protein-dihydroergotamine complex is more stable. This also suggests that dihydroergotamine might be a more effective drug when compared to ZINC2111081, which is supported by the results of the DCCM analysis.

#### Pantothenate synthetase with abiraterone acetate

**Figure 13:**
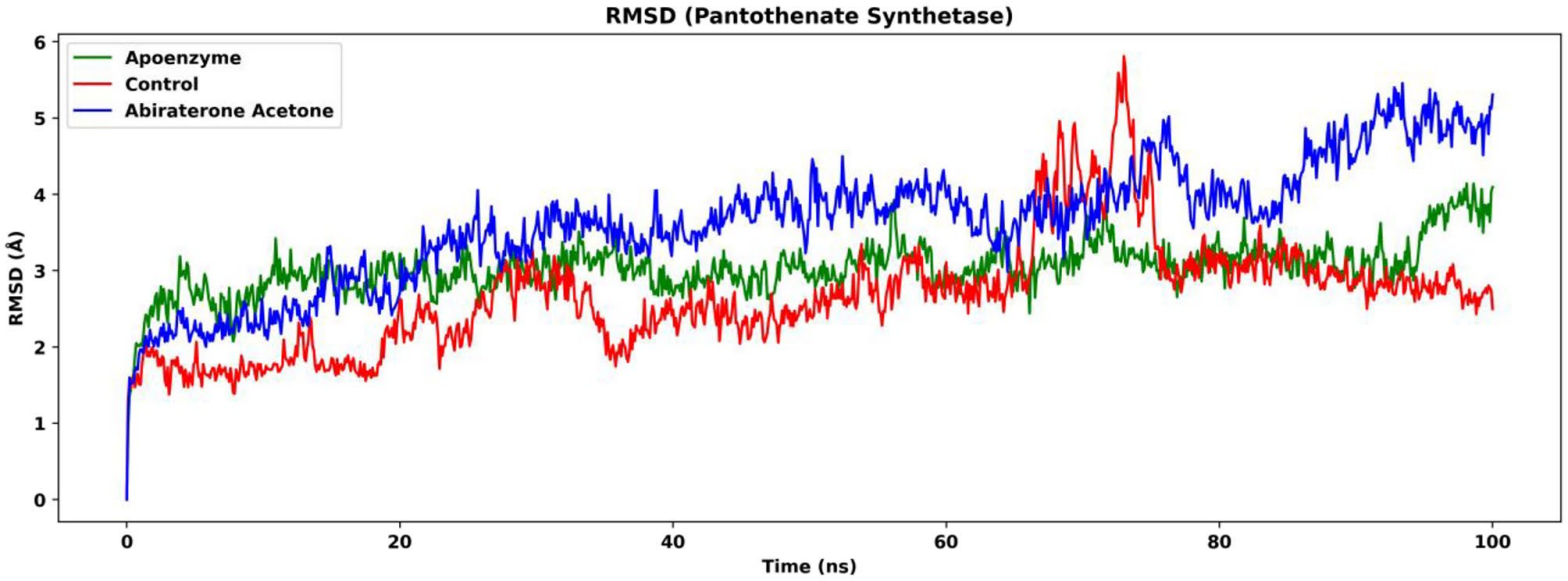
Root mean square deviation (RMSD) of pantothenate synthetase in three systems, namely Apoenzyme (apoenzyme system), Control (pantothenate synthetase-C_15_H_18_N_4_O_6_S complex) and Abiraterone Acetate (pantothenate synthetase-abiraterone acetate complex). Only the C-alpha atoms are considered.

MD simulation is performed for pantothenate synthetase for three systems, the apoenzyme, the control system and the pantothenate synthetase-abiraterone acetate complex. The control system is the pantothenate synthetase-(2 s)-2-Amino-4-({4-[(5-methyl-1,2-oxazol-3-yl)sulfamoyl]phenyl}carbamoyl)butanoic acid (pantothenate synthetase-C_15_H_18_N_4_O_6_S) complex, where C_15_H_18_N_4_O_6_S is an effective drug targeting pantothenate synthetase according to previous research^80^. The RMSD plot, in which only C-alpha atoms are considered, shows that all three systems attain equilibrium after 80 ns (Fig. 13). The protein-abiraterone acetate system has the highest value, followed by the apoenzyme and control systems (after 80 ns, protein-abiraterone acetate: mean: 4.57 Å, SD: 0.495 Å; apoenzyme: mean: 3.32 Å, SD: 0.338 Å; control: mean: 2.91 Å, SD: 0.206 Å), which means that the protein-abiraterone acetate complex fluctuates the most, and the binding the abiraterone acetate might destabilize the enzyme. The difference in RMSD between the apoenzyme and the control systems is small and not very significant. This indicates that the binding of abiraterone acetate decreases the degree of fluctuation of the protein, while that of C_15_H_18_N_4_O_6_S does not affect the protein significantly in terms of RMSD, which might suggest that abiraterone acetate is a better drug when compared to C_15_H_18_N_4_O_6_S.

**Figure 14:**
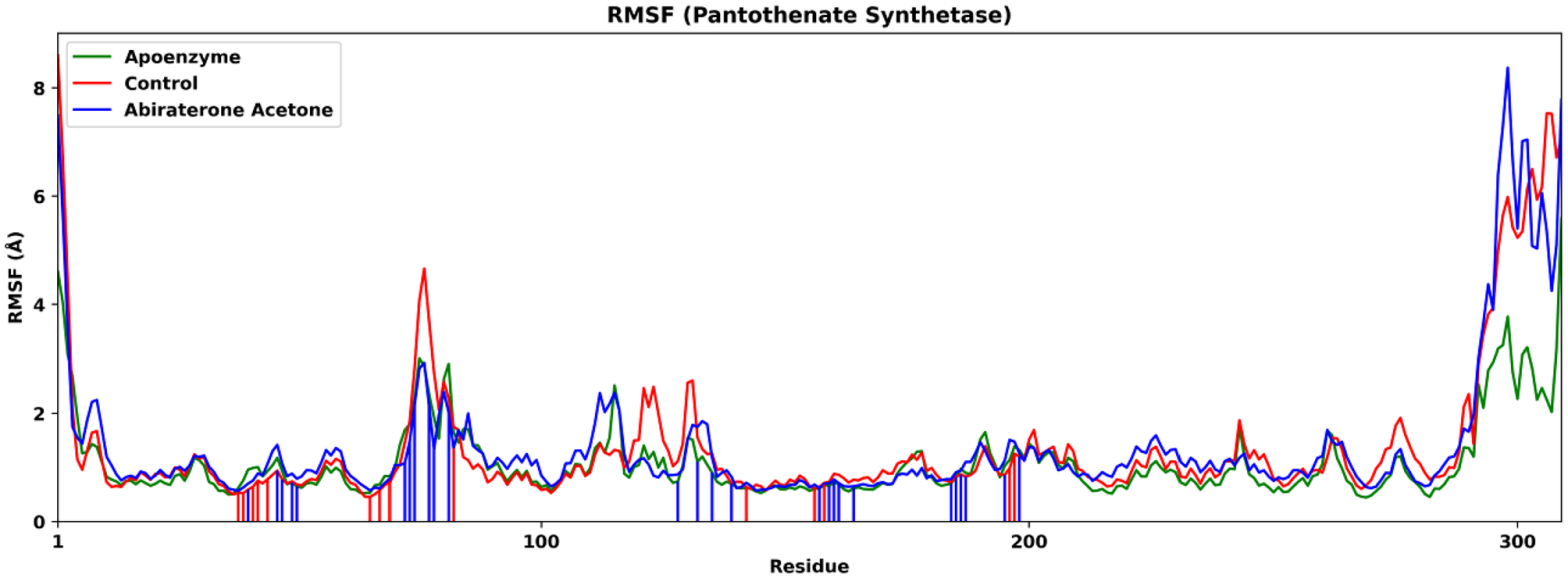
Root mean square fluctuation (RMSF) of pantothenate synthetase in three systems, namely Apoenzyme (apoenzyme system), Control (pantothenate synthetase-C_15_H_18_N_4_O_6_S complex) and Abiraterone Acetate (pantothenate synthetase-abiraterone acetate complex). Only the C-alpha atoms are considered. The red and blue vertical lines represent the residues that interact with the ligand in the Control and Abiraterone Acetate systems, respectively. Only the C-alpha atoms are considered.

The degree of fluctuation of various residues in the three systems is shown in the RMSF plot (Fig. 14). The overall RMSF is highest for the protein-abiraterone acetate system. The aforementioned higher fluctuation protein-abiraterone acetate system is mainly contributed by the residues near the C-terminal. For the control system, the C_15_H_18_N_4_O_6_S ligand interacts with pantothenate synthetase mainly via Pro38–Leu50, Ser65–Tyr82, Arg132–Val142, Phe156–Gln164, Thr186–Val187 and Met195–Arg198. For the protein-abiraterone acetate complex system, the abiraterone acetate ligand interacts with the enzyme mainly via Met40–Leu50, Gln72–Ala81, Glu128–Val139, Phe157–Gln164, Val184–Val187 and Met195–Arg198. It should be noted that the regions near the residues that have interactions with the drugs do not have a huge change in fluctuation.

**Figure 15:**
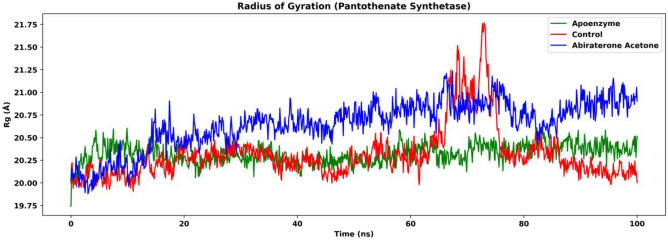
The radius of gyration (Rg) of pantothenate synthetase in three systems, namely Apoenzyme (apoenzyme system), Control (pantothenate synthetase-C_15_H_18_N_4_O_6_S complex) and Abiraterone Acetate (pantothenate synthetase-abiraterone acetate complex). Only the protein backbone is considered.

The Rg, which is an indicator of compactness, of the three systems are also analyzed. Figure 15 shows that the apoenzyme has the lowest Rg among the three systems, which suggests that it is the most compact system. The control system has similar Rg values when compared to the apoenzyme one, while the protein-abiraterone acetone system has overall higher Rg values (after 80 ns, apoenzyme: mean: 20.4 Å, SD: 0.0821 Å; control: mean: 20.2 Å, SD: 0.128 Å; protein-abiraterone acetone: mean: 20.8 Å, SD: 0.148 Å). This indicates that the binding of abiraterone acetone makes the pantothenate synthetase more expanded, which could be due to the hydrogen bonds formed between the protein and the ligand. The lower compactness might destabilize the enzyme. It is noteworthy that the binding of C_15_H_18_N_4_O_6_S does not lower the compactness much because of the small difference in Rg values between the protein-C_15_H_18_N_4_O_6_S complex and the apoenzyme. This might again suggest that abiraterone acetone is a more effective drug, which is supported by the RMSD results.

**Figure 16:**
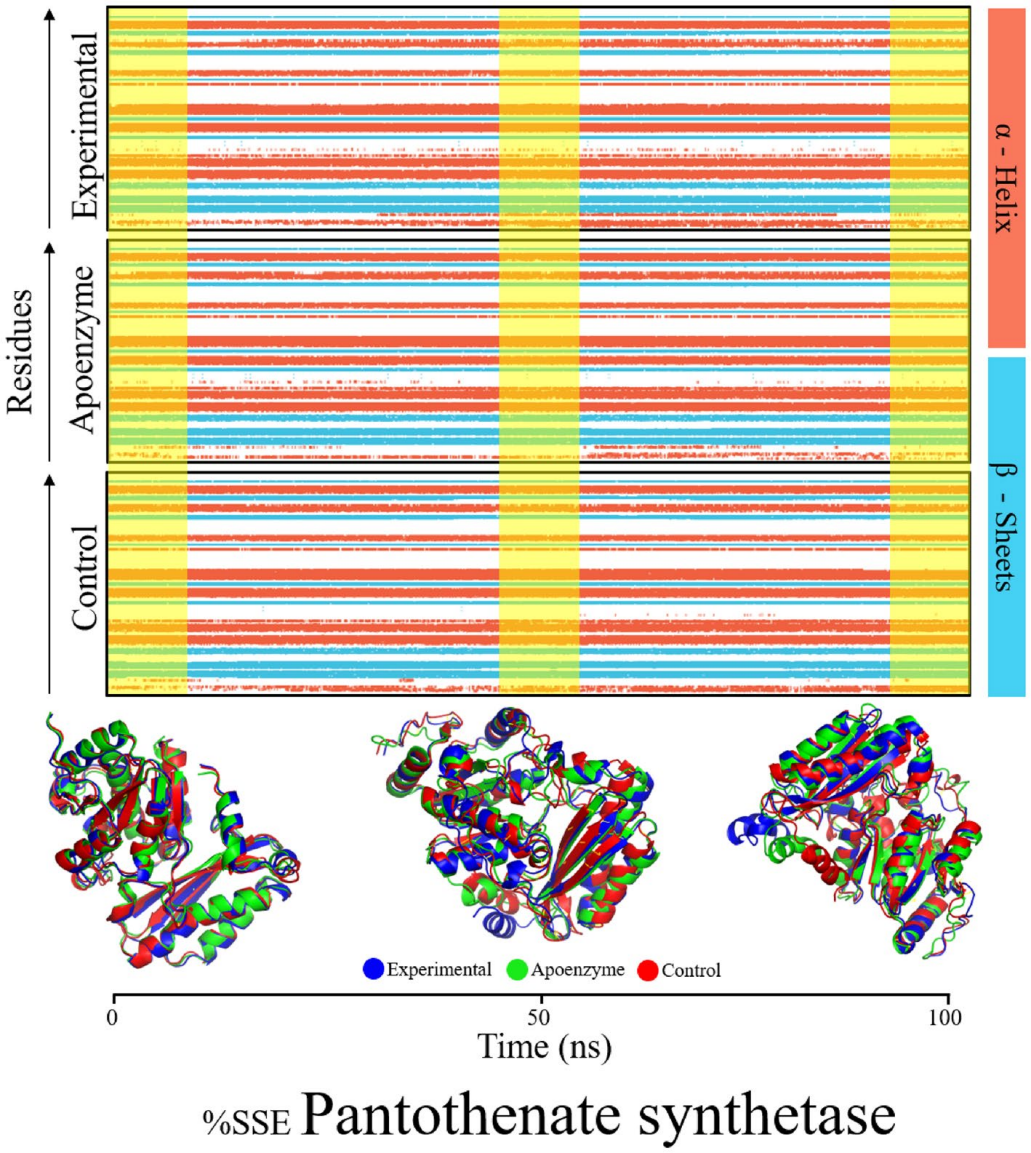
%age secondary structure timeline of of pantothenate synthetase in three systems, namely Apoenzyme (apoenzyme system), Control (pantothenate synthetase-C_15_H_18_N_4_O_6_S complex) and Abiraterone Acetate (pantothenate synthetase-abiraterone acetate complex).

The % secondary structure plot shows the distribution of secondary structures inside the pantothenate synthetase simulations in the apoenzyme, control and protein-abiraterone acetate systems (Fig. 16). In the pantothenate synthetase-abiraterone acetate simulation, the protein has 31.82% alpha-helix, 19.12% beta-strand, and a total secondary shape element (SSE) content of 50.94%. Similarly, the apoenzyme simulation demonstrates 31.08% helix, 18.38% strand, and a total SSE of 49.46%, whilst the control simulation indicates 31.88% helix, 18.70% strand, and a complete SSE of 50.57%. The aligned timeline plot depicts the development of these simulations through the simulation of 100 ns. Additionally, the frames of the three systems were extracted at 1, 50, and 100 ns and then superimposed on each other. There was no major difference observed which reinforces the stability of protein structures.

**Figure 17:**
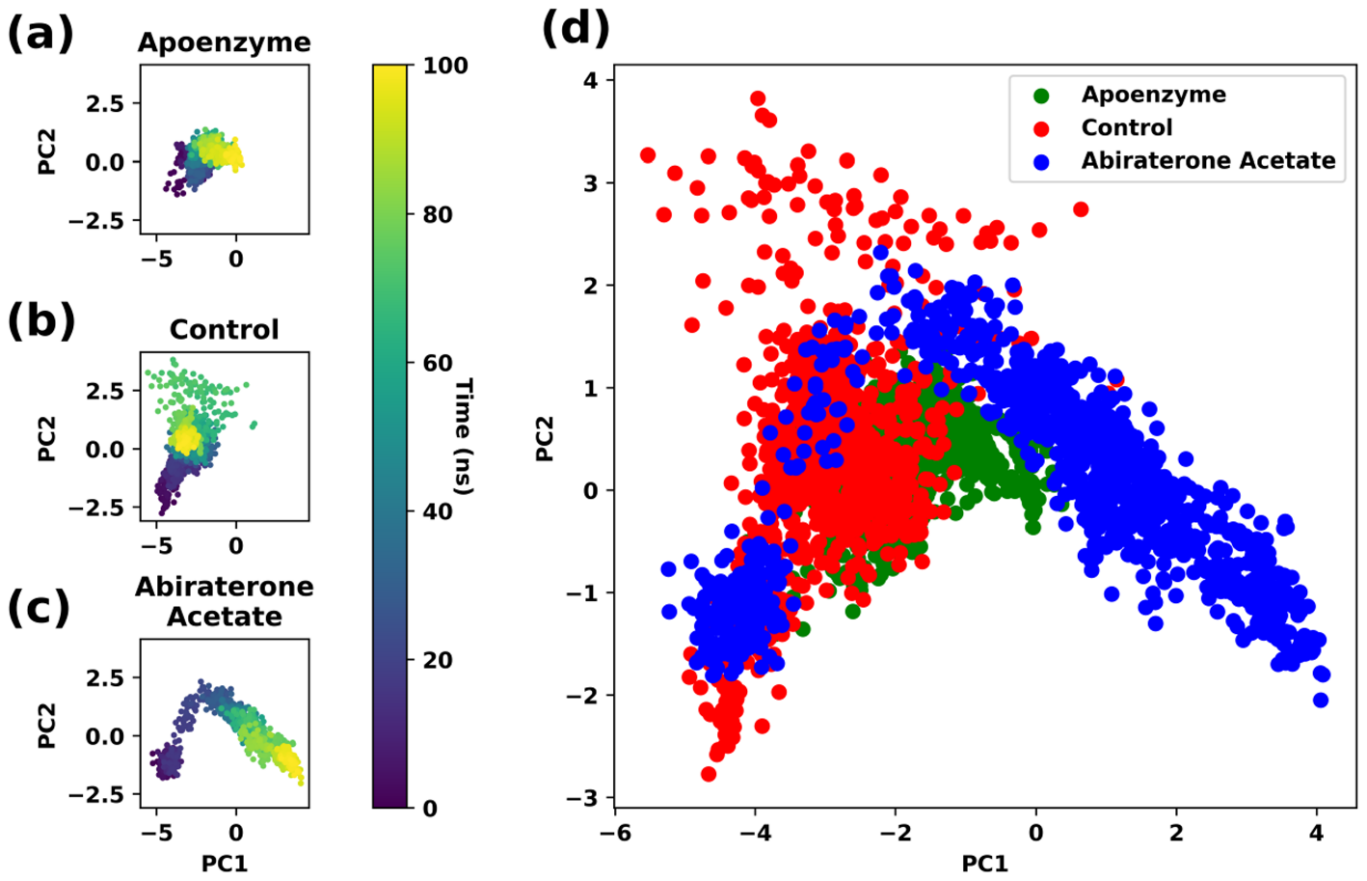
Principal component analysis (PCA) of pantothenate synthetase in three systems, namely Apoenzyme (apoenzyme system, **a**), Control (pantothenate synthetase-C_15_H_18_N_4_O_6_S complex, **b**) and Abiraterone Acetate (pantothenate synthetase-abiraterone acetate complex, **c**), and (**d**) the overlaid PCA plot of three systems. Only the C-alpha atoms are considered.

PCA is conducted for analysis of the trajectories. The C-alpha coordinates of all protein systems are projected to the principal components generated with the pantothenate synthetase-abiraterone acetate system. Substantial differences in their trajectories and conformational changes for the three systems are observed in the PCA plots (Fig. 17), and there are some degrees of overlapping among them. The control system overlaps with the apoenzyme one considerably although there are some points of the control system that do not lie in the apoenzyme regions. In contrast, the overlapping between the pantothenate synthetase-abiraterone acetate system, which occupies roughly two regions, and the apoenzyme one is extremely small, or they almost do not overlap. This might indicate that the binding of abiraterone acetate can better distort the structure of the protein so that it is less similar to its native structure when compared to C_15_H_18_N_4_O_6_S, which in turn suggests that abiraterone acetate is a better inhibitor of pantothenate synthetase. This agrees with the results of the RMSD and Rg analyses. It should be noted that some degree of overlap between the control and protein-abiraterone acetate regions exists, indicating that both drugs inhibit the protein by guiding it to a similar distorted conformation.

**Figure 18:**
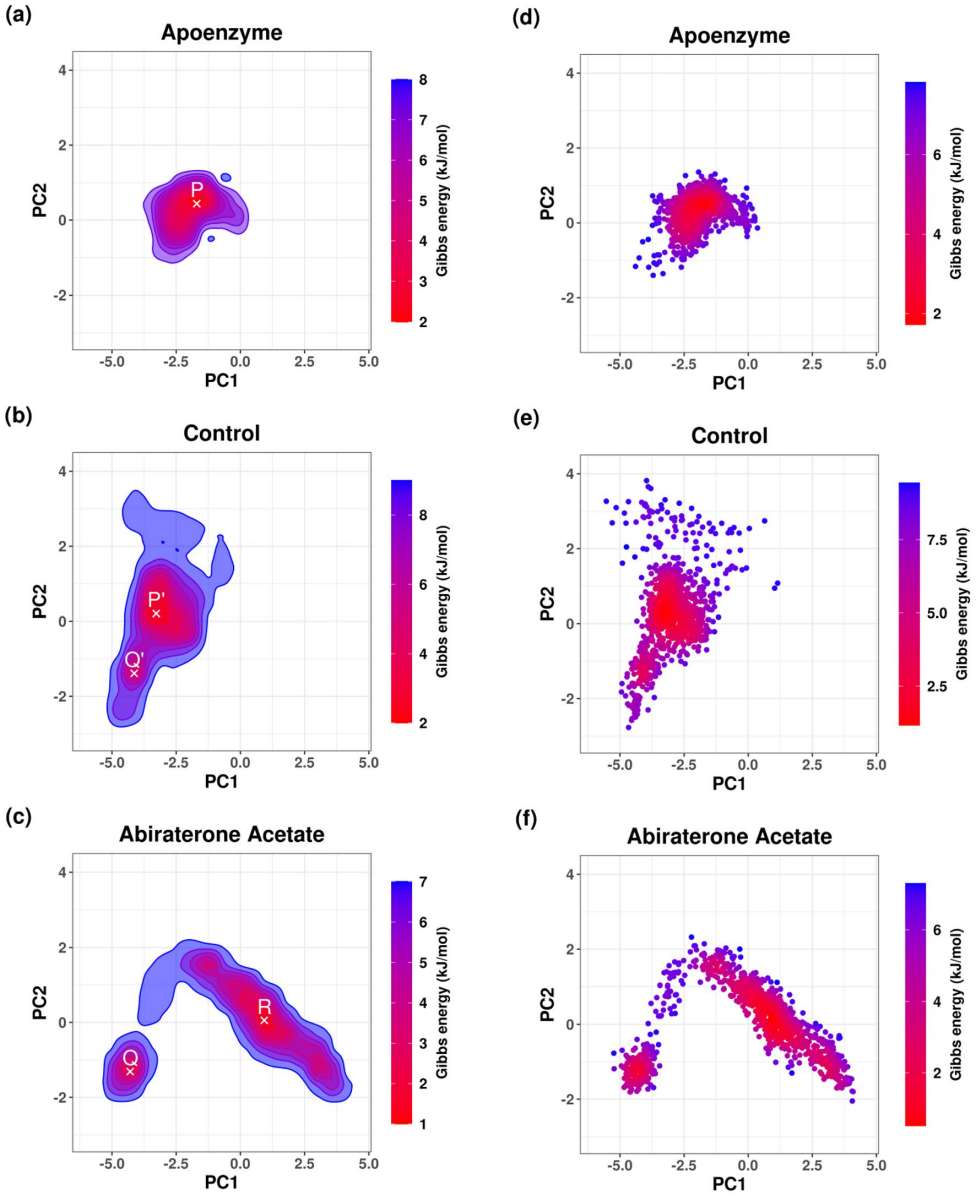

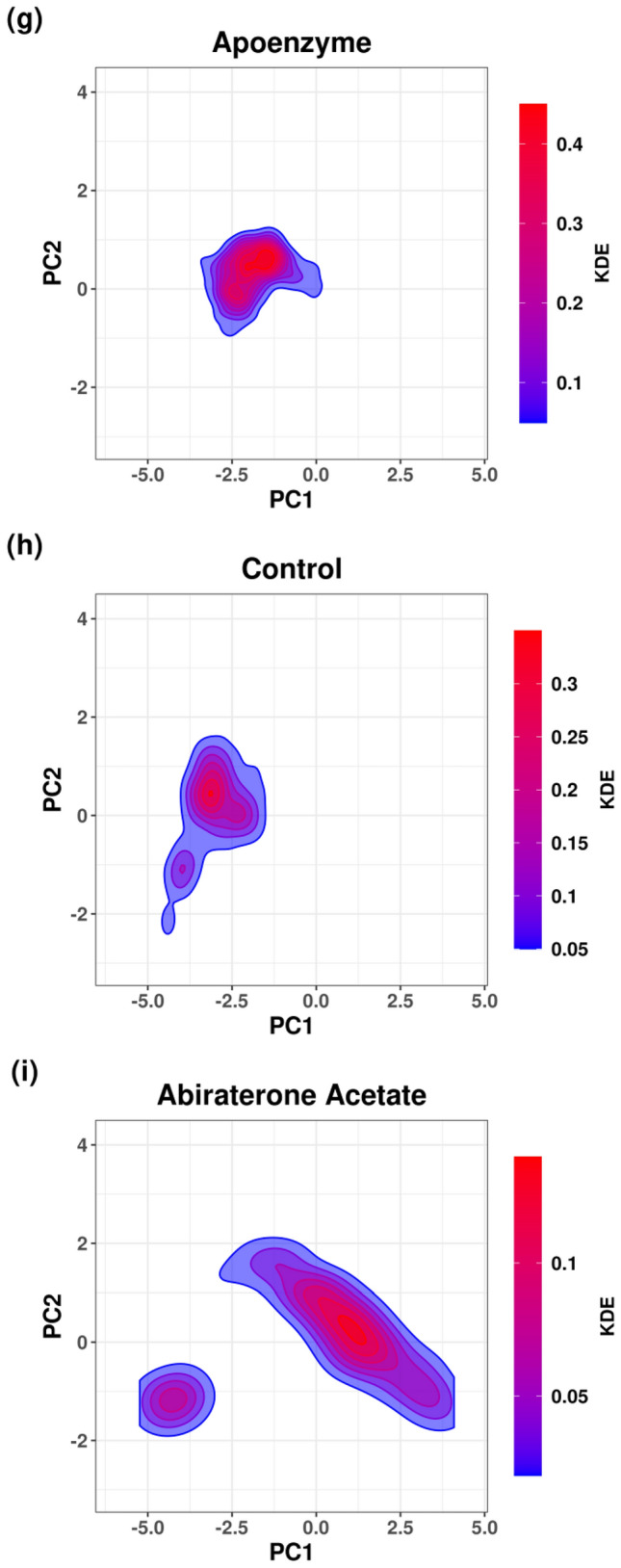
Free energy landscape (FEL) of pantothenate synthetase in three systems, namely Apoenzyme (apoenzyme system, **a**), Control (pantothenate synthetase-C_15_H_18_N_4_O_6_S complex, **b**) and Abiraterone Acetate (pantothenate synthetase-abiraterone acetate complex, **c**). PCA results colored by Gibbs free energy of Apoenzyme (**d**), Control (**e**) and Abiraterone Acetate (**f**). Kernel density estimation (KDE) plots of PCA results of Apoenzyme (**g**), Control (**h**) and Abiraterone Acetate (**i**).

Using the result of PCA, FEL is plotted for the analysis of the metastable conformations, and it is verified by the KDE plots (Fig. 18). In the apoenzyme system, only one energy minimum or metastable conformer (conformer P) is observed. In the control system, two metastable conformers, one with lower energy (conformer P’) and another with higher energy (conformer Q’), exist. The protein-abiraterone acetate system has two metastable conformers (conformers Q and R). In terms of the positions of the conformers, P is similar to P’ and Q is similar to Q’. Due to the similarity between P’ and P, the conformation adopted by the control system is close to that adopted by the apoenzyme because P’ has lower energy than Q’, which means that the control system stays at P’ for a longer period. The conformations adopted by the protein-abiraterone acetate system are very different when compared to the apoenzyme because Q and R do not lie close to P. These results indicate that the binding to ligands can change the conformation of the protein. When C_15_H_18_N_4_O_6_S is bound, the major conformation changes from P to P’, while it changes from P to Q and R when abiraterone acetate is bound, from which we can also deduce that abiraterone acetate can make a more significant modification to the protein conformations. We should also note the similarity between Q in the protein-abiraterone acetate system and Q’ in the control system, which indicates that both drugs change the conformation towards the Q or Q’ direction, but C_15_H_18_N_4_O_6_S does so less effectively because the energy of Q’ is high when compared to the dominant conformer P’ of the control system.

**Figure 19:**
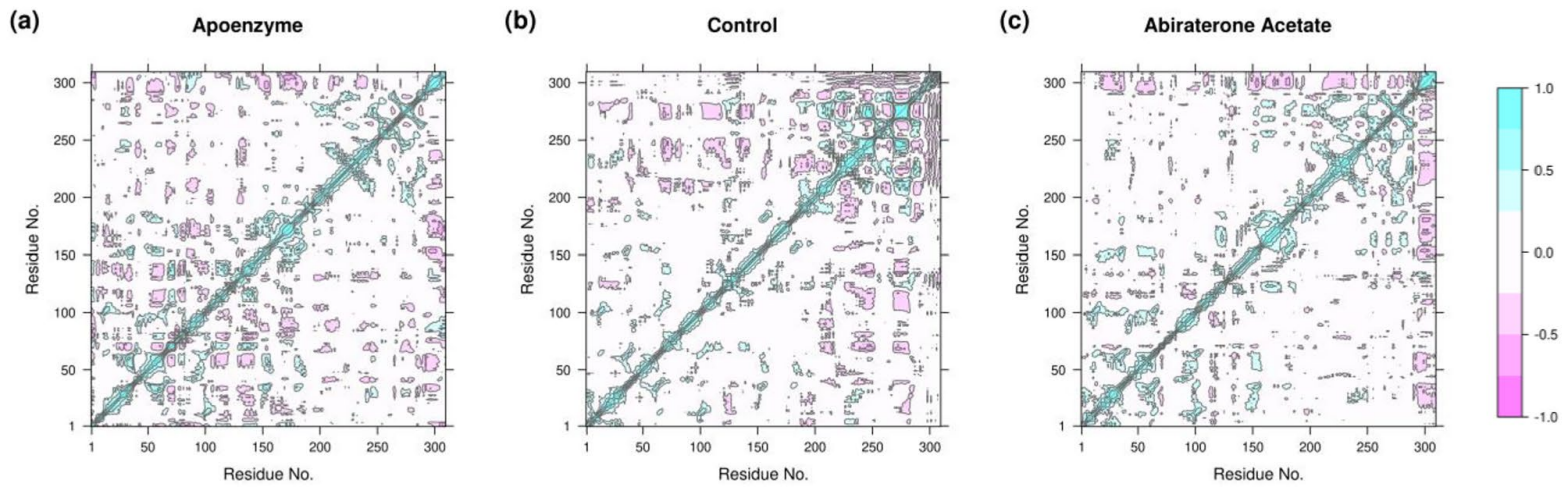
Dynamic cross-correlation matrix (DCCM) of pantothenate synthetase in three systems, namely Apoenzyme (apoenzyme system, **a**), Control (pantothenate synthetase-C_15_H_18_N_4_O_6_S complex, **b**) and Abiraterone Acetate (pantothenate synthetase-abiraterone acetate complex, **c**).

DCCM is used to analyze the correlation of motions of residues in the three protein systems. The RMSD results show that the three systems attain equilibrium after 80 ns, so we extracted those frames for DCCM analysis. The three systems show very different patterns of DCCM (Fig. 19). For the apoenzyme, the major correlation comes from the residues 1–200. The binding of C_15_H_18_N_4_O_6_S changes the correlation patterns such that the contribution is mainly from residues 200–300. The protein-abiraterone acetate system exhibits correlations in motions between residues 295–309 and all other parts of the protein. Also, the reduction of correlations caused by binding of drugs is higher for abiraterone acetate than C_15_H_18_N_4_O_6_S, this might further support the previous deduction that abiraterone acetate is a more effective drug.

**Figure 20:**
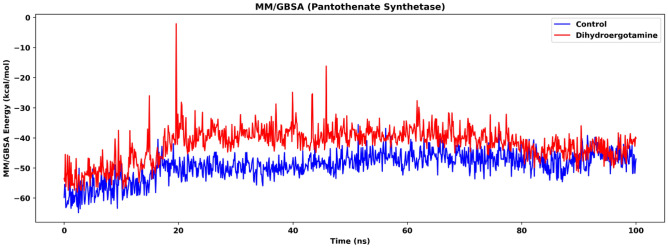
MM/GBSA energy of pantothenate synthetase in the two ligand-bound systems, namely Control (pantothenate synthetase-C_15_H_18_N_4_O_6_S complex, **b**) and Abiraterone Acetate (pantothenate synthetase-abiraterone acetate complex, **c**).

MM/GBSA is employed to evaluate the binding energy of pantothenate synthetase and the two drugs, namely C_15_H_18_N_4_O_6_S and abiraterone acetate. The MM/GBSA results show overall higher energy of the binding of abiraterone acetate (Fig. 20). Despite this, after the system has equilibrated after 80 ns according to the RMSD results, there is, in fact, no significant difference in binding energy between the two systems (after 80 ns, pantothenate synthetase-C_15_H_18_N_4_O_6_S complex: mean: − 47.2 kcal/mol, SD: 3.32 kcal/mol; pantothenate synthetase-abiraterone acetate complex: mean: − 44.0 kcal/mol, SD: 2.78 kcal/mol). While the difference in binding energy is small, the binding of abiraterone acetate can induce a higher degree of conformational change of the protein according to the results of PCA and FEL. This shows that the binding energy is not disadvantageous for the drug effectiveness of abiraterone acetate.

## Conclusion

Exploring new ways to fight TB is essential to reducing the need for extensive treatment and drug resistance. The study identifies the enzymes isocitrate lyase and pantothenate synthetase as promising targets for treating *M. tuberculosis* strains, whether drug-sensitive or drug-resistant. In addition, this research has uncovered the best ligands (dihydroergotamine, abiraterone acetate) with high binding affinity for these drug targets, providing crucial direction for upcoming *M. tuberculosis* drug development efforts. These findings suggest efficient TB therapeutics that may even replace currently available medications. However, it is necessary to emphasize that experimental validation is essential to confirm these results’ authenticity.

### Gaps and limitations

While this study identified promising drug candidates, further testing is necessary. In vitro and in vivo experiments will be crucial to confirm the efficacy and safety of these drugs for treating Mycobacterium tuberculosis infection.

### Supplementary Information

Supplementary Information 1.

Supplementary Information 2.

Supplementary Table S1.

Supplementary Table S2.

## Data Availability

The datasets used and analyzed during the current study have already been reported here. Any further information needed will be made available by the correspondence author.
