# Supplementary material for: Exploring optimal drug targets through subtractive proteomics analysis and pangenomic insights for tailored drug design in tuberculosis
Source: Sci Rep. 2024 May 13;14:10904. doi: 10.1038/s41598-024-61752-6 (PMC11091173; doi:10.1038/s41598-024-61752-6)
Supplement: Supplementary file 1 — Supplementary Information 1. [file 41598_2024_61752_MOESM1_ESM.docx]

**File S3. Projected virulence-associated proteins from the non-homologous essential core proteins given in FASTA format below:**

>CORE_REP|Org167_Gene1759#

MQPTGIAIIGLACRFPTVVSPGDLWDLLRDGREAAGSIDNVADFDADFFNLSPREASAMDPRQRLALELTWELLEDAFVVPETLRGQPIAVYLGAMNDDYAVLTLAADRVDHHAFAGTSRAIIANRVSFAFGLRGPSVTIDSGQSSSLVAVHLACESVRTGEAPLAIAGGVHLNLARETAMLEQEFGAVSPSGHTYAFDERADGYVPGDGGGLVLLKPVQAALDDGDRIHAIIRGSAVGNAGHSATGLTVPSVAGQVDVIRRAMSGAGVDCHQVHYVEAHGTGTKIGDPIEARALGEIFAARQRRPVSVGSVKTNIGHTGGAAGIAGLLKAVLAIENAVIPPSLNYVGAAIDLDSLGLRVDTALTPWPVADEPRRAGVSSFGMGGTNAHVILEQGPTQSPEIVESVAAAGSNAPVAVPWVLAARSPQALTNQAGRLLAHLTADDGLTALDVGWSLVSTRSVFDHRAVVVGADRGRLMAGLAGLAAGEPGAGVVVGRARSVGKTVFVFPGQGSQWLGMGRQLYGRYSVFARAFDEVVAVLDGQLRLSVRQVMWGADAGLLESTEFAQPALFVVQVALAALLQDWGVLPDLVMGHSVGEIAAAYVAGALSLVDAARVVAARGRLMQALPAGGVMVAVAASEDEVAPLLTEGVCIAAVNAPESVVISGEQAAVGVVVDRLVGLGRRVRRLAVSHAFHSVLMDPMVEEFSKVLADVCVRAPRIGLVSNVTGQLAGAGYGSPAYWVEHVRKPVRFFDGVGLAESLGARVFVEVGPGAGLEASVALLARDRPEVESVLAGVGRLFAEGVAVDWSSVFAGLGGRRVELPTYGFARQRFWLGDNGELSVDQTGKDAGAIARLQSLAPPELQRQLVELVCFHAAIVLGRKSSHDIDPECAFQDLGFDSMSGVELRNRLQMAIGLPGLSLPRTLIFDYPTASALAECLGQLLGGQHESSDDESIWQLLKNIPIHQLRRTGLLDKLLLLAGQPEESLAGRTVSDEVIDSLSPEALIGLALDEDENDIR

>CORE_REP|Org372_Gene2930#

MAPKQLPDGRVAVLLSAHAEELIGPDARAIADYLERFPATTVTEVARQLRKTRRVRRHRAVLRAADRLELAEGLRALAAGREHPLIARSSLGSAPRQAFVFPGQGGHWPGMGAVAYRELPTYRTATDTCAAAFAAAGVDSPLPYLIAPPGTDERQAFCEIEIEGAQFVHAVALAEVWRSCGVLPDLTVGHSLGEVAAAYLAGSITLSDAVAVVAARANVVGRLPGRYAVAALGIGEQDASALIATTGGWLELSVVNASSTVAVSGERQAVAAIVDTVRSSGHFARGITVGFPVHTSVLESLRDELCEQLPDSEFMEAPVQFIGGTTGDVVAPGTTFGDYWYANLRHTVRFDRAVESAIRCGARAFIEISAHPALLFAIGQNCEGAANLPDGPAVLVGSARRGERFVDALSANIVSAAVADPGYPWGDLGGDPLDGDVDLSGFPNAPMRAVPMWAHPEPLPPVSGLTIAVERWERMVPSTPVAGRHRHLAVLDLGAHRALAQTLCAAIDSHPDTELSAARDAELILVIAPDFEHTDAVRAAGALADLVGAGLLDYPMHIGARCQSVCLVTVGAEQVDAADAVPSAGQAALAAMHRSIGFEHPEQTFSHLDLPSWDLDPVLGVSVITAVLRGFGETALRGSVNGYTLFERTLADAPAVPNWSLDSGVLDDVVVTGGAGAIGMHYARYLAEHGARRIVLLSRRAADQATVAMLRKQHGTVIVSPPCDITDPTQLSAIAAEYGGVGASLIVHAAGSVISGTAPGVTSAAVVDNFAAKVLGLAQMIELWPLRPDVRTLLCSSVMGVWGGHGVVAYSAANRLLDVMAAQLRAQGRHCVAVKWGLWQAPKAGEPARGIADAVTIARVERSGLRQMAPQQAIEASLHEFTVDPLVFAADAARLQMLLDSRQFERYEGPTDPNLTIVDAVRTQLAAVLGIPQAGEVNLQESLFDLGVDSMLALDLRNRLKRSIGATVSLATLMGDITGDGLVAKLEDADERSHTAQKVDISRD

>CORE_REP|Org163_Gene3150#

MSGTPDDGDIGLIIAVKRLAAAKTRLAPVFSAQTRENVVLAMLVDTLTAAAGVGSLRSITVITPDEAAAAAAAGLGADVLADPTPEDDPDPLNTAITAAERVVAEGASNIVVLQGDLPALQTQELAEAISAARHHRRSFVADRLGTGTAVLCAFGTALHPRFGPDSSARHRRSGAVELTGAWPGLRCDVDTPADLTAARQLGGRARDRASGRTSLTGTGQRRRGIQGGERQTNGERMPAECWQPHPMMSNDRKVTEIENSPVTEVRPEEHAWYPDDSALAAPPAATPAAISDQLPSDRYLNRELSWLDFNARVLALAADKSMPLLERAKFLAIFASNLDEFYMVRVAGLKRRDEMGLSVRSADGLTPREQLGRIGEQTQQLASRHARVFLDSVLPALGEEGIYIVTWADLDQAERDRLSTYFNEQVFPVLTPLAVDPAHPFPFVSGLSLNLAVTVRQPEDGTQHFARVKVPDNVDRFVELAAREASEEAAGTEGRTALRFLPMEELIAAFLPVLFPGMEIVEHHAFRITRNADFEVEEDRDEDLLQALERELARRRFGSPVRLEIADDMTESMLELLLRELDVHPGDVIEVPGLLDLSSLWQIYAVDRPTLKDRTFVPATHPAFAERETPKSIFATLREGDVLVHHPYDSFSTSVQRFIEQAAADPNVLAIKQTLYRTSGDSPIVRALIDAAEAGKQVVALVEIKARFDEQANIAWARALEQAGVHVAYGLVGLKTHCKTALVVRREGPTIRRYCHVGTGNYNSKTARLYEDVGLLTAAPDIGADLTDLFNSLTGYSRKLSYRNLLVAPHGIRAGIIDRVEREVAAHRAEGAHNGKGRIRLKMNALVDEQVIDALYRASRAGVRIEVVVRGICALRPGAQGISENIIVRSILGRFLEHSRILHFRAIDEFWIGSADMMHRNLDRRVEVMAQVKNPRLTAQLDELFESALDPCTRCWELGPDGQWTASPQEGHSVRDHQESLMERHRSP

>CORE_REP|Org27_Gene1697#

MSFSERDSVADRLIVKGAREHNLRSVDLDLPRDALIVFTGLSGSGKSSLAFDTIFAEGQRRYVESLSAYARQFLGQMDKPDVDFIEGLSPAVSIDQKSTNRNPRSTVGTITEVYDYLRLLYARAGTPHCPTCGERVARQTPQQIVDQVLAMPEGTRFLVLAPVVRTRKGEFADLFDKLNAQGYSRVRVDGVVHPLTDPPKLKKQEKHDIEVVVDRLTVKAAAKRRLTDSVETALNLADGIVVLEFVDHELGAPHREQRFSEKLACPNGHALAVDDLEPRSFSFNSPYGACPECSGLGIRKEVDPELVVPDPDRTLAQGAVAPWSNGHTAEYFTRMMAGLGEALGFDVDTPWRKLPAKARKAILEGADEQVHVRYRNRYGRTRSYYADFEGVLAFLQRKMSQTESEQMKERYEGFMRDVPCPVCAGTRLKPEILAVTLAGESKGEHGAKSIAEVCELSIADCADFLNALTLGPREQAIAGQVLKEIRSRLGFLLDVGLEYLSLSRAAATLSGGEAQRIRLATQIGSGLVGVLYVLDEPSIGLHQRDNRRLIETLTRLRDLGNTLIVVEHDEDTIEHADWIVDIGPGAGEHGGRIVHSGPYDELLRNKDSITGAYLSGRESIEIPAIRRSVDPRRQLTVVGAREHNLRGIDVSFPLGVLTSVTGVSGSGKSTLVNDILAAVLANRLNGARQVPGRHTRVTGLDYLDKLVRVDQSPIGRTPRSNPATYTGVFDKIRTLFAATTEAKVRGYQPGRFSFNVKGGRCEACTGDGTIKIEMNFLPDVYVPCEVCQGARYNRETLEVHYKGKTVSEVLDMSIEEAAEFFEPIAGVHRYLRTLVDVGLGYVRLGQPAPTLSGGEAQRVKLASELQKRSTGRTVYILDEPTTGLHFDDIRKLLNVINGLVDKGNTVIVIEHNLDVIKTSDWIIDLGPEGGAGGGTVVAQGTPEDVAAVPASYTGKFLAEVVGGGASAATSRSNRRRNVSA

>CORE_REP|Org163_Gene1928#

MTSSRAVNVHGCPRIAACRCTDTHPRGRPAFAYRWFVPKTTRAQPGRLSSRFWRLLGASTEKNRSRSLADVTASAEYDKEAADLSDEKLRKAAGLLNLDDLAESADIPQFLAIAREAAERRTGLRPFDVQLLGALRMLAGDVIEMATGEGKTLAGAIAAAGYALAGRHVHVVTINDYLARRDAEWMGPLLDAMGLTVGWITADSTPDERRTAYDRDVTYASVNEIGFDVLRDQLVTDVNDLVSPNPDVALIDEADSVLVDEALVPLVLAGTTHRETPRLEIIRLVAELVGDKDADEYFATDSDNRNVHLTEHGARKVEKALGGIDLYSEEHVGTTLTEVNVALHAHVLLQRDVHYIVRDDAVHLINASRGRIAQLQRWPDGLQAAVEAKEGIETTETGEVLDTITVQALINRYATMCGMTGTALAAGEQLRQFYQLGVSPIPPNKPNIREDEADRVYITTAAKNDGIVEHITEVHQRGQPVLVGTRDVAESEELHERLVRRGVPAVVLNAKNDAEEARVIAEAGKYGAVTVSTQMAGRGTDIRLGGSDEADHDRVAELGGLHVVGTGRHHTERLDNQLRGRAGRQGDPGSSVFFSSWEDDVVAANLDHNKLPMATDENGRIVSPRTGSLLDHAQRVAEGRLLDVHANTWRYNQLIAQQRAIIVERRNTLLRTVTAREELAELAPKRYEELSDKVSEERLETICRQIMLYHLDRGWADHLAYLADIRESIHLRALGRQNPLDEFHRMAVDAFASLAADAIEAAQQTFETANVLDHEPGLDLSKLARPTSTWTYMVNDNPLSDDTLSALSLPGVFR

>CORE_REP|Org170_Gene40#

MDTDNDRPTLARVYRSLRDICPDSWNLPGGRMPTGLGYDFLRPVEDSGINDLKHYYFMADLADGQPLGRANLYSVCFDLATTDRKLTPAWRTTIKRWFPGFMTFRFLECGLLTMVSNPLALRSDTDLERVLPVLAGQMDQLAHDDGSDFLMIRDVDPEHYQRYLDILRPLGFRPALGFSRVDTTISWSSVEEALGCLSHKRRLPLKTSLEFRERFGIEVEELDEYAEHAPVLARLWRNVKTEAKDYQREDLNPEFFAACSRHLHGRSRLWLFRYQGTPIAFFLNVWGADENYILLEWGIDRDFEHYRKANLYRAALMLSLKDAISRDKRRMEMGITNYFTKLRIPGARVIPTIYFLRHSTDPVHTATLARMMMHNIQRPTLPDDMSEEFCRWEERIRLDQDGLPEHDIFRKIDRQHKYTGLKLGGVYGFYPRFTGPQRSTVKAAELGEIVLLGTNSYLGLATHPEVVEASAEATRRYGTGCSGSPLLNGTLDLHVSLEQELACFLGKPAAVLCSTGYQSNLAAISALCESGDMIIQDALNHRSLFDAARLSGADFTLYRHNDMDHLARVLRRTEGRRRIIVVDAVFSMEGTVADLATIAELADRHGCRVYVDESHALGVLGPDGRGASAALGVLARMDVVMGTFSKSFASVGGFIAGDRPVVDYIRHNGSGHVFSASLPPAAAAATHAALRVSRREPDRRARVLAAAEYMATGLARQGYQAEYHGTAIVPVILGNPTVAHAGYLRLMRSGVYVNPVAPPAVPEERSGFRTSYLADHRQSDLDRALHVFAGLAEDLTPQGAAL

>CORE_REP|Org172_Gene2465#

MAEDQLTAQAVAPPTEASAALEPALETPESPVETLKTSISASRRVRARLARRMTAQRSTTNPVLEPLVAVHREIYPKADLSILQRAYEVADQRHASQLRQSGDPYITHPLAVANILAELGMDTTTLVAALLHDTVEDTGYTLEALTEEFGEEVGHLVDGVTKLDRVVLGSAAEGETIRKMITAMARDPRVLVIKVADRLHNMRTMRFLPPEKQARKARETLEVIAPLAHRLGMASVKWELEDLSFAILHPKKYEEIVRLVAGRAPSRDTYLAKVRAEIVNTLTASKIKATVEGRPKHYWSIYQKMIVKGRDFDDIHDLVGVRILCDEIRDCYAAVGVVHSLWQPMAGRFKDYIAQPRYGVYQSLHTTVVGPEGKPLEVQIRTRDMHRTAEYGIAAHWRYKEAKGRNGVLHPHAAAEIDDMAWMRQLLDWQREAADPGEFLESLRYDLAVQEIFVFTPKGDVITLPTGSTPVDFAYAVHTEVGHRCIGARVNGRLVALERKLENGEVVEVFTSKAPNAGPSRDWQQFVVSPRAKTKIRQWFAKERREEALETGKDAMAREVRRGGLPLQRLVNGESMAAVARELHYADVSALYTAIGEGHVSAKHVVQRLLAELGGIDQAEEELAERSTPATMPRRPRSTDDVGVSVPGAPGVLTKLAKCCTPVPGDVIMGFVTRGGGVSVHRTDCTNAASLQQQAERIIEVLWAPSPSSVFLVAIQVEALDRHRLLSDVTRALADEKVNILSASVTTSGDRVAISRFTFEMGDPKHLGHLLNAVRNVEGVYDVYRVTSAA

>CORE_REP|Org172_Gene1756#

MAIAETDTEVHTPFEQDFEKDVAATQRYFDSSRFAGIIRLYTARQVVEQRGTIPVDHIVAREAAGAFYERLRELFAARKSITTFGPYSPGQAVSMKRMGIEAIYLGGWATSAKGSSTEDPGPDLASYPLSQVPDDAAVLVRALLTADRNQHYLRLQMSERQRAATPAYDFRPFIIADADTGHGGDPHVRNLIRRFVEVGVPGYHIEDQRPGTKKCGHQGGKVLVPSDEQIKRLNAARFQLDIMRVPGIIVARTDAEAANLIDSRADERDQPFLLGATKLDVPSYKSCFLAMVRRFYELGVKELNGHLLYALGDSEYAAAGGWLERQGIFGLVSDAVNAWREDGQQSIDGIFDQVESRFVAAWEDDAGLMTYGEAVADVLEFGQSEGEPIGMAPEEWRAFAARASLHAARAKAKELGADPPWDCELAKTPEGYYQIRGGIPYAIAKSLAAAPFADILWMETKTADLADARQFAEAIHAEFPDQMLAYNLSPSFNWDTTGMTDEEMRRFPEELGKMGFVFNFITYGGHQIDGVAAEEFATALRQDGMLALARLQRKMRLVESPYRTPQTLVGGPRSDAALAASSGRTATTKAMGKGSTQHQHLVQTEVPRKLLEEWLAMWSGHYQLKDKLRVQLRPQRAGSEVLELGIHGESDDKLANVIFQPIQDRRGRTILLVRDQNTFGAELRQKRLMTLIHLWLVHRFKAQAVHYVTPTDDNLYQTSKMKSHGIFTEVNQEVGEIIVAEVNHPRIAELLTPDRVALRKLITKEA

>CORE_REP|Org325_Gene1100#

MSFLKTVPEELTAAAAQLGTIGAAMAAQNAAAAAPTTAIAPAALDEVSALQAALFTAYGTFYQQVSAEAQAMHDMFVNTLGISAGTYGVTESLNSSAAASPLSGITGEASAIIQATTGLFPPELSGGIGNILNIGAGNWASATSTLIGLAGGGLLPAEEAAEAASALGGEAALGELGALGAAEAALGEAGIAAGLGSASAIGMLSVPPAWAGQATLVSTTSTLPGAGWTAAXXXXRRPPRGRSSPGCQGWPRPHATAPASVHRATASNPSSCPSQQPSRRQTYGRPRSTNNEIQPNNSTETEYPMDFGALPPEINSARMYAGAGAGPMMAAGAAWNGLAAELGTTAASYESVITRLTTESWMGPASMAMVAAAQPYLAWLTYTAEAAAHAGSQAMASAAAYEAAYAMTVPPEVVAANRALLAALVATNVLGINTPAIMATEALYAEMWAQDALAMYGYAAASGAAGMLQPLSPPSQTTNPGGLAAQSAAVGSAAATAAVNQVSVADLISSLPNAVSGLASPVTSVLDSTGLSGIIADIDALLATPFVANIINSAVNTAAWYVNAAIPTAIFLANALNSGAPVAIAEGAIEAAEGAASAAAAGLADSVTPAGLGASLGEATLVGRLSVPAAWSTAAPATTAGATALEGSGWTVAAEEAGPVTGMMPGMASAAKGTGAYAGPRYGFKPTVMPKQVVV

>CORE_REP|Org389_Gene1228#

MTNPYPNLLSPLDLGFTTLRNRVVMGSMHTGLEDRARHIDRLADYFAERARGGVGLIITGGYAPNRTGWLLPFASELVTSAQARRHRRITRAVHDSGAKILLQILHAGRYAYHPLAVSASPIKAPITPFRPRALSARGVEATIADFARCAQLARDAGYDGVEIMGSEGYLLNQFLAPRTNKRTDSWGGTPANRRRFPVEIIRRSRAAVGSDFIICYRLSMADYVAEGQSWDEIVALATEVEGAGATIINSGFGWHEARVPTIVTSVPGGAFVDISSAVAEHVTIPVVASNRINMPQAAERILAETQVRLISMARPMLSDPDWVLKAQSNRVDEINTCISCNQACLDHAFARKTVSCLLNPRAGRETQLVLSPTRRARSVAVVGAGPAGLATAANAAQRGHRVTLFEANDFIGGQFDMARRIPGKEEFSETIRYFSTILAKHGVEVRLGTRVAAQELTGYDEVVLATGVAPRIPAIPGIDHPMMLTYAEAITGVRPVGRTVAVVGAGGIGFDVTELLVTDSSPTLNLKEWKAEWGVADPREARGALTTPLPAPPAREVYLLQRTKGPQGKRLGKTTGWVHRASLKAKGVHQLSGVNYEQINDDGLHISFGPKRRRPQLLAVDNVVVCAGQEPVRDLESELRRHGINPHIIGGAAVAAELDAKRAIKQGTELAARL

>CORE_REP|Org170_Gene1#

MWNAVVSELNGDPKVDDGPSSDANLSAPLTPQQRAWLNLVQPLTIVEGFALLSVPSSFVQNEIERHLRAPITDALSRRLGHQIQLGVRIAPPATDEADDTTVPPSENPATTSPDTTTDNDEIDDSAAARGDNQHSWPSYFTERPHNTDSATAGVTSLNRRYTFDTFVIGASNRFAHAAALAIAEAPARAYNPLFIWGESGLGKTHLLHAAGNYAQRLFPGMRVKYVSTEEFTNDFINSLRDDRKVAFKRSYRDVDVLLVDDIQFIEGKEGIQEEFFHTFNTLHNANKQIVISSDRPPKQLATLEDRLRTRFEWGLITDVQPPELETRIAILRKKAQMERLAVPDDVLELIASSIERNIRELEGALIRVTAFASLNKTPIDKALAEIVLRDLIADANTMQISAATIMAATAEYFDTTVEELRGPGKTRALAQSRQIAMYLCRELTDLSLPKIGQAFGRDHTTVMYAQRKILSEMAERREVFDHVKELTTHPSALQALARRVLPTTFLKKLLSPRSHQSQRFEPPRHVRRLQFLERMGSCQVVHRGGTRRSCVSGRCGWSQRSAVSTIRKWAAISEVARLLGVGCAETVRKWVRQAQVDAGARPGTTTEESAELKRLRRDNAELRRANAILKTASAFFAAELDRPAR

>CORE_REP|Org106_Gene302#

MAGVGEGDSGGVERDDIGMVAASPVASRVNGKVDADVVGRFATCCRALGIAVYQRKRPPDLAAARSGFAALTRVAHDQCDAWTGLAAAGDQSIGVLEAASRTATTAGVLQRQVELADNALGFLYDTGLYLRFRATGPDDFHLAYAAALASTGGPEEFAKANHVVSGITERRAGWRAARWLAVVINYRAERWSDVVKLLTPMVNDPDLDEAFSHAAKITLGTALARLGMFAPALSYLEEPDGPVAVAAVDGALAKALVLRAHVDEESASEVLQDLYAAHPENEQVEQALSDTSFGIVTTTAGRIEARTDPWDPATEPGAEDFVDPAAHERKAALLHEAELQLAEFIGLDEVKRQVSRLKSSVAMELVRKQRGLTVAQRTHHLVFAGPPGTGKTTIARVVAKIYCGLGLLKRENIREVHRADLIGQHIGETEAKTNAIIDSALDGVLFLDEAYALVATGAKNDFGLVAIDTLLARMENDRDRLVVIIAGYRADLDKFLDTNEGLRSRFTRNIDFPSYTSHELVEIAHKMAEQRDSVFEQSALHDLEALFAKLAAESTPDTNGISRRSLDIAGNGRFVRNIVERSEEEREFRLDHSEHAGSGEFSDEELMTITADDVGRSVEPLLRGLGLSVRA

>CORE_REP|Org101_Gene3957#

MAYHNPFIVNGKIRFPANTNLVRHVEKWAKVRGDKLAYRFLDFSTERDGVARDILWSDFSARNRAVGARLQQVTQPGDRVAILCPQNLDYLISFFGALYSGRIAVPLFDPAEPGHVGRLHAVLDDCAPSTILTTTDSAEGVRKFIRARSAKERPRVIAVDAVPTEVAATWQQPEANEETVAYLQYTSGSTRIPSGVQITHLNLPTNVVQVLNALEGQEGDRGVSWLPFFHDMGLITVLLASVLGHSFTFMTPAAFVRRPGRWIRELARKPGETGGTFSAAPNFAFEHAAVRGVPRDDEPPLDLSNVKGILNGSEPVSPASMRKFFEAFAPYGLKQTAVKPSYGLAEATLFVSTTPMDEVPTVIHVDRDELNNQRFVEVAADAPNAVAQVSAGKVGVSEWAVIVDADTASELPDGQIGEIWLHGNNLGTGYWGKEEESAQTFKNILKSRISESRAEGAPDDALWVRTGDYGTYFKDHLYIAGRIKDLVIIDGRNHYPQDLECTAQESTKALRVGYAAAFSVPANQLPQTVFDDSHAGLKFDPEDTSEQLVIVGERAAGTHKLDHQPIVDDIRAAIAVGHGVTVRDVLLVSAGTIPRTSSGKIGRRACRAAYLDGSLRSGVGSPTVFATSD

>CORE_REP|Org296_Gene3049#

MASRQTPAELARCDLAKTAEREHTPTATATTPSVAGNVMPMSVRSLPAALRACARLQPHDPAFTFMDYEQDWDGVAITLTWSQLYRRTLNVAQELSRCGSTGDRVVISAPQGLEYVVAFLGALQAGRIAVPLSVPQGGVTDERSDSVLSDSSPVAILTTSSAVDDVVQHVARRPGESPPSIIEVDLLDLDAPNGYTFKEDEYPSTAYLQYTSGSTRTPAGVVMSHQNVRVNFEQLMSGYFADTDGIPPPNSALVSWLPFYHDMGLVIGICAPILGGYPAVLTSPVSFLQRPARWMHLMASDFHAFSAAPNFAFELAARRTTDDDMAGRDLGNILTILSGSERVQAATIKRFADRFARFNLQERVIRPSYGLAEATVYVATSKPGQPPETVDFDTESLSAGHAKPCAGGGATSLISYMLPRSPIVRIVDSDTCIECPDGTVGEIWVHGDNVANGYWQKPDESERTFGGKIVTPSPGTPEGPWLRTGDSGFVTDGKMFIIGRIKDLLIVYGRNHSPDDIEATIQEITRGRCAAISVPGDRSTEKLVAIIELKKRGDSDQDAMARLGAIKREVTSALSSSHGLSVADLVLVAPGSIPITTSGKVRRGACVEQYRQDQFARLDA

>CORE_REP|Org341_Gene1385#

MSPLLEVTDLAVTFRTDGDPVTAVRGISYRVEPGEVVAMVGESGSGKSAAAMAVVGLLPEYAQVRGSVRLQGTELLGLADNAMSRFRGKAIGTVFQDPMSALTPVYTVGDQIAEAIEVHQPRVGKKAARRRAVELLDLVGISQPQRRSRAFPHELSGGERQRVVIAIAIANDPDLLICDEPTTALDVTVQAQILDVLKAARDVTGAGVLIITHDLGVVAEFADRALVMYAGRVVESAGVNDLYRDRRMPYTVGLLGSVPRLDAAQGTRLVPIPGAPXXXXLAGLAPGCPFAPRCPLVIDECLTAEPELLDVATDHRAACIRTELVTGRSAADIYRVKTEARPAALGDASVVVRVRHLVKTYRLAKGVVLRRAIGEVRAVDGISLELRQGRTLGIVGESGSGKSTTLHEILELAAPQSGSIEVLGTDVATLGTAERRSLRRDIQVVFQDPVASLDPRLPVFDLIAEPLQANGFGKNETHARVAELLDIVGLRHGDASRYPAEFSGGQKQRIGIARALALQPKILALDEPVSALDVSIQAGIINLLLDLQEQFGLSYLFVSHDLSVVKHLAHQVAVMLAGTVVEQGDSEEVFGNPKHEYTRRLLGAVPQPDPARRG

>CORE_REP|Org156_Gene3842#

MERFDGLRPARLKVGIISAGRVGTALGVALQRADHVVMACSAISHASRRRAQRRLPDTPVLPPLDVAASAELLLLAVTDSELAGLVSGLAATSAVRPQTIVAHTSGANGIGILAPLAQQGCIPLAIHPAMTFTGSDEDISRLPDTCFGITAADDVGYAIGQSLVLEMGGEPFCVREDARILYHAALAHASNHIVTVLADALEALRAALSGGTARPTNRRRPAGRDRGAHRRAAGQSGAGEHAAAGTGRAHRTGRPRRCGSGRGSSGGPRGRRRSAGPGIPDKRAADRAARTRPRGCRRGFDGMTIPAFHPGELNVYSAPGDVADVSRALRLTGRRVMLVPTMGALHEGHLALVRAAKRVPGSVVVVSIFVNPMQFGAGEDLDAYPRTPDDDLAQLRAEGVEIAFTPTTAAMYPDGLRTTVQPGPLAAELEGGPRPTHFAGVLTVVLKLLQIVRPDRVFFGEKDYQQLVLIRQLVADFNLDVAVVGVPTVREADGLAMSSRNRYLDPAQRAAAVALSAALTAAAHAATAGAQAALDAARAVLDAAPGVAVDYLELRDIGLGPMPLNGSGRLLVAARLGTTRLLDNIAIEIGTFAGTDRPDGYRAILESHWRN

>CORE_REP|Org384_Gene1859#

MTRPQAAAEDARNAMVAGLLASGISVNGLQPSHNPQVAAQMFTTATRLDPKMCDAWLARLLAGDQSIEVLAGAWAAVRTFGWETRRLGVTDLQFRPEVSDGLFLRLAITSVDSLACAYAAVLAEAKRYQEAAELLDATDPRHPFDAELVSYVRGVLYFRTKRWPDVLAQFPEATQWRHPELKAAGAAMATTALASLGVFEEAFRRAQEAIEGDRVPGAANIALYTQGMCLRHVGREEEAVELLRRVYSRDAKFTPAREALDNPNFRLILTDPETIEARTDPWDPDSAPTRAQTEAARHAEMAAKYLAEGDAELNAMLGMEQAKKEIKLIKSTTKVNLARAKMGLPVPVTSRHTLLLGPPGTGKTSVARAFTKQLCGLTVLRKPLVVETSRTKLLGRYMADAEKNTEEMLEGALGGAVFFDEMHTLHEKGYSQGDPYGNAIINTLLLYMENHRDELVVFGAGYAKAMEKMLEVNQGLRRRFSTVIEFFSYTPQELIALTQLMGRENEDVITEEESQVLLPSYTKFYMEQSYSEDGDLIRGIDLLGNAGFVRNVVEKARDHRSFRLDDEDLDAVLASDLTEFSEDQLRRFKELTREDLAEGLRAAVAEKKTK

>CORE_REP|Org64_Gene4068#

MTAEPEVRTLREVVLDQLGTAESRAYKMWLPPLTNPVPLNELIARDRRQPLRFALGIMDEPRRHLQDVWGVDVSGAGGNIGIGGAPQTGKSTLLQTMVMSAAATHSPRNVQFYCIDLGGGGLIYLENLPHVGGVANRSEPDKVNRVVAEMQAVMRQRETTFKEHRVGSIGMYRQLRDDPSQPVASDPYGDVFLIIDGWPGFVGEFPDLEGQVQDLAAQGLAFGVHVIISTPRWTELKSRVRDYLGTKIEFRLGDVNETQIDRITREIPANRPGRAVSMEKHHLMIGVPRFDGVHSADNLVEAITAGVTQIASQHTEQTPPVRVLPERIHLHELDPNPPGPESDYRTRWEIPIGLRETDLTPAHCHMHTNPHLLIFGAAKSGKTTIAHAIARAICARNSPQQVRFMLADYRSGLLDAVPDTHLLGAGAINRNSASLDEAVQALAVNLKKRLPPTDLTTAQLRSRSWWSGFDVVLLVDDWHMIVGAAGGMPPMAPLAPLLPAAADIGLHIIVTCQMSQAYKATMDKFVGAAFGSGAPTMFLSGEKQEFPSSEFKVKRRPPGQAFLVSPDGKEVIQAPYIEPPEEVFAAPQAPVKIISLPV

>CORE_REP|Org9_Gene2717#

MNFAVLPPEVNSARIFAGAGLGPMLAAASAWDGLAEELHAAAGSFASVTTGLAGDAWHGPASLAMTRAASPYVGWLNTAAGQAAQAAGQARLAASAFEATLAATVSPAMVAANRTRLASLVAANLLGQNAPAIAAAEAEYEQIWAQDVAAMFGYHSAASAVATQLAPIQEGLQQQLQNVLAQLASGNLGSGNVGVGNIGNDNIGNANIGFGNRGDANIGIGNIGDRNLGIGNTGNWNIGIGITGNGQIGFGKPANPDVLVVGNGGPGVTALVMGGTDSLLPLPNIPLLEYAARFITPVHPGYTATFLETPSQFFPFTGLNSLTYDVSVAQGVTNLHTAIMAQLAAGNEVVVFGTSQSATIATFEMRYLQSLPAHLRPGLDELSFTLTGNPNRPDGGILTRFGFSIPQLGFTLSGATPADAYPTVDYAFQYDGVNDFPKYPLNVFATANAIAGILFLHSGLIALPPDLASGVVQPVSSPDVLTTYILLPSQDLPLLVPLRAIPLLGNPLADLIQPDLRVLVELGYDRTAHQDVPSPFGLFPDVDWAEVAADLQQGAVQGVNDALSGLGLPPPWQPALPRLF

>CORE_REP|Org156_Gene872#

MTTGGLVDENDGAAMRPLRHTLSQLRLHELLVEVQDRVEQIVEGRDRLDGLVEAMLVVTAGLDLEATLRAIVHSATSLVDARYGAMEVHDRQHRVLHFVYEGIDEETVRRIGHLPKGLGVIGLLIEDPKPLRLDDVSAHPASIGFPPYHPPMRTFLGVPVRVRDESFGTLYLTDKTNGQPFSDDDEVLVQALAAAAGIAVANARLYQQAKARQSWIEATRDIATELLSGTEPATVFRLVAAEALKLTAADAALVAVPVDEDMPAADVGELLVIETVGSAVASIVGRTIPVAGAVLREVFVNGIPRRVDRVDLEGLDELADAGPALLLPLRARGTVAGVVVVLSQGGPGAFTDEQLEMMAAFADQAALAWQLATSQRRMRELDVLTDRDRIARDLHDHVIQRLFAIGLALQGAVPHERNPEVQQRLSDVVDDLQDVIQEIRTTIYDLHGASQGITRLRQRIDAAVAQFADSGLRTSVQFVGPLSVVDSALADQAEAVVREAVSNAVRHAKASTLTVRVKVDDDLCIEVTDNGRGLPDEFTGSGLTNLRQRAEQAGGEFTLASVPGASGTVLRWSAPLSQ

>CORE_REP|Org260_Gene1921#

MARLSRERYAQLYGPTTGDRIRLADTNLLVEVTEDRCGGPGLAGDEAVFGGGKVLRESMGQGRASRADGAPDTVITGAVIIDYWGIIKADIGIRDGRIVGIGKAGNPDIMTGVHRDLVVGPSTEIISGNRRIVTAGTVDCHVHLICPQIIVEALAAGTTTIIGGGTGPAEGTKATTVTPGEWHLARMLESLDGWPVNFALLGKGNTVNPDALWEQLRGGASGFKLHEDWGSTPAAIDTCLAVADVAGVQVALHSDTLNETGFVEDTIGAIAGRSIHAYHTEGAGGGHAPDIITVAAQPNVLPSSTNPTRPHTVNTLDEHLDMLMVCHHLNPRIPEDLAFAESRIRPSTIAAEDVLHDMGAISMIGSDSQAMGRVGEVVLRTWQTAHVMKARRGALEGDPSGSQAADNNRVRRYIAKYTICPAIAHGMDHLIGSVEVGKLADLVLWEPAFFGVRPHVVLKGGAIAWAAMGDANASIPTPQPVLPRPMFGAAAATAAATSVHFVAPQSIDARLADRLAVNRGLAPVADVRAVGKTDLPLNDALPSIEVDPDTFTVRIDGQVWQPQPAAELPMTQRYFLF

>CORE_REP|Org375_Gene204#

MPQTTDEAASVSTVADIKPRSRDVTDGLEKAAARGMLRAVGMDDKDFAKPQIGVASSWNEITPCNLSLDRLANAVKEGVFSAGGYPLEFGTISVSDGISMGHEGMHFSLVSREVIADSVEVVMQAERLDGSVLLAGCDKSLPGMLMAAARLDLAAVFLYAGSILPGRAKLSDGSERDVTIIDAFEAVGACSRGLMSRADVDAIERAICPGEGACGGMYTANTMASAAEALGMSLPGSAAPPATDRRRDGFARRSGQAVVELLRRGITARDILTKEAFENAIAVVMAFGGSTNAVLHLLAIAHEANVALSLQDFSRIGSGVPHLADVKPFGRHVMSDVDHIGGVPVVMKALLDAGLLHGDCLTVTGHTMAENLAAITPPDPDGKVLRALANPIHPSGGITILHGSLAPEGAVVKTAGFDSDVFEGTARVFDGERAALDALEDGTITVGDAVVIRYEGPKGGPGMREMLAITGAIKGARLGKDVLLLTDGRFSGGTTGLCVGHIAPEAVDGGPIALLRNGDRIRLDVAGRVLDVLADPAEFASRQQDFSPPPPRYTTGVLSKYVKLVSSAAVGAVCG

>CORE_REP|Org395_Gene1984#

MFWVGGPCLMPASSAARCAARIVGGRCLMPASSAARYAARIVGGRCLMPASSAARCAARIVGGPRLYGMQRIIGTEVEYGISSPSDPTANPILTSTQAVLAYAAAAGIQRAKRTRWDYEVESPLRDARGFDLSRSAGPPPVVDADEVGAANMILTNGARLYVDHAHPEYSAPECTDPLDAVIWDKAGERVMEAAARHVASVPGAAKLQLYKNNVDGKGASYGSHENYLMSRQTPFSAIITGLTPFLVSRQVVTGSGRVGIGPSGDEPGFQLSQRSDYIEVEVGLETTLKRGIINTRDEPHADADRYRRLHVIIGDANLAETSTYLKLGTTALVLDLIEEGPAHAIDLTDLALARPVHAVHAISRDPSLRATVALADGRELTGLALQRIYLDRVAKLVDSRDPDPRAADIVETWAHVLDQLERDPMDCAELLDWPAKLRLLDGFRQRENLSWSAPRLHLVDLQYSDVRLDKGLYNRLVARGSMKRLVTEHQVLSAVENPPTDTRAYFRGECLRRFGADIAAASWDSVIFDLGGDSLVRIPTLEPLRGSKAHVGALLDSVDSAVELVEQLTAEPR

>CORE_REP|Org322_Gene4048#

MTDRLASLFESAVSMLPMSEARSLDLFTEITNYDESACDAWIGRIRCGDTDRVTLFRAWYSRRNFGQLSGSVQISMSTLNARIAIGGLYGDITYPVTSPLAITMGFAACEAAQGNYADAMEALEAAPVAGSEHLVAWMKAVVYGAAERWTDVIDQVKSAGKWPDKFLAGAAGVAHGVAAANLALFTEAERRLTEANDSPAGEACARAIAWYLAMARRSQGNESAAVALLEWLQTTHPEPKVAAALKDPSYRLKTTTAEQIASRADPWDPGSVVTDNSGRERLLAEAQAELDRQIGLTRVKNQIERYRAATLMARVRAAKGMKVAQPSKHMIFTGPPGTGKTTIARVVANILAGLGVIAEPKLVETSRKDFVAEYEGQSAVKTAKTIDQALGGVLFIDEAYALVQERDGRTDPFGQEALDTLLARMENDRDRLVVIIAGYSSDIDRLLETNEGLRSRFATRIEFDTYSPEELLEIANVIAAADDSALTAEAAENFLQAAKQLEQRMLRGRRALDVAGNGRYARQLVEASEQCRDMRLAQVLDIDTLDEDRLREINGSDMAEAIAAVHAHLNMRE

>CORE_REP|Org341_Gene3703#

MGRLFGTDGVRGVANRELTAELALALGAAAARRLSRSGAPGRRVAVLGRDPRASGEMLEAAVIAGLTSEGVDALRVGVLPTPAVAYLTGAYDADFGVMISASHNPMPDNGIKIFGPGGHKLDDDTEDQIEDLVLGVSRGPGLRPAGAGIGRVIDAEDATERYLRHVAKAATARLDDLAVVVDCAHGAASSAAPRAYRAAGARVIAINAEPNGRNINDGCGSTHLDPLRAAVLAHRADLGLAHDGDADRCLAVDANGDLVDGDAIMVVLALAMKEAGELACNTLVATVMSNLGLHLAMRSAGVTVRTTAVGDRYVLEELRAGDYSLGGEQSGHIVMPALGSTGDGIVTGLRLMTRMVQTGSSLSDLASAMRTLPQVLINVEVVDKATAAAAPSVRTAVEQAAAELGDTGRILLRPSXXXXDDSGDGGGSRRGRRPAAGGHGRRRGEHRALNRKCWNPDAARGVRIGMRPDSVNSAGIDIAAVYAVADRFSAAAELIDDAIGNHLTRLAFGGACAGRGHASRGDALRCRLDRLAGELSVWSRAAVQIAFALRAGANRYAEADLCAAARIG

>CORE_REP|Org412_Gene2483#

MPPKAADGRRPSPDGGLGGFVPFPADRAASYRAAGYWSGRTLDTVLSDAARRWPDRLAVADAGDRPGHGGLSYAELDQRADRAAAALHGLGITPGDRVLLQLPNGCQFAVALFALLRAGAIPVMCLPGHRAAELGHFAAVSAATGLVVADVASGFDYRPMARELVADHPTLRHVIVDGDPGPFVSWAQLCAQAGTGSPAPPADPGSPALLLVSGGTTGMPKLIPRTHDDYVFNATASAALCRLSADDVYLVVLAAGHNFPLACPGLLGAMTVGATAVFAPDPSPEAAFAAIERHGVTVTALVPALAKLWAQSCEWEPVTPKSLRLLQVGGSKLEPEDARRVRTALTPGLQQVFGMAEGLLNFTRIGDPPEVVEHTQGRPLCPADELRIVNADGEPVGPGEEGELLVRGPYTLNGYFAAERDNERCFDPDGFYRSGDLVRRRDDGNLVVTGRVKDVICRAGETIAASDLEEQLLSHPAIFSAAAVGLPDQYLGEKICAAVVFAGAPITLAELNGYLDRRGVAAHTRPDQLVAMPALPTTPIGKIDKRAIVRQLGIATGPVTTQRCH

>CORE_REP|Org394_Gene182#

MTAQLASHLTRALTLAQQQPYLARRQNWVNQLERHAMMQPDAPALRFVGNTMTWADLRRRVAALAGALSGRGVGFGDRVMILMLNRTEFVESVLAANMIGAIAVPLNFRLTPTEIAVLVEDCAAHVMLTEAALAPVAIGVRNIQPLLSVIVVAGGSSQDSVFGYEDLLNEAGDVHEPVDIPNDSPALIMYTSGTTGRPKGAVLTHANLTGQAMTALYTSGANINSDVGFVGVPLFHIAGIGNMLTGLLLGLPTVIYPLGAFDPGQLLDVLEAEKVTGIFLVPAQWQAVCTEQQARPRDLRLRVLSWGAAPAPDALLRQMSATFPETQILAAFGQTEMSPVTCMLLGEDAIAKRGSVGRVIPTVAARVVDQNMNDVPVGEVGEIVYRAPTLMSCYWNNPEATAEAFAGGWFHSGDLVRMDSDGYVWVVDRKKDMIISGGENIYCAELENVLASHPDIAEVAVIGRADEKWGEVPIAVAAVTNDDLRIEDLGEFLTDRLARYKHPKALEIVDALPRNPAGKVLKTELRLRYGACVNVERRSASAGFTERRENRQKL

>CORE_REP|Org161_Gene2826#

MPPRKKQAPQAPSTMKELKDTLWKAADKLRGSLSASQYKDVILGLVFLKYVSDAYDERREAIRAELAAEGMEESQIEDLIDDPEQYQGYGVFVVPVSARWKFLAENTKGKPAVGGEPAKNIGQLIDEAMDAVMKANPTLGGTLPRLYNKDNIDQRRLGELIDLFNSARFSRQGEHRARDLMGEVYEYFLGNFARAEGKRGGEFFTPPSVVKVIVEVLEPSSGRVYDPCCGSGGMFVQTEKFIYEHDGDPKDVSIYGQESIEETWRMAKMNLAIHGIDNKGLGARWSDTFARDQHPDVQMDYVMANPPFNIKDWARNEEDPRWRFGVPPANNANYAWIQHILYKLAPGGRAGVVMANGSMSSNSNGEGDIRAQIVEADLVSCMVALPTQLFRSTGIPVCLWFFAKDKAAGKQGSIDRCGQVLFIDARELGDLVDRAERALTNEEIVRIGDTFHAWRGSKSAAVKGIMYEDVPGFCKSATLAEIKATDYALTPGRYVGTPAVEDDGEPIDEKMARLSKALLEAFDESARLERVVREQLGRLR

>CORE_REP|Org270_Gene1830#

MAEESRGQRGSGYGLGLSTRTQVTGYQFLARRTAMALTRWRVRMEIEPGRRQTLAVVASVSAALVICLGALLWSFISPSGQLNESPIIADRDSGALYVRVGDRLYPALNLASARLITGRPDNPHLVRSSQIATMPRGPLVGIPGAPSSFSPKSPPASSWLVCDTVATSSSIGSLQGVTVTVIDGTPDLTGHRQILSGSDAVVLRYGGDAWVIREGRRSRIEPTNRAVLLPLGLTPEQVSQARPMSRALFDALPVGPELLVPEVPNAGGPATFPGAPGPIGTVIVTPQISGPQQYSLVLGDGVQTLPPLVAQILQNAGSAGNTKPLTVEPSTLAKMPVVNRLDLSAYPDNPLEVVDIREHPSTCWWWERTAGENRARVRVVSGPTIPVAATEMNKVVSLVKADTSGRQADQVYFGPDHANFVAVTGNNPGAQTSESLWWVTDAGARFGVEDSKEARDALGLTLTPSLAPWVALRLLPQGPTLSRADALVEHDTLPMDMTPAELVVPK

>CORE_REP|Org156_Gene3778#

MTLLLWSAVTGPSQWWRGGWAEPAGCCHGGFLDVVTRGEFGPDVRGWGPGSLSAAAAAWDELAAELWLAAASFESVCSGLADRWWQGPSSRMMAAQAARHTGWLAAAATQAEGAASQAQTMALAYEAAFAATVHPALVAANRALVAWLAGSNVFGQNTPAIAAAEAIYEQMWAQDVVAMLNYHAVASAVGARLRPWQQLLHELPRRLGGEHSDSTNTELANPSSTTTRITVPGASPVHAATLLPFIGRLLAARYAELNTAIGTNWFPGTTPEVVSYPATIGVLSGSLGAVDANQSIAIGQQMLHNEILAATASGQPVTVAGLSMGSMVIDRELAYLAIDPNAPPSSALTFVELAGPERGLAQTYLPVGTTIPIAGYTVGNAPESQYNTSVVYSQYDIWADPPDRPWNLLAGANALMGAAYFHDLTAYAAPQQGIEIAAVTSSLGGTTTTYMIPSPTLPLLLPLKQIGVPDWIVGGLNNVLKPLVDAGYSQYAPTAGPYFSHGNLVW

>CORE_REP|Org39_Gene3184#

MKNIGWMLRQRATVSPRLQAYVEPSTDVRMTYAQMNALANRCADVLTALGIAKGDRVALLMPNSVEFCCLFYGAAKLGAVAVPINTRLAAPEVSFILSDSGSKVVIYGAPSAPVIDAIRAQADPPGTVTDWIGADSLAERLRSAAADEPAVECGGDDNLFIMYTSGTTGHPKGVVHTHESVHSAASSWASTIDVRYRDRLLLPLPMFHVAALTTVIFSAMRGVTLISMPQFDATKVWSLIVEERVCIGGAVPAILNFMRQVPEFAELDAPDFRYFITGGAPMPEALIKIYAAKNIEVVQGYALTESCGGGTLLLSEDALRKAGSAGRATMFTDVAVRGDDGVIREHGEGEVVIKSDILLKEYWNRPEATRDAFDNGWFRTGDIGEIDDEGYLYIKDRLKDMIISGGENVYPAEIESVIIGVPGVSEVAVIGLPDEKWGEIAAAIVVADQNEVSEQQIVEYCGTRLARYKLPKKVIFAEAIPRNPTGKILKTVLREQYSATVPK

>CORE_REP|Org179_Gene4007#

MADPGGFLKYTHRKLPKRRPVPLRLRDWREVYEEFDNESLRQQATRCMDCGIPFCHNGCPLGNLIPEWNDLVRRGRWRDAIERLHATNNFPDFTGRLCPAPCEPACVLGINQDPVTIKQIELEIIDKAFDEGWVQPRPPRKLTGQTVAVVGSGPAGLAAAQQLTRAGHTVTVFEREDRIGGLLRYGIPEFKMEKRHLDRRLDQMRSEGTEFRPGVNVGVDISAEKLRADFDAVVLAGGATAWRELPIPGRELEGVHQAMEFLPWANRVQEGDDVLDEDGQPPITAKGKKVVIIGGGDTGADCLGTVHRQGAIAVHQFEIMPRPPDARAESTPWPTYPLMYRVSAAHEEGGERVFSVNTEAFVGTDGRVSALRAHEVTMLDGKFVKVEGSDFELEADLVLLAMGFVGPERAGLLTDLGVKFTERGNVARGDDFDTSVPGVFVAGDMGRGQSLIVWAIAEGRAAAAAVDRYLMGSSALPAPVKPTAAPLQ

>CORE_REP|Org59_Gene3250#

MDYAFLPPEINSARMYSGPGPNSMLVAAASWDALAAELASAAENYGSVIARLTGMHWWGPASTSMLAMSAPYVEWLERTAAQTKQTATQARAAAAAFEQAHAMTVPPALVTANRAELKALIASNLLGQNTAAIAAIEAQYAEMWAQDAAAMYGYATTSAAARQLTPFSSPQQTTNPAGLAAQNAAVTQAATNSAGNTPTALSQLSSFLSQAVEAPTGWPNILPDDFTILDGIFAAYATVGVTQDIESICAGIIGAENNLGLLGAASENPAELAPGAFGIDAALSSAEKGAAASMHDAVLASAGRAGSIGPMSVPPSWATPSSTPVSALSGAGLTTLDGTDVAEHGTPGLPGVPAGTDKRASGDPVAFFGFGGGVHHPLAVGHRDPDFHALSWRDPALRRPSCAPATTPDPDTRRVVLLAFNCHERPYDLDYNSFDSAAG

>CORE_REP|Org266_Gene3997#

MGLRLTTKVQVSGWRFLLRRLEHAIVRRDTRMFDDPLQFYSRSIALGIVVAVLILAGAALLAYFKPQGKLGGTSLFTDRATNQLYVLLSGQLHPVYNLTSARLVLGNPANPATVKSSELSKLPMGQTVGIPGAPYATPVSAGSTSIWTLCDTVARADSTSPVVQTAVIAMPLEIDASIDPLQSHEAVLVSYQGETWIVTTKGRHAIDLTDRALTSSMGIPVTARPTPISEGMFNALPDMGPWQLPPIPAAGAPNSLGLPDDLVIGSVFQIHTDKGPQYYVVLPDGIAQVNATTAAALRATQAHGLVAPPAMVPSLVVRIAERVYPSPLPDEPLKIVSRPQDPALCWSWQRSAGDQSPQSTVLSGRHLPISPSAMNMGIKQIHGTATVYLDGGKFVALQSPDPRYTESMYYIDPQGVRYGVPNAETAKSLGLSSPQNAPWEIVRLLVDGPVLSKDAALLEHDTLPADPSPRKVPAGASGAP

>CORE_REP|Org50_Gene2330#

MTEKTPDDVFKLAKDEKVEYVDVRFCDLPGIMQHFTIPASAFDKSVFDDGLAFDGSSIRGFQSIHESDMLLLPDPETARIDPFRAAKTLNINFFVHDPFTLEPYSRDPRNIARKAENYLISTGIADTAYFGAEAEFYIFDSVSFDSRANGSFYEVDAISGWWNTGAATEADGSPNRGYKVRHKGGYFPVAPNDQYVDLRDKMLTNLINSGFILEKGHHEVGSGGQAEINYQFNSLLHAADDMQLYKYIIKNTAWQNGKTVTFMPKPLFGDNGSGMHCHQSLWKDGAPLMYDETGYAGLSDTARHYIGGLLHHAPSLLAFTNPTVNSYKRLVPGYEAPINLVYSQRNRSACVRIPITGSNPKAKRLEFRSPDSSGNPYLAFSAMLMAGLDGIKNKIEPQAPVDKDLYELPPEEAASIPQTPTQLSDVIDRLEADHEYLTEGGVFTNDLIETWISFKRENEIEPVNIRPHPYEFALYYDV

>CORE_REP|Org17_Gene3350#

MSWPAAAVDRVIKAYDVRGLVGEEIDESLVTDLGAAFARLMRTEDARPVVIGHDMRDSSPSLADAFAAGVTGQGLDVVRVGLASTDQLYFASGLLDCPGAMFTASHNPAAYNGIKMCRAAAKPVGADTGLTAIRDDLIAGVARYDGTPGTIADQDVLVDYGAFLRSLVDTSGLRPLRVAVDAGNGMAGHTAPAVLGVIDSITLLPSYFELDGSFPNHEANPLDPANLVDLQAYVRDTGADIGLAFDGDADRCFVVDERGQPVSPSTVTALVAARELNREIGATIIHNVITSRAVPELVAERGGTPLRSRVGHSYIKALMAETGAIFGGEHSAHYYFRDFWGADSGMLAALHVLAALGEQSRPLSELTADYQRYESSGEINFTVVDSSACVEAVLKSFGNRIVSIDHLDGVTVDLGDDSWFNLRSSNTEPLLRLNVEGRSVGDVDAVVRQVSAEIAAQSAHAKAGP

>CORE_REP|Org184_Gene547#

MGSTEQATSRVRGAARTSAQLFEAACSVIPGGVNSPVRAFTAVGGTPRFITEAHGCWLIDADGNRYVDLVCSWGPMILGHAHPAVVEAVAKAAARGLSFGAPTPAETQLAGEIIGRVAPVERIRLVNSGTEATMSAVRLARGFTGRAKIVKFSGCYHGHVDALLADAGSGVATLGLCDDPQRPASPRSQSSRGLPSSPGVTGAAAADTIVLPYNDIDAVQQTFARFGEQIAAVITEASPGNMGVVPPGPGFNAALRAITAEHGALLILDEVMTGFRVSRSGWYGIDPVPADLFAFGKVMSGGMPAAAFGGRAEVMQRLAPLGPVYQAGTLSGNPVAVAAGLATLRAADDAVYTALDANADRLAGLLSEALTDAVVPHQISRAGNMLSVFFGETPVTDFASARASQTWRYPAFFHAMLDAGVYPPCSAFEAWFVSAALDDAAFGRIANALPAAARAAAQERPA

>CORE_REP|Org41_Gene2297#

MNWTVDIPIDQLPSLPPLPTDLRTRLDAALAKPAAQQPTWPADQALAMRTVLESVPPVTVPSEIVRLQEQLAQVAKGEAFLLQGGDCAETFMDNTEPHIRGNVRALLQMAVVLTYGASMPVVKVARIAGQYAKPRSADIDALGLRSYRGDMINGFAPDAAAREHDPSRLVRAYANASAAMNLVRALTSSGLASLHLVHDWNREFVRTSPAGARYEALATEIDRGLRFMSACGVADRNLQTAEIYASHEALVLDYERAMLRLSDGEDGEPQLFDLSAHTVWIGERTRQIDGAHIAFAQVIANPVGVKLGPNMTPELAVEYVERLDPHNKPGRLTLVSRMGNHKVRDLLPPIVEKVQATGHQVIWQCDPMHGNTHESSTGFKTRHFDRIVDEVQGFFEVHRALGTHPGGIHVEITGENVTECLGGAQDISETDLAGRYETACDPRLNTQQSLELAFLVAEMLRD

>CORE_REP|Org32_Gene865#

MTALDWRSALTADEQRSVRALVTATTAVDGVAPVGEQVLRELGQQRTEHLLVAGSRPGGPIIGYLNLSPPRGAGGAMAELVVHPQSRRRGIGTAMARAALAKTAGRNQFWAHGTLDPARATASALGLVGVRELIQMRRPLRDIPEPTIPDGVVIRTYAGTSDDAELLRVNNAAFAGHPEQGGWTAVQLAERRGEAWFDPDGLILAFGDSPRERPGRLLGFHWTKVHPDHPGLGEVYIGPSGCGKTTVLRTLNRMHEVIPGARVEGAVLLDDQDIYAPGIDPVGVRRAIGMVFQRPNPFPAMSIRNNVVAGLKLQGVRNRKVLDDTAESSLRGANLWDEVKDRLDKPGGGLSGGQQQRLCIARAIAVQPDVLLMDEPCSSLDPISTMAIEDLISELKQQYTIVIVTHNMQQAARVSDQTAFFNLEAVGKPGRLVEIASTEKIFSNPNQKATEDYISGRFG

>CORE_REP|Org23_Gene2493#

MSELSVATGAVSTASSSIPMPAGVNPADLAAELAAVVTESVDEDYLLYECDGQWVLAAGVQAMVELDSDELRVIRDGVTRRQQWSGRPGAALGEAVDRLLLETDQAFGWVAFEFGVHRYGLQQRLAPHTPLARVFSPRTRIMVSEKEIRLFDAGIRHREAIDRLLATGVREVPQSRSVDVSDDPSGFRRRVAVAVDEIAAGRYHKVILSRCVEVPFAIDFPLTYRLGRRHNTPVRSFLLQLGGIRALGYSPELVTAVRADGVVITEPLAGTRALGRGPAIDRLARDDLESNSKEIVEHAISVRSSLEEITDIAEPGSAAVIDFMTVRERGSVQHLGSTIRARLDPSSDRMAALEALFPAVTASGIPKAAGVEAIFRLDECPRGLYSGAVVMLSADGGLDAALTLRAAYQVGGRTWLRAGAGIIEESEPEREFEETCEKLSTLTPYLVARQ

>CORE_REP|Org342_Gene2191#

MGIETEFGVTCTFHGHRRLSPDEVARYLFRRVVSWGRSSNVFLRNGARLYLDVGSHPEYATAECDSLVQLVTHDRAGEWVLEDLLVDAEQRLADEGIGGDIYLFKNNTDSAGNSYGCHENYLIVRAGEFSRISDVLLPFLVTRQLICGAGKVLQTPKAATYCLSQRAEHIWEGVSSATTRSRPIINTRDEPHADAEKYRRLHVIVGDSNMSETTTMLKVGTAALVLEMIESGVAFRDFSLDNPIRAIREVSHDVTGRRPVRLAGGRQASALDIQREYYTRAVEHLQTREPNAQIEQVVDLWGRQLDAVESQDFAKVDTEIDWVIKRKLFQRYQDRYDMELSHPKIAQLDLAYHDIKRGRGIFDLLQRKGLAARVTTDEEIAEAVDQPPQTTRARLRGEFISAAQEAGRDFTVDWVHLKLNDQAQRTVLCKDPFRAVDERVKRLIASM

>CORE_REP|Org418_Gene1346#

MNELLHLAPNVWPRNTTRDEVGVVCIAGIPLTQLAQEYGTPLFVIDEDDFRSRCRETAAAFGSGANVHYAAKAFLCSEVARWISEEGLCLDVCTGGELAVALHASFPPERITLHGNNKSVSELTAAVKAGVGHIVVDSMTEIERLDAIAGEAGIVQDVLVRLTVGVEAHTHEFISTAHEDQKFGLSVASGAAMAAVRRVFATDHLRLVGLHSHIGSQIFDVDGFELAAHRVIGLLRDVVGEFGPEKTAQIATVDLGGGLGISYLPSDDPPPIAELAAKLGTIVSDESTAVGLPTPKLVVEPGRAIAGPGTITLYEVGTVKDVDVSATAHRRYVSVDGGMSDNIRTALYGAQYDVRLVSRVSDAPPVPARLVGKHCESGDIIVRDTWVPDDIRPGDLVAVAATGAYCYSLSSRYNMVGRPAVVAVHAGNARLVLRRETVDDLLSLEVR

>CORE_REP|Org151_Gene2329#

MDRQKEFVLRTLEERDIRFVRLWFTDVLGFLKSVAIAPAELEGAFEEGIGFDGSSIEGFARVSESDTVAHPDPSTFQVLPWATSSGHHHSARMFCDITMPDGSPSWADPRHVLRRQLTKAGELGFSCYVHPEIEFFLLKPGPEDGSVPVPVDNAGYFDQAVHDSALNFRRHAIDALEFMGISVEFSHHEGAPGQQEIDLRFADALSMADNVMTFRYVIKEVALEEGARASFMPKPFGQHPGSAMHTHMSLFEGDVNAFHSADDPLQLSEVGKSFIAGILEHACEISAVTNQWVNSYKRLVQGGEAPTAASWGAANRSALVRVPMYTPHKTSSRRVEVRSPDSACNPYLTFAVLLAAGLRGVEKGYVLGPQAEDNVWDLTPEERRAMGYRELPSSLDSALRAMEASELVAEALGEHVFDFFLRNKRTEWANYRSHVTPYELRTYLSL

>CORE_REP|Org341_Gene1815#

MDFGALPPEVNSGRMYCGPGSAPMVAAASAWNGLAAELSVAAVGYERVITTLQTEEWLGPASTLMVEAVAPYVAWMRATAIQAEQAASQARAAAAAYETAFAAIVPPPLIAANRARLTSLVTHNVFGQNTASIAATEAQYAEMWAQDAMAMYGYAGSSATATKVTPFAPPPNTTSPSAAATQLSAVAKAAGTSAGAAQSAIAELIAHLPNTLLGLTSPLSSALTAAATPGWLEWFINWYLPISQLFYNTVGLPYFAIGIGNSLITSWRALGWIGPEAAEAAAAXXXAGGRRRRGWRYRTRIRGSRQRGHHRQVIAAAQLGRGQSEPSPDRGFGLRTPGQRYRRATGGRCGGKPVGRHAASRFGHRYGGCGSPLRVPGYGDVPAAVCRITRGLPYPRTRGRHICRTISRLPTRQTPSRTCVGTLPTAIFS

>CORE_REP|Org427_Gene486#

MSVVGTPKSAEQIQQEWDTNPRWKDVTRTYSAEDVVALQGSVVEEHTLARRGAEVLWEQLHDLEWVNALGALTGNMAVQQVRAGLKAIYLSGWQVAGDANLSGHTYPDQSLYPANSVPQVVRRINNALQRADQIAKIEGDTSVENWLAPIVADGEAGFGGALNVYELQKALIAAGVAGSHWEDQLASEKKCGHLGGKVLIPTQQHIRTLTSARLAADVADVPTVVIARTDAEAATLITSDVDERDQPFITGERTREGFYRTKNGIEPCIARAKAYAPFADLIWMETGTPDLEAARQFSEAVKAEYPDQMLAYNCSPSFNWKKHLDDATIAKFQKELAAMGFKFQFITLAGFHALNYSMFDLAYGYAQNQMSAYVELQEREFAAEERGYTATKHQREVGAGYFDRIATTVDPNSSTTALTGSTEEGQFH

>CORE_REP|Org379_Gene4017#

MASGSGLCKTTSNFIWGQLLLLGEGIPDPGDIFNTGSSLFKQISDKMGLAIPGTNWIGQAAEAYLNQNIAQQLRAQVMGDLDKLTGNMISNQAKYVSDTRDVLRAMKKMIDGVYKVCKGLEKIPLLGHLWSWELAIPMSGIAMAVVGGALLYLTIMTLMNATNLRGILGRLIEMLTTLPKFPGLPGLPSLPDIIDGLWPPKLPDIPIPGLPDIPGLPDFKWPPTPGSPLFPDLPSFPGFPGFPEFPAIPGFPALPGLPSIPNLFPGLPGLGDLLPGVGDLGKLPTWTELAALPDFLGGFAGLPSLGFGNLLSFASLPTVGQVTATMGQLQQLVAAGGGPSQLASMGSQQAQLISSQAQQGGQQHATLVSDKKEDEEGVAEAERAPIDAGTAASQRGRRGPSFDRTPSRQQVCAIASRSHSE

>CORE_REP|Org341_Gene1575#

MTASVNSLDLAAIRADFPILKRIMRGGNPLAYLDSGATSQRPLQVLDAEREFLTASNGAVHRGAHQLMEEATDAYEQGRADIALFVGADTDELVFTKNATEALNLVSYVLGDSRFERAVGPGDVIVTTELEHHANLIPWQELARRTGATLRWYGVTDDGRIDLDSLYLDDRVKVVAFTHHSNVTGVLTPVSELVSRAHQSGALTVLDACQSVPHQPVDLHELGVDFAAFSGHKMLGPNGIGVLYGRRELLAQMPPFLTGGSMIETVTMEGATYAPAPQRFEAGTPMTSQVVGLAAAARYLGAIGMAAVEAHERELVXXXXAAIEGLSGIDGVRILGPTSMRDRGSPVAFVVEGVHAHDVGQVLDDGGVAVRVGHHCALPLHRRFGLAATARASFAVYNTADEVDRLVAGVRRSRHFFGRA

>CORE_REP|Org27_Gene2517#

MLQSDSVTVTLCSPTEDDWPGMFLLAAASFTDFIGPESATAWRTLVPTDGAVVVRDGAGPGSEVVGMALYMDLRLTVPGEVVLPTAGLSFVAVAPTHRRRGLLRAMCAELHRRIADSGYPVAALHASEGGIYGRFGYGPATTLHELTVDRRFARFHADAPGGGLGGSSVRLVRPTEHRGEFEAIYERWRQQVPGGLLRPQVLWDELLAECKAAPGGDRESFALLHPDGYALYRVDRTDLKLARVSELRAVTADAHCALWRALIGLDSMERISIITHPQDPLPHLLTDTRLARTTWRQDGLWLRIMNVPAALEARGYAHEVGEFSTVLEVSDGGRFALKIGDGRARCTPTDAAAEIEMDRDVLGSLYLGAHRASTLAAANRLRTKDSQLLRRLDAAFASDVPVQTAFEF

>CORE_REP|Org111_Gene3606#

MKRFWENVGKPNDTTDGRGTTSLAMTPISQTPGLLAEAMVDLGAIEHNVRVLREHAGHAQLMAVVKADGYGHGATRVAQTALGAGAAELGVATVDEALALRADGITAPVLAWLHPPGIDFGPALLADVQVAVSSLRQLDELLHAVRRTGRTATVTVKVDTGLNRNGVGPAQFPAMLTALRQAMAEDAVRLRGLMSHMVYADKPDDSINDVQAQRFTAFLAQAREQGVRFEVAHLSNSSATMARPDLTFDLVRPGIAVYGLSPVPALGDMGLVPAMTVKCAVALVKSIRAGEGVSYGHTWIAPRDTNLALLPIGYADGVFRSLGGRLEVLINGRRCPGVGRICMDQFMVDLGPGPLDVAEGDEAILFGPGIRGEPTAQDWADLVGTIHYEVVTSPRGRITRTYREAENR

>CORE_REP|Org289_Gene3572#

MSAGRTRKTSSLTARIECPLLRVTEFCREQPMTKSLPGVADLRLGANHPRMWTRRVQGTVVNVGVKVLPWIPTPAKRILXAGRSVIIDGNTLDPTLQLMLSTSRIFGVDGLAXDXXXXXXXXXXRAXCEAMPGPQIHVDVTDLSIPGPAGEIPARHYRPSGGGAXPXLVFYHGGGWTLGDLDTHDAXXRLTCRDADIQVLSIDYRLAPEHPAPAAVEDAYAAFVWAHEHASDEFGALPGRVAVGGDSAGXXLXAVVCQLARDKARYEGGPTPVLQWLLYPRTDFTAQTRSMGLFGNGFLLTKRDIDWFHTQYLRDSDVDPADPRLSPLLAESLSGLAPALIAVAGFDPLRDEGESYAKALRAAGTAVDLRYLGSLTHGFLNLFQLGGGSAAGTNELISAXXAHLXRV

>CORE_REP|Org40_Gene2051#

MASVAQPVRRRPKDRKKQILDQAVGLFIERGFHSVKLEDIAEAAGVTARALYRHYDNKQALLAEAIRTGQDQYQSARRLTEGETEPTPRPLNADLEDLIAAAVASRALTVLWQREARYLNEDDRTAVRRRINAIVAGMRDSVLLEVPDLSPQHSELRAWAVSSTLTSLGRHSLSLPGEELKKLLYQACMAAARTPPVCELPPLPAGDAARDEADVLFSRYETLLAAGARLFRAQGYPAVNTSEIGKGAGIAGPGLYRSFSSKQAILDALIRRLDEWRCLECIRALRANQQAAQRLRGLVQGHVRISLDAPDLVAVSVTELSHASVEVRDGYLRNQGDREAVWIDLIGKLVPATSVAQGRLLVAAAISFIEDVARTWHLTRYAGVADEISGLALAILTSGAGNLLRA

>CORE_REP|Org289_Gene489#

MSSTVLVINSGSSSLKFQLXXXXXGMSXAAGIVERIGERSSPXADHAQALHRAFKMLAEDGIDLXTCGLVAVGHRVVHGGTEFHQPTLLDDTVIGKLEELSALAPLHNPXAVLGIKVARRLLANVAHVAVFDTAFFHDLPPAAATYAIDXDVADRWHIRRYGFHGTSHQYVSXXAXAFLGRPLDGLNQIVLHLGNGASASAIARGRPVETSMGLTPLEGLVMGTRSGDLDPGVISYLWRTARMGVEDIESMLNHRSGMLGLAGERDFRRLRLVIETGDRSAQLAYEVFIHRLRKYLGAYLAVLGHTDVVSFTAGIGENDAAVRRDALAGLQGLGIALDQDRNLGPGHGARRISSDDSPIAVLVVPTNEELAIARDCLRVLGGRRAXIIXXXAXVSXXAIHTRAA

>CORE_REP|Org125_Gene1870#

MTAALDFATLPPEINSARMYSGAGSAPMLAAASAWHGLSAELRASALSYSSVLSTLTGEEWHGPASASMTAAAAPYVAWMSVTAVRAEQAGAQAEAAAAAYEAAFAATVPPPVIEANRAQLMALIATNVLGQNAPAIAATEAQYAEMWSQDAMAMYGYAGASAAATQLTPFTEPVQTTNASGLAAQSAAIAHATGASAGAQQTTLSQLIAAIPSVLQGLSSSTAATSASGPSGLLGILGSGSSWLDKLWALLDPNSNFWNTIASSGLFLPSNTIAPFLGLLGGVAAADAAGDVLGEATSGGLGGALVAPLGSAGGLGGTVAAGLGNAATVGTLSVPPSWTAAAPLASPLGSALGGTPMVAPPPAVAAGMPGMPFGTMGGQGFGRAVPQYGFRPNFVARPPAAG

>CORE_REP|Org242_Gene2710#

MGVELGSNSEVGALRVVILHRPGAELRRLTPRNTDQLLFDGLPWVSRAQDEHDEFAELLASRGAEVLLLSDLLTEALHHSGAARMQGIAAAVDAPRLGLPLAQELSAYLRSLDPGRLAHVLTAGMTFNELPSDTRTDVSLVLRMHHGGDFVIEPLPNLVFTRDSSIWIGPRVVIPSLALRARVREASLTDLIYAHHPRFTGVRRAYESRTAPVEGGDVLLLAPGVVAVGVGERTTPAGAEALARSLFDDDLAHTVLAVPIAQQRAQMHLDTVCTMVDTDTMVMYANVVDTLEAFTIQRTPDGVTIGDAAPFAEAAAKAMGIDKLRVIHTGMDPVVAEREQWDDGNNTLALAPGVVVAYERNVQTNARLQDAGIEVLTIAGSELGTGRGGPRCMSCPAARDPL

>CORE_REP|Org155_Gene3928#

MQPMTARFDLFVVGSGFFGLTIAERVATQLDKRVLVLERRPHIGGNAYSEAEPQTGIEVHKYGAHLFHTSNKRVWDYVRQFTDFTDYRHRVFAMHNGQAYQFPMGLGLVSQFFGKYFTPEQARQLIAEQAAEIDTADAQNLEEKAISLIGRPLYEAFVKGYTAKQWQTDPKELPAANITRLPVRYTFDNRYFSDTYEGLPTDGYTAWLQNMAADHRIEVRLNTDWFDVRGQLRPGSPAAPVVYTGPLDRYFDYAEGRLGWRTLDFEVEVLPIGDFQGTAVMNYNDLDVPYTRIHEFRHFHPERDYPTDKTVIMREYSRFAEDDDEPYYPINTEADRALLATYRARAKSETASSKVLFGGRLGTYQYLDMHMAIASALNMYDNVLAPHLRDGVPLLQDGA

>CORE_REP|Org125_Gene369#

MAQREWVEKDFYQELGVSSDASPEEIKRAYRKLARDLHPDANPGNPAAGERFKAVSEAHNVLSDPAKRKEYDETRRLFAGGGFGGRRFDSGFGGGFGGFGVGGDGAEFNLNDLFDAASRTGGTTIGDLFGGLFGRGGSARPSRPRRGNDLETETELDFVEAAKGVAMPLRLTSPAPCTNCHGSGARPGTSPKVCPTCNGSGVINRNQGAFGFSEPCTDCRGSGSIIEHPCEECKGTGVTTRTRTINVRIPPGVEDGQRIRLAGQGEAGLRGAPSGDLYVTVHVRPDKIFGRDGDDLTVTVPVSFTELALGSTLSVPTLDGTVGVRVPKGTADGRILRVRGRGVPKRSGGSGDLLVTVKVAVPPNLAGAAQEALEAYAAAERSSGFNPRAGWAGNR

>CORE_REP|Org55_Gene1299#

MAEIVLDHVNKSYPDGHTAVRDLNLTIADGEFLILVGPSGCGKTTTLNMIAGLEDISSGELRIAGERVNEKAPKDRDIAMVFQSYALYPHMTVRQNIAFPLTLAKMRKADIAQKVSETAKILDLTNLLDRKPSQLSGGQRQRVAMGRAIVRHPKAFLMDEPLSNLDAKLRVQMRGEIAQLQRRLGTTTVYVTHDQTEAMTLGDRVVVMYGGIAQQIGTPEELYERPANLFVAGFIGSPAMNFFPARLTAIGLTLPFGEVTLAPEVQGVIAAHPKPENVIVGVRPEHIQDAALIDAYQRIRALTFQVKVNLVESLGADKYLYFTTESPAVHSVQLDELAEVEGESALHENQFVARVPAESKVAIGQSVELAFDTARLAVFDADSGANLTIPHRA

>CORE_REP|Org40_Gene3626#

MQLTPTTGHIPAKRAENVLVLRTPAPAIVGMLEFRFVLGRGSIEITESGPSVAGRLAALASRMTIKPLMTVGSYLSPLPLPLGFVDFACRVWRPGQGTVRTTINLPNATAQLVRAPGVRAADGAGRVVLYLHGGAFVMCGPNSHSRIVNALSGFAESPVLIVDYRLIPKHSLGMALDDCHDAYQWLRARGYRPEQIVLAGDSAGGYLALALAQRLQCDDEKPAAIVAISPLLQLAKGPKQDHPNIGTDAMFPARAFDALAAWVRAAAAKNMVDGRPEDLYEPLDHIESSLPPTLIHVSGSEVLLHDAQLGAGKLAAAGVCAEVRVWPGQAHLFQLATPLVPEATRSLRQIGQFIRDATADSSLSPVHRSRYVAGSPRAASRGAFGQSPI

>CORE_REP|Org89_Gene3101#

MFGGRSNEHAISCVSAGSILRNLDSRRFDVIAVGITPAGSWVLTDANPDALTITNRELPQVKSGSGTELALPADPRRGGQLVSLPPGAGEVLESVDVVFPVLHGPYGEDGTIQGLLELAGVPYVGAGVLASAVGMDKEFTKKLLAADGLPVGAYAVLRPPRSTLHRQECERLGLPVFVKPARGGSSIGVSRVSSWDQLPAAVARARRHDPKVIVEAAISGRELECGVLEMPDGTLEASTLGEIRVAGVRGREDSFYDFATKYLDDAAELDVPAKVDDQVAEAIRQLAIRAFAAIDCRGLARVDFFLTDDGPVINEINTMPGFTTISMYPRMWAASGVDYPTLLATMIETALARGVGLHRCRPAAPGASAALAIGTSPMVPTRRARR

>CORE_REP|Org384_Gene2526#

MRSPHRDAIRTARGLVVKVGTTALTTPSGMFDAGRLAGLAEAVERRMKAGSDVVIVSSGAIAAGIEPLGLSRRPKDLATKQAAASVGQVALVNSWSAAFARYGRTVGQVLLTAHDISMRVQHTNAQRTLDRLRALHAVAIVNENDTVATNEIRFGDNDRLSALVAHLVGADALVLLSDIDGLYDCDPRKTADATFIPEVSGPADLDGVVAGRSSHLGTGGMASKVSAALLAADAGVPVLLAPAADAATALADASVGTVFAARPARLSARRFWVRYAAEATGALTLDAGAVRAVVRQRRSLLAAGITAVSGRFCGGDVVELRAPDAAMVARGVVAYDASELATMVGRSTSELPGELRRPVVHADDLVAVSAKQAKQV

>CORE_REP|Org5_Gene3290#

MATLHPEPPFALCGPRGTLIARGVRTRYCDVRAAQAALRSGTAPILLGALPFDVSRPAALMVPDGVLRARKLPDWPTGPLPKVRVAAALPPPADYLTRIGRARDLLAAFDGPLHKVVLARAVQLTADAPLDARVLLRRLVVADPTAYGYLVDLTSAGNDDTGAALVGASPELLVARSGNRVMCKPFAGSAPRAADPKLDAANAAALASSAKNRHEHQLVVDTMRVALEPLCEDLTIPAQPQLNRTAAVWHLCTAITGRLRNISTTAIDLALALHPTPAVGGVPTKAATELIAELEGDRGFYAGAVGWCDGRGDGHWVVSIRCAQLSADRRAALAHAGGGIVAESDPDDELEETTTKFATILTALGVEQ

>CORE_REP|Org13_Gene2296#

MALSAEGSSGGSRGGSPKAEAASVPSWPQILGRLTDNRDLARGQAAWAMDQIMTGNARPAQIAAFAVAMTMKAPTADEVGELAGVMLSHAHPLPADTVPDDAVDVVGTGGDGVNTVNLSTMAAIVVAAAGVPVVKHGNRAASSLSGGADTLEALGVRIDLGPDLVARSLAEVGIGFCFAPRFHPSYRHAAAVRREIGVPTVFNLLGPLTNPARPRAGLIGCAFADLAEVMAGVFAARRSSVLVVHGDDGLDELTTTTTSTIWRVAAGSVDKLTFDPAGFGFARAQLDQLAGGDAQANAAAVRAVLGGARGPVRDAVVLNAAGAIVAHAGLSSRAEWLPAWEEGLRRASAAIDTGAAEQLLARWVRFGRQI

>CORE_REP|Org122_Gene1941#

MSKLQLRAVVADRRLDVEFSVSAGEVLAVLGPNGAGKSTALHVIAGLLRPDAGLVRLGDRVLTDTEAGVNVATHDRRVGLLLQDPLLFPHLSVAKNVAFGPQCRRGMFGSGRARTRASALRWLREVNAEQFADRKPRQLSGGQAQRVAIARALAAEPDVLLLDEPLTGLDVAAAAGIRSVLRSVVARSGCAVVLTTHDLLDVFTLADRVLVLESGTIAEIGPVADVLTAPRSRFGARIAGVNLVNGTIGPDGSLRTQSGAHWYGTPVQDLPTGHEAIAVFPPTAVAVYPEPPHGSPRNIVGLTVAEVDTRGPTVLVRGHDQPGGAPGLAACITVDAATELRVAPGSRVWFSVKAQEVALHPAPHQHASS

>CORE_REP|Org395_Gene2755#

MVPELMFDEPRPGRPPRHLADLDAAGRASAVAELGLPAFRAKQLAHQYYGRLIADPRQMTDLPAAVRDRIAGAMFPNLLTASADITCDAGQTRKTLWRAVDGTMFESVLMRYPRRNTVCISSQAGCGMACPFCATGQGGLTRNLSTAEILEQVRAGAAALRDDFGDRLSNVVFMGMGEPLANYARVLAAVQRITARPPSGFGISARAVTVSTVGLAPAIRNLADARLGVTLALSLHAPDDGLRDTLVPVNNRWRISEALDAARYYANVTGRRVSIEYALIRDVNDQPWRADLLGKRLHRVLGPLAHVNLIPLNPTPGSDWDASPKPVEREFVKRVRAKGVSCTVRDTRGREISAACGQLAAVGG

>CORE_REP|Org41_Gene2955#

MANVQYSAVTQRYPGADAPTVDNLDLDIADGEFLVLVGPSGCGKSTTLRVLAGLEPIESGRISIGDVDVTHLPPRARDVAMVFQNYALYPNMTVAANMGFALRNAGMSRADTRRRVLEVADMLELTDLLDRKPAKLSGGQRQRVAMGRAIVRRPRVFCMDEPLSNLDAKLRVSTRSQISGLQRRLGTTTVYVTHDQVEAMTMGDRVAVLKDGVLQQVDTPRALYDDPVNTFVATFIGAPAMNLIDAAVAHGVVRAPDLAIPVPDPAAERVLVGVRPESWDVASIGTPGSLTVHVELVEELGFESFVYATPVDQRGWSSRAPRIVFRTDRRTAVRVGESLAIVPHSQEVRLFNSRTETRLR

>CORE_REP|Org289_Gene1824#

MVDPTATDSPKVSIVSISYNQEEYIREALDGFAAQRTEFPVEVIIADDASTDATPRIIGEYAARYPQLFRPILRQTNIGVHANFKDVLSAARGEYLALCEGDDYWTDPLKLSKQVKYLDRHPETTVCFHPVRVIYEDGAKDSEFPPLSWRRDLSVDALLARNFIQTNSVVYRRQPSYDDIPANVMPIDWYLHVRHAVGGEIAMLPETMAVYRRHAHGIWHSAYTXXRKFWETRGHGMAATLEAMLDLVHGHREREAIVGEVSAWVLREXXXTPGXQGRALLLKSIADHXRMTMLSLQHRWAQTPWRRFKRRLSTELSSLAALAYXTRRRALEGRDGGYRETTSPPTGRGRNVRGSHA

>CORE_REP|Org180_Gene2467#

MTSRETRAADAAGARQADAQVRSSIDVPPDLVVGLLGSADENLRALERTLSADLHVRGNAVTLCGEPADVALAERVISELIAIVASGQSLTPEVVRHSVAMLVGTGNESPAEVLTLDILSRRGKTIRPKTLNQKRYVDAIDANTIVFGIGPAGTGKTYLAMAKAVHALQTKQVTRIILTRPAVEAGERLGFLPGTLSEKIDPYLRPLYDVLYDMMDPELIPKLMSAGVIEVAPLAYMRGRTLNDAFIVLDEAQNTTAEQMKMFLTRLGFGSKVVVTGDVTQIDLPGGARSGLRAAVDILEDIDDIHIAELTSVDVVRHRLVSEIVDAYARYEEPGSGLNRAARRASGARGRR

>CORE_REP|Org163_Gene998#

MACERLGGQSGAADVDAAAPAMAAVNLTLGFAGKTVLDQVSMGFPARAVTSLMGPTGSGKTTFLRTLNRMNDKVSGYRYSGDVLLGGRSIFNYRDVLEFRRRVGMLFQRPNPFPMSIMDNVLAGVRAHKLVPRKEFRGVAQARLTEVGLWDAVKDRLSDSPFRLSGGQQQLLCLARTLAVNPEVLLLDEPTSALDPTTTEKIEEFIRSLADRLTVIIVTHNLAQAARISDRAALFFDGRLVEEGPTEQLFSSPKHAENRPIRRRTVGGRQGRQARKLKSTERYGVKIRLHTLLAVLTAAPLLLAAAGCGSKPPSGSPETGAGAGTVATTPRVVAGDVGGDR

>CORE_REP|Org404_Gene4037#

MKNLWNDPNMLDDGAIGRGDPSVRHHFRDSVSDTMRITDLAAPRKIPPGTGWRKFVYSVSFHKINPGESPRERHYRNLQGRIRRQYVITVVSGKGGVGVTTMAACIGGVFRECRPENVIAIDAVPSFGTLADRIDESPPGDYAAIINDTDVQGYADIREHLGQNTVGLDVLAGNRTSDQPRPLVPAMFSAVLSRLRRTHTVIVIDTSPDLEHDVMKAVLQSTDTLVFVSGITADRSRPVLRAVDYLRAQGYHELVSRSTVILNHTDSITDKDALAYLTERFTKVGAIVEAMPFDPHLAKGGIIDTVHELNKKSRLRLFEITAGLADKYVPDAERAAQ

>CORE_REP|Org72_Gene686#

MGVSIEVNGLTKSFGSSRIWEDVTLTIPAGEVSVLLGPSGTGKSVFLKSLIGLLRPERGSIIIDGTDIIECSAKELYEIRTLFGVLFQDGALFGSMNLYDNTAFPLREHTKKKESEIRDIVMEKLALVGLGGDEKKFPGEISGGMRKRAGLARALVLDPQIILCDEPDSGLDPVRTAYLSQLIMDINAQIDATILIVTHNINIARTVPDNMGMLFRKHLVMFGPREVLLTSDEPVVRQFLNGRRIGPIGMSEEKDEATMAEEQALLDAGHHAGGVEEIEGVPPQISATPGMPERKAVARRQARVREMLHTLPKKAQAAILDDLEGTHKYAVHEIGQ

>CORE_REP|Org327_Gene4095#

MTAPPVHDRAHHPVRDVIVIGSGPAGYTAALYAARAQLAPLVFEGTSFGGALMTTTDVENYPGFRNGITGPELMDEMREQALRFGADLRMEDVESVSLHGPLKSVVTADGQTHRARAVILAMGAAARYLQVPGEQELLGRGVSSCATCDGFFFRDQDIAVIGGGDSAMEEATFLTRFARSVTLVHRRDEFRASKIMLDRARNNDKIRFLTNHTVVAVDGDTTVTGLRVRDTNTGAETTLPVTGVFVAIGHEPRSGLVREAIDVDPDGYVLVQGRTTSTSLPGVFAAGDLVDRTYRQAVTAAGSGCAAAIDAERWLAEHAATGEADSTDALIGAQR

>CORE_REP|Org54_Gene3628#

MRLLVTGGAGFIGTNFVHSAVREHPDDAVTVLDALTYAGRRESLADVEDAIRLVQGDITDAELVSQLVAESDAVVHFAAESHVDNALDNPEPFLHTNVIGTFTILEAVRRHGVRLHHISTDEVYGDLELDDRARFTESTPYNPSSPYSATKAGADMLVRAWVRSYGVRATISNCSNNYGPYQHVEKFIPRQITNVLTGRRPKLYGAGANVRDWIHVDDHNSAVRRILDRGRIGRTYLISSEGERDNLTVLRTLLRLMDRDPDDFDHVTDRVGHDLRYAIDPSTLYDELCWAPKHTDFEEGLRTTIDWYRDNESWWRPLKDATEARYQERGQ

>CORE_REP|Org22_Gene2668#

MGGLTISDLVVEYSSGGYAVRPIDGLSLDVAPGSLVILLGPSGCGKTTLLSCLGGILRPKSGSIKFDDVDITTLEGAALAKYRRDKVGIVFQAFNLVSSLTALENVMVPLRAAGVSRAAARKRAEDLLIRVNLGERMKHRPGDMSGGQQQRVAVARAIALDPQLILADEPTAHLDFIQVEEVLRLIRSLAQGDRVVVVATHDSRMLPLADRVLELMPAQVSPNQPPETVHVKAGEVLFEQSTLGDLIYVVSEGEFEIVRELADGGEELVKTAAPGDYFGEIGVLFHLPRSATVRARSDATAVGYTAQAFRERLGVTRVADLIEHRELASE

>CORE_REP|Org155_Gene1578#

MNAHTSVGPLDRAARVYIAGHRGLVGSALLRTFAGAGFTNLLVRSRAELDLTDRAATFDFVLESRPQVVIDAAARVGGILANDTYPADFLSENLQIQVNLLDAAVAARVPRLLFLGSSCIYPKLAPQPIPESALLTGPLEPTNDAYAIAKIAGILAVQAVRRQHGLPWISAMPTNLYGPGDNFSPSGSHLLPALIRRYDEAKASGAPNVTNWGTGTPRRELLHVDDLASACLYLLEHFDGPTHVNVGTGIDHTIGEIAEMVASAVGYSGETRWDPSKPEGTPRKLLDVSVLREAGWRPSIALRDGIEATVAWYREHAGTVRQ

>CORE_REP|Org321_Gene1461#

MPSLDNTADEKPAIDPILLKVLDAVPFRLSIDDGIEAVRQRLRDLPRQPVHPELRVVDLAIDGPAGPIGTRIYWPPTCPDQAEAPVVLYFHGGGFVMGDLDTHDGTCRQHAVGADAIVVSVDYRLAPEHPYPAAIEDAWAATRWVAEHGRQVGADLGRIAVAGDSAGGTIAAVIAQRARDMGGPPIVFQLLWYPSTLWDQSLPSLAENADAPILDVKAIAAFSRWYAGEIDLHNPPAPMAPGRAENLADLPPAYIAVAGYDPLRDDGIRYGELLAAAGVPVEVHNAQTLVHGYVGYAGVVPAATEATNRGLVALRVVLHG

>CORE_REP|Org235_Gene120#

MKVWITGAGGMMGSHLAEMLLAAGHDVYATYCRPTIDPSDLQFNGAEVDITDWCSVYDSIATFRPDAVFHLAAQSYPAVSWARPVETLTTNMVGTAIVFEALRRVRPHAKIIVAGSSAEYGFVDPSEVPINERRELRPLHPYGVSKAATDMLAYQYHKSYGMHTVVARIFNCTGPRKVGDALSDFVRRCTWLEHHPEQSAIRVGNLKTKRTIVDVRDLNRALMLMLDKGEAGADYNVGGSIAYEMGDVLKQVIAACKRDDIVPEVDPALLRPTDEKIIYGDCSKLAAITGWQQEICLTQTIADMFDYWRSKSESALMV

>CORE_REP|Org117_Gene3735#

MLLAIDVRNTHTVVGLLSGMKEHAKVVQQWRIRTESEVTADELALTIDGLIGEDSERLTGTAALSTVPSVLHEVRIMLDQYWPSVPHVLIEPGVRTGIPLLVDNPKEVGADRIVNCLAAYDRFRKAAIVVDFGSSICVDVVSAKGEFLGGAIAPGVQVSSDAAAARSAALRRVELARPRSVVGKNTVECMQAGAVXGFAGLVDGLVGRIREDVSGFSVDHDVAIVATGHTAPLLLPELHTVDHYDQHLTLQGLRLVFERNLEVQRGRLKTARXRRCRHRVWVPGRPPPTGSTPPTSVGVRSFKLARRECR

>CORE_REP|Org170_Gene1533#

MDRCCQRATAFACALRPTKLIDYEEMFRGAMQARAMVANPDQWADSDRDQVNTRHYLSTSMRVALDRGEFFLVYQPIIRLADNRIIGAEALLRWEHPTLGTLLPGRFIDRAENNGLMVPLTAFVLEQACRHVRSWRDHSTDPQPFVSVNVSASTICDPGFLVLVEGVLGETGLPAHALQLELAEDARLSRDEKAVTRLQELSALGVGIAIDDFGIGFSSLAYLPRLPVDVVKLGGKFIECLDGDIQARLANEQITRAMIDLGDKLGITVTAKLVETPSQAARLRAFGCKAAQGWHFAKALPVDFFRE

>CORE_REP|Org311_Gene1042#

MSRPEVLTPFTAIVPAAGLGTRFLPATKTVPKELLPVVDTPGIELVAAEAAAAGAERLVIVTSEGKDGVVAHFVEDLVLEGTLEARGKIAMLAKVRRAPALIKVESVVQAEPLGLGHAIGCVEPTLSPDEDAVAVLLPDDLVLPTGVLETMSKVRASRGGTVLCAIEVAREEISAYGVFDVEPVPDGDYTDDPNVLKVRGMVEKPKAETAPSRYAAAGRYVLDRAIFDALRRIDRGAGGEVQLTDAIALLIAEGHPVHVVVHQGSRHDLGNPGGYLKAAVDFALDRDDYGPDLRRWLVARLGLTEQ

>CORE_REP|Org171_Gene3412#

MRSVQAPKGHLGSGKALHLMVNDLTPHFEDVQAHYDLSDDFFRLFLDPTQTYSCAHFEREDMTLEEAQIAKIDLALGKLGLQPGMTLLDIGCGWGATMRRAIAQYDVNVVGLTLSKNQAAHVQKSFDEMDTPRDRRVLLAGWEQFNEPVDRIVSIGAFEHFGHDRHADFFARAHKILPPDGVLLLHTITGLTRQQMVDHGLPLTLWLARFLKFIATEIFPGGQPPTIEMVEDQSAKTGFTLTRRQSLQPHYARTLDLWAEALQEHKSEAIAIQSEEVYERYMKYLTGCAKLFRVGYIDVNQFTLAK

>CORE_REP|Org380_Gene1860#

MDQQSTRTDITVNVDGFWMLQALLDIRHVAPELRCRPYVSTDSNDWLNEHPGMAVMREQGIVVNDAVNEQVAARMKVLAAPDLEVVALLSRGKLLYGVIDDENQPPGSRDIPDNEFRVVLARRGQHWVSAVRVGNDITVDDVTVSDSASIAALVMDGLESIHHADPAAINAVNVPMEEMLEATKSWQESGFNVFSGGDLRRMGISAATVAALGQALSDPAAEVAVYARQYRDDAKGPSASVLSLKDGSGGRIALYQQARTAGSGEAWLAICPATPQLVQVGVKTVLDTLPYGEWKTHSRV

>CORE_REP|Org369_Gene983#

MPCIHSRHTPRFREQSQEAAASLLPFRPTAGKEGAMILDMFRLDDKVAVITGGGRGLGAAIALAFAQAGADVLIASRTSSELDAVAEQIRAAGRRAHTVAADLAHPEVTAQLAGQAVGAFGKLDIVVNNVGGTMPNTLLSTSTKDLADAFAFNVGTAHALTVAAVPLMLEHSGGGSVINISSTMGRLAARGFAAYGTAKAALAHYTRLAALDLCPRVRVNAIAPGSILTSALEVVAANDELRAPMEQATPLRRLGDPVDIAAAAVYLASPAGSFLTGKTLEVDGGLTFPNLDLPIPDL

>CORE_REP|Org160_Gene816#

MRPALSDYQHVASGKVREIYRVDDEHLLLVASDRISAYDYVLDSTIPDKGRVLTAMSAFFFGLVDAPNHLAGPPDDPRIPDEVLGRALVVRRLEMLPVECVARGYLTGSGLLDYQATGKVCGIALPPGLVEASRFATPLFTPATKAALGDHDENISFDRVVEMVGALRANQLRDRTLQTYVQAADHALTRGIIIADTKFEFGIDRHGNLLLADEIFTPDSSRYWPADDYRAGVVQTSFDKQFVRSWLTGSESGWDRGSDRPPPPLPEHIVEATRARYINAYERISELKFDDWIGPGA

>CORE_REP|Org375_Gene312#

MDATPNAVELTVDNAWFIAETIGAGTFPWVLAITMPYSDAAQRGAFVDRQRDELTRMGLLSPQGVINPAVADWIKVVCFPDRWLDLRYVGPASADGACELLRGIVALRTGTGKTSNKTGNGVVALRNAQLVTFTAMDIDDPRALVPILGVGLAHRPPARFDEFSLPTRVGARADERLRSGVPLGEVVDYLGIPASARPVVESVFSGPRSYVEIVAGCNRDGRHTTTEVGLSIVDTSAGRVLVSPSRAFDGEWVSTFSPGTPFAIAVAIQTLTACLPDGQWFPGQRVSRDFSTQSS

>CORE_REP|Org88_Gene3767#

MQVMGVLNVTDDSFSDGGCYLDLDDAVKHGLAMAAAGAGIVDVGGESSRPGATRVDPAVETSRVIPVVKELAAQGITVSIDTMRADVARAALQNGAQMVNDVSGGRADPAMGPLLAEADVPWVLMHWRAVSADTPHVPVRYGNVVAEVRADLLASVADAVAAGVDPARLVLDPGLGFAKTAQHNWAILHALPELVATGIPVLVGASRKRFLGALLAGPDGVMRPTDGRDTATAVISALAALHGAWGVRVHDVRASVDAIKVVEAWMGAERIERDG

>CORE_REP|Org143_Gene3941#

MSDPHHPHIQTHNAWVEFPIFDAKSRSLKKAVLGKAGGTIGRNNSNVVVIEALRDITMELNLGDRVGLVGHNGAGKSTLLRLLSGIYEPTRGWAKVTGRVAPVFDLGIGMDPEISGYENIIIRGLFLGQTRKQMQAKVDEIAEFTELGEYLSMPLRTYSTGMRVRLAMGVVTSIDPEILLLDEGIGAVDADFLRKAQSRLQNLVERSGILVFASHSNEFLARLCKTAIWIDHGVIRLAGGIEEVVRAYEGEDAARHVREVLAETQADRQNVQG

>CORE_REP|Org165_Gene3339#

MTDTRVLAVANQKGGVAKTTTVASLGAAMVEKGRRVLLVDLDPQGCLTFSLGQDPDKLPVSVHEVLLGEVEPNAVLVTTMEGMTLLPANIDLAGAEAMLLMRAGREYALKRALAKFSDRFDVVIIDCPPSLGVLTLNGLTAADEAIVPLQCEMLAHRGVGQFLRTVADVQQITNPNLRLLGALPTLYDSRTTHTRDVLLDVADRYDLQVLAPPIPRTVRFAEASASGSSVMAGRKNKGAVAYRELAQALLKHWKTGRPLPTFTVDL

>CORE_REP|Org50_Gene1540#

MTILEIKDLHVSVENPAEADHEIPILRGVDLTVKSGETHALMGPNGSGKSTLSYAIAGHPKYHVTSGTITLDGADVLAMSIDERARAGLFLAMQYPVEVPGVSMSNFLRSAATAIRGEPPKLRHWVKEVKAAMAALDIDPAFAERSVNEGFSGGEKKRHEILQLELLKPKIAILDETDSGLDVDALRVVSEGVNRYAESQHGGILLITHYTRILRYIHPEYVHVFVGGRIVESGGSELADELDQNGYVRFSPASGRYPHQPAPTGA

>CORE_REP|Org333_Gene1319#

MELLGGPRVGNTESQLCVADGDDLPTYCSANSEDLNITTITTLSPTSMSHPQQVRDDQWVEPSDQLQGTAVFDATGDKATMPSWDELVRQHADRVYRLAYRLSGNQHDAEDLTQETFIRVFRSVQNYQPGTFEGWLHRITTNLFLDMVRRRARIRMEALPEDYDRVPADEPNPEQIYHDARLGPDLQAALASLPPEFRAAVVLCDIEGLSYEEIGATLGVKLGTVRSRIHRGRQALRDYLAAHPEHGECAVHVNPVR

>CORE_REP|Org289_Gene1816#

MVSRARGNGSAMRLARRARXILRXNGIEVSRYFAELDWERNFLRQLQSHRVSAVLDVGANSGQYARGLRGAGXXXRIVSFEXXPGPFAVLQRSASTDPLWECRRCALGDVDGTISINVAGNEGASSSVLPMLKRHQDAFPPANYVGAQRVPIHRLDSVAADVLRPNDIAFLKIDVQGFEKQVIAGGDSTVHDRCVGMQLELSFQPLYEGGMLIREALDLVDSLGFTLSGLQPGFTDPRNGRMLQADGIFFRGSD

>CORE_REP|Org365_Gene857#

MELLLLTSELYPDPVLPALSLLPHTVRTAPAEASSLLEAGNADAVLVDARNDLSSGRGLCRLLSSTGRSIPVLAVVSEGGLVAVSADWGLDEILLLSTGPAEIDARLRLVVGRRGDLADQESLGKVSLGELVIDEGTYTARLRGRPLDLTYKEFELLKYLAQHAGRVFTRAQLLHEVWGYDFFGGTRTVDVHVRRLRAKLGPEHEALIGTVRNVGYKAVRPARGRPPAADPDDEDADPGRDGMQEPLVDPLRSQ

>CORE_REP|Org277_Gene2430#

MLTAMRGDIRAARERDPAAPTALEVIFCYPGVHAVWGHRLAHWLWQRGARLLARAAAEFTRILTGVDIHPGAVIGARVFIDHATGVVIGETAEVGDDVTIYHGVTLGGSGMVGGKRHPTVGDRVIIGAGAKVLGPIKIGEDSRIGANAVVVKPVPPSAVVVGVPGQVIGQSQPSPGGPFDWRLPDLVEPASIRCSPGWPGWRPSAAARKQQESSGHPKPGYGTARTSRSEAIPGRRQCLLRRRPPTRIIGC

>CORE_REP|Org427_Gene2704#

MSGHSKWATTKHKKAVVDARRGKMFARLIKNIEVAARVGGGDPAGNPTLYDAIQKAKKSSVPNENIERARKRGAGEEAGGADWQTIMYEGYAPNGVAVLIECLTDNRNRAASEVRVAMTRNGGTMADPGSVSYLFSRKGVVTLEKNGLTEDDVLAAVLEAGAEDVNDLGDSFEVISEPAELVAVRSALQDAGIDYESAEASFQPSVSVPVDLDGARKVFKLVDALEDSDDVQNVWTNVDVSDEVLAALDDE

>CORE_REP|Org365_Gene512#

MTCADDDAERSDEVGAPPVALMTSVLIVEDEESLADPLAFLLRKEGFEATVVTDGPAALAEFDRAGADIVLLDLMLPGMSGTDVCKQLRARSSVPVIMVTARDSEIDKVVGLELGADDYVTKPYSARELIARIRAVLRRGGDDDSEMSDGVLESGPVRMDVERHVVSVNGDTITLPLKEFDLLEYLMRNSGRVLTRGQLIDRVWGADYVGDTKTLDVHVKRLRSKIEADPANPVHLVTVRGLGYKLEG

>CORE_REP|Org266_Gene1023#

MNRQPIVQLSNLSWTFREGETRRQVLDHITFDFEPGEFVALLGQSGSGKSTLLNLISGIEKPTTGDVTINGFAITQKTERDRTLFRRDQIGIVFQFFNLIPTLTVLENITLPQELAGVSQRKAAVVARDLLEKVGMADRERTFPDKLSGGEQQRVAISRALAHNPMLVLADEPTGNLDSDTGDKVLDVLLDLTRQAGKTLIMATHSPSMTQHADRVVNLQGGRLIPAVNRENQTDQPASTILLPTSYE

>CORE_REP|Org437_Gene945#

MGGMDTGVTSPRVLVVDDDSDVLASLERGLRLSGFEVATAVDGAEALRSATENRPDAIVLDINMPVLDGVSVVTALRAMDNDVPVCVLSARSSVDDRVAGLEAGADDYLVKPFVLAELVARVKALLRRRGSTATSSSETITVGPLEVDIPGRRARVNGVDVDLTKREFDLLAVLAEHKTAVLSRAQLLELVWGYDFAADTNVVDVFIGYLRRKLEAGGGPRLLHTVRGVGFVLRMQ

>CORE_REP|Org300_Gene3196#

MIITLDHVTKQYKSSARPALDDINVKIDKGEFVFLIGPSGSGKSTFMRLLLAAETPTSGDVRVSKFHVNKLRGRHVPKLRQVIGCVFQDFRLLQQKTVYDNVAFALEVIGKRTDAINRVVPEVLETVGLSGKANRLPDELSGGEQQRVAIARAFVNRPLVLLADEPTGNLDPETSRDIMDLLERINRTGTTVLMATHDHHIVDSMRQRVVELSLGRLVRDEQRGVYGMDR

>CORE_REP|Org90_Gene1782#

MSRLSAAVVAIDGPAGTGKSSVSRRLARELGARFLDTGAMYRIVTLAVLRAGADPSDIAAVETIASTVQMSLGYDPDGDSCYLAGEDVSVEIRGDAVTRAVSAVSSVPAVRTRLVELQRTMAEGPGSIVVEGRDIGTVVFPDAPVKIFLTASAETRARRRNAQNVAAGLADDYDGVLADVRRRDHLDSTRAVSPLQAAGDAVIVDTSDMTEAEVVAHLLELVTRRSEAVR

>CORE_REP|Org441_Gene488#

MNELVDTTEMYLRTIYDLEEEGVTPLRARIAERLDQSGPTVSQTVSRMERDGLLRVAGDRHLELTEKGRALAIAVMRKHRLAERLLVDVIGLPWEEVHAEACRWEHVMSEDVERRLVKVLNNPTTSPFGNPIPGLVELGVGPEPGADDANLVRLTELPAGSPVAVVVRQLTEHVQGDIDLITRLKDAGVVPNARVTVETTPGGGVTIVIPGHENVTLPHEMAHAVKVEKV

>CORE_REP|Org268_Gene2865#

MTVGTLVASVLPATVFEDLAYAELYSDPPGLTPLPEEAPLIARSVAKRRNEFITVRHCARIALDQLGVPPAPILKGDKGEPCWPDGVVGSLTHCAGYRGAVVGRRDAVRSVGIDAEPHDVLPNGVLDAISLPAERADMPRTMPAALHWDRILFCAKEATYKAWFPLTKRWLGFEDAHITFETDSTGWTGRFVSRILIDGSTLSGPPLTTLRGRWSVERGLVLTAIVL

>CORE_REP|Org184_Gene3246#

MVKVFLVDDHEVVRRGLVDLLGADPELDVVGEAGSVAEAMARVPAARPDVAVLDVRLPDGNGIELCRDLLSRMPDLRCLILTSYTSDEAMLDAILAGASGYVVKDIKGMELARAVKDVGAGRSLLDNRAAAALMAKLRGAAEKQDPLSGLTDQERTLLGLLSEGLTNKQIADRMFLAEKTVKNYVSRLLAKLGMERRTQAAVFATELKRSRPPGDGP

>CORE_REP|Org53_Gene366#

MTISFSSSNLRDDATSGNGDYRLDKLPETTPSTSVFDRADVTYRQFTELHGQARDTRREAHVVELESKTGERARCAPMHALEQLADYGFAWRDIARVVGVSVPAITKWRKGAGVTGENRLKIARLLALIDMLSDRFIGEPASWLEMPIQAGVGITRMDLLERGRYDLVLALASTHTGDGTVEYVLNETDKDWRETVVDNAFESYTAEDGVISIRPKR

>CORE_REP|Org289_Gene3545#

MLWHNSACMWGKNMTECFLSDQEIRKLNRDLRILIAANGTLTRVLNIVADDEVIVQIVKQRIHDVSPKLSEFEQLGQVGVGRVLQRYIILKGRNSEHLFVAAESLIAIDRLPAAIITRLTQTNDPLGEVMAASHIETFKEEAKVWVGDLPGWLALHGYQNSRKRAVARRYRVISGGQPIMVVTEHFLRSVFRDAPHEEPDRWQFSNAITLAR

>CORE_REP|Org241_Gene2497#

MPLFSFEGRSPRIDPTAFVAPTATLIGDVTIEAGASVWFNAVLRGDYAPVVVREGANVQDGAVLHAPPGIPVDIGPGATVAHLCVIHGVHVGSEALIANHATVLDGAVIGARCMIAAGALVVAGTQIPAGMLVTGAPAKVKGPIEGTGAEMWVNVNPQAYRDLAARHLAGLEPMQASLRVKPSAVLTSGCKSRSRRRRRRCPPPHQLGRRVR

>CORE_REP|Org276_Gene3094#

MEAFHTHSGIGVPLRRSNVDTDQIIPAVFLKRVTRTGFEDGLFAGWRSDPAFVLNLSPFDRGSVLVAGPDFGTGSSREHAVWALMDYGFRVVISSRFGDIFRGNAGKAGLLAAEVAQDDVELLWKLIEQSPGLEITANLQDRIITAATVVLPFKIDDHSAWRLLEGLDDIALTLRKLDEIEAFEGACAYWKPRTLPAP

>CORE_REP|Org426_Gene749#

MSRIGKQPIPVPAGVDVTIEGQSISVKGPKGTLGLTVAEPIKVARNDDGAIVVTRPDDERRNRSLHGLSRTLVSNLVTGVTQGYTTKMEIFGVGYRVQLKGSNLEFALGYSHPVVIEAPEGITFAVQAPTKFTVSGIDKQKVGQIAANIRRLRRPDPYKGKGVRYEGEQIRRKVGKTGK

>CORE_REP|Org367_Gene2648#

MAPKAVLVGLPGSGKSTIGRRLAKALGVGLLDTDVAIEQRTGRSIADIFATDGEQEFRRIEEDVVRAALADHDGVLSLGGGAVTSPGVRAALAGHTVVYLEISAAEGVRRTGGNTVRPLLAGPDRAEKYRALMAKRAPLYRRVATMRVDTNRRNPGAVVRHILSRLQVPSPSEAAT

>CORE_REP|Org347_Gene1341#

MTVTDDYLANNVDYASGFKGPLPMPPSKHIAIVACMDARLDVYRMLGIKEGEAHVIRNAGCVVTDDVIRSLAISQRLLGTREIILLHHTDCGMLTFTDDDFKRAIQDETGIRPTWSPESYPDAVEDVRQSLRRIEVNPFVTKHTSLRGFVFDVATGKLNEVTP

>CORE_REP|Org58_Gene3054#

MTGAVCPGSFDPVTLGHVDIFERAAAQFDEVVVAILVNPAKTGMFDLDERIAMVKESTTHLPNLRVQVGHGLVVDFVRSCGMTAIVKGLRTGTDFEYELQMAQMNKHIAGVDTFFVATAPRYSFVSSSLAKEVAMLGGDVSELLPEPVNRRLRDRLNTERT

>CORE_REP|Org199_Gene3578#

MTETTPAPQTPAAPAGPAQSFVLERPIQTVGRRKEAVVRVRLVPGTGKFDLNGRSLEDYFPNKVHQQLIKAPLVTVDRVESFDIFAHLGGGGPSGQAGALRLGIARALILVSPEDRPALKKAGFLTRDPRATERKKYGLKKARKAPQYSKR

>CORE_REP|Org106_Gene3755#

MLRTMLKSKIHRATVTCADLHYVGSVTIDADLMDAADLLEGEQVTIVDIDNGARLVTYAITGERGSGVIGINGAAAHLVHPGDLVILIAYATMDDARARTYQPRIVFVDAYNKPIDMGHDPAFVPENAGELLDPRLGVG

>CORE_REP|Org390_Gene4044#

MTDSEKSATIKVTDASFATDVLSSNKPVLVDFWATWCGPCKMVAPVLEEIATERATDLTVAKLDVDTNPETARNFQVVSIPTLILFKDGQPVKRIVGAKGKAALLRELSDVVPNLN

>CORE_REP|Org110_Gene1928#

MRLTPHEQERLLLSYAAELARRRRARGLRLNHPEAIAVIADHILEGARDGRTVAELMASGREVLGRDDVMEGVPEMLAEVQVEATFPDGTKLVTVHQPIA
