# Supplementary material for: Exploring optimal drug targets through subtractive proteomics analysis and pangenomic insights for tailored drug design in tuberculosis
Source: Sci Rep. 2024 May 13;14:10904. doi: 10.1038/s41598-024-61752-6 (PMC11091173; doi:10.1038/s41598-024-61752-6)
Supplement: Supplementary file 2 — Supplementary Information 2. [file 41598_2024_61752_MOESM2_ESM.docx]

**File S2:** Protein targets found to have appropriate physiochemical parameters provided in Fasta format below:

>CORE_REP|Org27_Gene1697#

MSFSERDSVADRLIVKGAREHNLRSVDLDLPRDALIVFTGLSGSGKSSLAFDTIFAEGQRRYVESLSAYARQFLGQMDKPDVDFIEGLSPAVSIDQKSTNRNPRSTVGTITEVYDYLRLLYARAGTPHCPTCGERVARQTPQQIVDQVLAMPEGTRFLVLAPVVRTRKGEFADLFDKLNAQGYSRVRVDGVVHPLTDPPKLKKQEKHDIEVVVDRLTVKAAAKRRLTDSVETALNLADGIVVLEFVDHELGAPHREQRFSEKLACPNGHALAVDDLEPRSFSFNSPYGACPECSGLGIRKEVDPELVVPDPDRTLAQGAVAPWSNGHTAEYFTRMMAGLGEALGFDVDTPWRKLPAKARKAILEGADEQVHVRYRNRYGRTRSYYADFEGVLAFLQRKMSQTESEQMKERYEGFMRDVPCPVCAGTRLKPEILAVTLAGESKGEHGAKSIAEVCELSIADCADFLNALTLGPREQAIAGQVLKEIRSRLGFLLDVGLEYLSLSRAAATLSGGEAQRIRLATQIGSGLVGVLYVLDEPSIGLHQRDNRRLIETLTRLRDLGNTLIVVEHDEDTIEHADWIVDIGPGAGEHGGRIVHSGPYDELLRNKDSITGAYLSGRESIEIPAIRRSVDPRRQLTVVGAREHNLRGIDVSFPLGVLTSVTGVSGSGKSTLVNDILAAVLANRLNGARQVPGRHTRVTGLDYLDKLVRVDQSPIGRTPRSNPATYTGVFDKIRTLFAATTEAKVRGYQPGRFSFNVKGGRCEACTGDGTIKIEMNFLPDVYVPCEVCQGARYNRETLEVHYKGKTVSEVLDMSIEEAAEFFEPIAGVHRYLRTLVDVGLGYVRLGQPAPTLSGGEAQRVKLASELQKRSTGRTVYILDEPTTGLHFDDIRKLLNVINGLVDKGNTVIVIEHNLDVIKTSDWIIDLGPEGGAGGGTVVAQGTPEDVAAVPASYTGKFLAEVVGGGASAATSRSNRRRNVSA

>CORE_REP|Org163_Gene1928#

MTSSRAVNVHGCPRIAACRCTDTHPRGRPAFAYRWFVPKTTRAQPGRLSSRFWRLLGASTEKNRSRSLADVTASAEYDKEAADLSDEKLRKAAGLLNLDDLAESADIPQFLAIAREAAERRTGLRPFDVQLLGALRMLAGDVIEMATGEGKTLAGAIAAAGYALAGRHVHVVTINDYLARRDAEWMGPLLDAMGLTVGWITADSTPDERRTAYDRDVTYASVNEIGFDVLRDQLVTDVNDLVSPNPDVALIDEADSVLVDEALVPLVLAGTTHRETPRLEIIRLVAELVGDKDADEYFATDSDNRNVHLTEHGARKVEKALGGIDLYSEEHVGTTLTEVNVALHAHVLLQRDVHYIVRDDAVHLINASRGRIAQLQRWPDGLQAAVEAKEGIETTETGEVLDTITVQALINRYATMCGMTGTALAAGEQLRQFYQLGVSPIPPNKPNIREDEADRVYITTAAKNDGIVEHITEVHQRGQPVLVGTRDVAESEELHERLVRRGVPAVVLNAKNDAEEARVIAEAGKYGAVTVSTQMAGRGTDIRLGGSDEADHDRVAELGGLHVVGTGRHHTERLDNQLRGRAGRQGDPGSSVFFSSWEDDVVAANLDHNKLPMATDENGRIVSPRTGSLLDHAQRVAEGRLLDVHANTWRYNQLIAQQRAIIVERRNTLLRTVTAREELAELAPKRYEELSDKVSEERLETICRQIMLYHLDRGWADHLAYLADIRESIHLRALGRQNPLDEFHRMAVDAFASLAADAIEAAQQTFETANVLDHEPGLDLSKLARPTSTWTYMVNDNPLSDDTLSALSLPGVFR

>CORE_REP|Org172_Gene2465#

MAEDQLTAQAVAPPTEASAALEPALETPESPVETLKTSISASRRVRARLARRMTAQRSTTNPVLEPLVAVHREIYPKADLSILQRAYEVADQRHASQLRQSGDPYITHPLAVANILAELGMDTTTLVAALLHDTVEDTGYTLEALTEEFGEEVGHLVDGVTKLDRVVLGSAAEGETIRKMITAMARDPRVLVIKVADRLHNMRTMRFLPPEKQARKARETLEVIAPLAHRLGMASVKWELEDLSFAILHPKKYEEIVRLVAGRAPSRDTYLAKVRAEIVNTLTASKIKATVEGRPKHYWSIYQKMIVKGRDFDDIHDLVGVRILCDEIRDCYAAVGVVHSLWQPMAGRFKDYIAQPRYGVYQSLHTTVVGPEGKPLEVQIRTRDMHRTAEYGIAAHWRYKEAKGRNGVLHPHAAAEIDDMAWMRQLLDWQREAADPGEFLESLRYDLAVQEIFVFTPKGDVITLPTGSTPVDFAYAVHTEVGHRCIGARVNGRLVALERKLENGEVVEVFTSKAPNAGPSRDWQQFVVSPRAKTKIRQWFAKERREEALETGKDAMAREVRRGGLPLQRLVNGESMAAVARELHYADVSALYTAIGEGHVSAKHVVQRLLAELGGIDQAEEELAERSTPATMPRRPRSTDDVGVSVPGAPGVLTKLAKCCTPVPGDVIMGFVTRGGGVSVHRTDCTNAASLQQQAERIIEVLWAPSPSSVFLVAIQVEALDRHRLLSDVTRALADEKVNILSASVTTSGDRVAISRFTFEMGDPKHLGHLLNAVRNVEGVYDVYRVTSAA

>CORE_REP|Org106_Gene302#

MAGVGEGDSGGVERDDIGMVAASPVASRVNGKVDADVVGRFATCCRALGIAVYQRKRPPDLAAARSGFAALTRVAHDQCDAWTGLAAAGDQSIGVLEAASRTATTAGVLQRQVELADNALGFLYDTGLYLRFRATGPDDFHLAYAAALASTGGPEEFAKANHVVSGITERRAGWRAARWLAVVINYRAERWSDVVKLLTPMVNDPDLDEAFSHAAKITLGTALARLGMFAPALSYLEEPDGPVAVAAVDGALAKALVLRAHVDEESASEVLQDLYAAHPENEQVEQALSDTSFGIVTTTAGRIEARTDPWDPATEPGAEDFVDPAAHERKAALLHEAELQLAEFIGLDEVKRQVSRLKSSVAMELVRKQRGLTVAQRTHHLVFAGPPGTGKTTIARVVAKIYCGLGLLKRENIREVHRADLIGQHIGETEAKTNAIIDSALDGVLFLDEAYALVATGAKNDFGLVAIDTLLARMENDRDRLVVIIAGYRADLDKFLDTNEGLRSRFTRNIDFPSYTSHELVEIAHKMAEQRDSVFEQSALHDLEALFAKLAAESTPDTNGISRRSLDIAGNGRFVRNIVERSEEEREFRLDHSEHAGSGEFSDEELMTITADDVGRSVEPLLRGLGLSVRA

>CORE_REP|Org101_Gene3957#

MAYHNPFIVNGKIRFPANTNLVRHVEKWAKVRGDKLAYRFLDFSTERDGVARDILWSDFSARNRAVGARLQQVTQPGDRVAILCPQNLDYLISFFGALYSGRIAVPLFDPAEPGHVGRLHAVLDDCAPSTILTTTDSAEGVRKFIRARSAKERPRVIAVDAVPTEVAATWQQPEANEETVAYLQYTSGSTRIPSGVQITHLNLPTNVVQVLNALEGQESDRGVSWLPFFHDMGLITVLLASVLGHSFTFMTPAAFVRRPGRWIRELARKPGETGGTFSAAPNFAFEHAAVRGVPRDDEPPLDLSNVKGILNGSEPVSPASMRKFFEAFAPYGLKQTAVKPSYGLAEATLFVSTTPMDEVPTVIHVDRDELNNQRFVEVAADAPNAVAQVSAGKVGVSEWAVIVDADTASELPDGQIGEIWLHGNNLGTGYWGKEEESAQTFKNILKSRISESRAEGAPDDALWVRTGDYGTYFKDHLYIAGRIKDLVIIDGRNHYPQDLECTAQESTKALRVGYAAAFSVPANQLPQTVFDDSHAGLKFDPEDTSEQLVIVGERAAGTHKLDHQPIVDDIRAAIAVGHGVTVRDVLLVSAGTIPRTSSGKIGRRACRAAYLDGSLRSGVGSPTVFATSD

>CORE_REP|Org156_Gene3842#

MERFDGLRPARLKVGIISAGRVGTALGVALQRADHVVMACSAISHASRRRAQRRLPDTPVLPPLDVAASAELLLLAVTDSELAGLVSGLAATSAVRPQTIVAHTSGANGIGILAPLAQQGCIPLAIHPAMTFTGSDEDISRLPDTCFGITAADDVGYAIGQSLVLEMGGEPFCVREDARILYHAALAHASNHIVTVLADALEALRAALSGGTARPTNRRRPAGRDRGAHRRAAGQSGAGEHAAAGTGRAHRTGRPRRCGSGRGSSGGPRGRRRSAGPGIPDKRAADRAARTRPRGCRRGFDGMTIPAFHPGELNVYSAPGDVADVSRALRLTGRRVMLVPTMGALHEGHLALVRAAKRVPGSVVVVSIFVNPMQFGAGEDLDAYPRTPDDDLAQLRAEGVEIAFTPTTAAMYPDGLRTTVQPGPLAAELEGGPRPTHFAGVLTVVLKLLQIVRPDRVFFGEKDYQQLVLIRQLVADFNLDVAVVGVPTVREADGLAMSSRNRYLDPAQRAAAVALSAALTAAAHAATAGAQAALDAARAVLDAAPGVAVDYLELRDIGLGPMPLNGSGRLLVAARLGTTRLLDNIAIEIGTFAGTDRPDGYRAILESHWRN

>CORE_REP|Org395_Gene1984#

MFWVGGPCLMPASSAARCAARIVGGRCLMPASSAARYAARIVGGRCLMPASSAARCAARIVGGPRLYGMQRIIGTEVEYGISSPSDPTANPILTSTQAVLAYAAAAGIQRAKRTRWDYEVESPLRDARGFDLSRSAGPPPVVDADEVGAANMILTNGARLYVDHAHPEYSAPECTDPLDAVIWDKAGERVMEAAARHVASVPGAAKLQLYKNNVDGKGASYGSHENYLMSRQTPFSAIITGLTPFLVSRQVVTGSGRVGIGPSGDEPGFQLSQRSDYIEVEVGLETTLKRGIINTRDEPHADADRYRRLHVIIGDANLAETSTYLKLGTTALVLDLIEEGPAHAIDLTDLALARPVHAVHAISRDPSLRATVALADGRELTGLALQRIYLDRVAKLVDSRDPDPRAADIVETWAHVLDQLERDPMDCAELLDWPAKLRLLDGFRQRENLSWSAPRLHLVDLQYSDVRLDKGLYNRLVARGSMKRLVTEHQVLSAVENPPTDTRAYFRGECLRRFGADIAAASWDSVIFDLGGDSLVRIPTLEPLRGSKAHVGALLDSVDSAVELVEQLTAEPR

>CORE_REP|Org322_Gene4048#

MTDRLASLFESAVSMLPMSEARSLDLFTEITNYDESACDAWIGRIRCGDTDRVTLFRAWYSRRNFGQLSGSVQISMSTLNARIAIGGLYGDITYPVTSPLAITMGFAACEAAQGNYADAMEALEAAPVAGSEHLVAWMKAVVYGAAERWTDVIDQVKSAGKWPDKFLAGAAGVAHGVAAANLALFTEAERRLTEANDSPAGEACARAIAWYLAMARRSQGNESAAVALLEWLQTTHPEPKVAAALKDPSYRLKTTTAEQIASRADPWDPGSVVTDNSGRERLLAEAQAELDRQIGLTRVKNQIERYRAATLMARVRAAKGMKVAQPSKHMIFTGPPGTGKTTIARVVANILAGLGVIAEPKLVETSRKDFVAEYEGQSAVKTAKTIDQALGGVLFIDEAYALVQERDGRTDPFGQEALDTLLARMENDRDRLVVIIAGYSSDIDRLLETNEGLRSRFATRIEFDTYSPEELLEIANVIAAADDSALTAEAAENFLQAAKQLEQRMLRGRRALDVAGNGRYARQLVEASEQCRDMRLAQVLDIDTLDEDRLREINGSDMAEAIAAVHAHLNMRE

>CORE_REP|Org179_Gene4007#

MADPGGFLKYTHRKLPKRRPVPLRLRDWREVYEEFDNESLRQQATRCMDCGIPFCHNGCPLGNLIPEWNDLVRRGRWRDAIERLHATNNFPDFTGRLCPAPCEPACVLGINQDPVTIKQIELEIIDKAFDEGWVQPRPPRKLTGQTVAVVGSGPAGLAAAQQLTRAGHTVTVFEREDRIGGLLRYGIPEFKMEKRHLDRRLDQMRSEGTEFRPGVNVGVDISAEKLRADFDAVVLAGGATAWRELPIPGRELEGVHQAMEFLPWANRVQEGDDVLDEDGQPPITAKGKKVVIIGGGDTGADCLGTVHRQGAIAVHQFEIMPRPPDARAESTPWPTYPLMYRVSAAHEEGGERVFSVNTEAFVGTDGRVSALRAHEVTMLDGKFVKVEGSDFELEADLVLLAMGFVGPERAGLLTDLGVKFTERGNVARGDDFDTSVPGVFVAGDMGRGQSLIVWAIAEGRAAAAAVDRYLMGSSALPAPVKPTAAPLQ

>CORE_REP|Org59_Gene3250#

MDFALLPPEVNSARMYTGPGAGSLLAAAGGWDSLAAELATTAEAYGSVLSGLAALHWRGPAAESMAVTAAPYIGWLYTTAEKTQQTAIQARAAALAFEQAYAMTLPPPVVAANRIQLLALIATNFFGQNTAAIAATEAQYAEMWAQDAAAMYGYATASAAAALLTPFSPPRQTTNPAGLTAQAAAVSQATDPLSLLIETVTQALQALTIPSFIPEDFTFLDAIFAGYATVGVTQDVESFVAGTIGAESNLGLLNVGDENPAEVTPGDFGIGELVSATSPGGGVSASGAGGAASVGNTVLASVGRANSIGQLSVPPSWAAPSTRPVSALSPAGLTTLPGTDVAEHGMPRCTGGASGSRASLRRPTSIRGSAHGDGPPTRGRVTRRLTDRRPVGRKRPIVRILRRVHHRKYLTDGRPLVDVDGHALIHGVLIDRGPSDDPGDGDILLAACVRSRADSGHQERAAGQISPRAARDKPRSKKYR

>CORE_REP|Org41_Gene2297#

MNWTVDIPIDQLPSLPPLPTDLRTRLDAALAKPAAQQPTWPADQALAMRTVLESVPPVTVPSEIVRLQEQLAQVAKGEAFLLQGGDCAETFMDNTEPHIRGNVRALLQMAVVLTYGASMPVVKVARIAGQYAKPRSADIDALGLRSYRGDMINGFAPDAAAREHDPSRLVRAYANASAAMNLVRALTSSGLASLHLVHDWNREFVRTSPAGARYEALATEIDRGLRFMSACGVADRNLQTAEIYASHEALVLDYERAMLRLSDGEDGEPQLFDLSAHTVWIGERTRQIDGAHIAFAQVIANPVGVKLGPNMTPELAVEYVERLDPHNKPGRLTLVSRMGNHKVRDLLPPIVEKVQATGHQVIWQCDPMHGNTHESSTGFKTRHFDRIVDEVQGFFEVHRALGTHPGGIHVEITGENVTECLGGAQDISETDLAGRYETACDPRLNTQQSLELAFLVAEMLRD

>CORE_REP|Org32_Gene865#

MTALDWRSALTADEQRSVRALVTATTAVDGVAPVGEQVLRELGQQRTEHLLVAGSRPGGPIIGYLNLSPPRGAGGAMAELVVHPQSRRRGIGTAMARAALAKTAGRNQFWAHGTLDPARATASALGLVGVRELIQMRRPLRDIPEPTIPDGVVIRTYAGTSDDAELLRVNNAAFAGHPEQGGWTAVQLAERRGEAWFDPDGLILAFGDSPRERPGRLLGFHWTKVHPDHPGLGEVYIGPSGCGKTTVLRTLNRMHEVIPGARVEGAVLLDDQDIYAPGIDPVGVRRAIGMVFQRPNPFPAMSIRNNVVAGLKLQGVRNRKVLDDTAESSLRGANLWDEVKDRLDKPGGGLSGGQQQRLCIARAIAVQPDVLLMDEPCSSLDPISTMAIEDLISELKQQYTIVIVTHNMQQAARVSDQTAFFNLEAVGKPGRLVEIASTEKIFSNPNQKATEDYISGRFG

>CORE_REP|Org342_Gene2191#

MGIETEFGVTCTFHGHRRLSPDEVARYLFRRVVSWGRSSNVFLRNGARLYLDVGSHPEYATAECDSLVQLVTHDRAGEWVLEDLLVDAEQRLADEGIGGDIYLFKNNTDSAGNSYGCHENYLIVRAGEFSRISDVLLPFLVTRQLICGAGKVLQTPKAATYCLSQRAEHIWEGVSSATTRSRPIINTRDEPHADAEKYRRLHVIVGDSNMSETTTMLKVGTAALVLEMIESGVAFRDFSLDNPIRAIREVSHDVTGRRPVRLAGGRQASALDIQREYYTRAVEHLQTREPNAQIEQVVDLWGRQLDAVESQDFAKVDTEIDWVIKRKLFQRYQDRYDMELSHPKIAQLDLAYHDIKRGRGIFDLLQRKGLAARVTTDEEIAEAVDQPPQTTRARLRGEFISAAQEAGRDFTVDWVHLKLNDQAQRTVLCKDPFRAVDERVKRLIASM

>CORE_REP|Org427_Gene486#

MSVVGTPKSAEQIQQEWDTNPRWKDVTRTYSAEDVVALQGSVVEEHTLARRGAEVLWEQLHDLEWVNALGALTGNMAVQQVRAGLKAIYLSGWQVAGDANLSGHTYPDQSLYPANSVPQVVRRINNALQRADQIAKIEGDTSVENWLAPIVADGEAGFGGALNVYELQKALIAAGVAGSHWEDQLASEKKCGHLGGKVLIPTQQHIRTLTSARLAADVADVPTVVIARTDAEAATLITSDVDERDQPFITGERTREGFYRTKNGIEPCIARAKAYAPFADLIWMETGTPDLEAARQFSEAVKAEYPDQMLAYNCSPSFNWKKHLDDATIAKFQKELAAMGFKFQFITLAGFHALNYSMFDLAYGYAQNQMSAYVELQEREFAAEERGYTATKHQREVGAGYFDRIATTVDPNSSTTALTGSTEEGQFH

>CORE_REP|Org155_Gene3928#

MQPMTARFDLFVVGSGFFGLTIAERVATQLDKRVLVLERRPHIGGNAYSEAEPQTGIEVHKYGAHLFHTSNKRVWDYVRQFTDFTDYRHRVFAMHNGQAYQFPMGLGLVSQFFGKYFTPEQARQLIAEQAAEIDTADAQNLEEKAISLIGRPLYEAFVKGYTAKQWQTDPKELPAANITRLPVRYTFDNRYFSDTYEGLPTDGYTAWLQNMAADHRIEVRLNTDWFDVRGQLRPGSPAAPVVYTGPLDRYFDYAEGRLGWRTLDFEVEVLPIGDFQGTAVMNYNDLDVPYTRIHEFRHFHPERDYPTDKTVIMREYSRFAEDDDEPYYPINTEADRALLATYRARAKSETASSKVLFGGRLGTYQYLDMHMAIASALNMYDNVLAPHLRDGVPLLQDGA

>CORE_REP|Org55_Gene1299#

MAEIVLDHVNKSYPDGHTAVRDLNLTIADGEFLILVGPSGCGKTTTLNMIAGLEDISSGELRIAGERVNEKAPKDRDIAMVFQSYALYPHMTVRQNIAFPLTLAKMRKADIAQKVSETAKILDLTNLLDRKPSQLSGGQRQRVAMGRAIVRHPKAFLMDEPLSNLDAKLRVQMRGEIAQLQRRLGTTTVYVTHDQTEAMTLGDRVVVMYGGIAQQIGTPEELYERPANLFVAGFIGSPAMNFFPARLTAIGLTLPFGEVTLAPEVQGVIAAHPKPENVIVGVRPEHIQDAALIDAYQRIRALTFQVKVNLVESLGADKYLYFTTESPAVHSVQLDELAEVEGESALHENQFVARVPAESKVAIGQSVELAFDTARLAVFDADSGANLTIPHRA

>CORE_REP|Org395_Gene2755#

MVPELMFDEPRPGRPPRHLADLDAAGRASAVAELGLPAFRAKQLAHQYYGRLIADPRQMTDLPAAVRDRIAGAMFPNLLTASADITCDAGQTRKTLWRAVDGTMFESVLMRYPRRNTVCISSQAGCGMACPFCATGQGGLTRNLSTAEILEQVRAGAAALRDDFGDRLSNVVFMGMGEPLANYARVLAAVQRITARPPSGFGISARAVTVSTVGLAPAIRNLADARLGVTLALSLHAPDDGLRDTLVPVNNRWRISEALDAARYYANVTGRRVSIEYALIRDVNDQPWRADLLGKRLHRVLGPLAHVNLIPLNPTPGSDWDASPKPVEREFVKRVRAKGVSCTVRDTRGREISAACGQLAAVGG

>CORE_REP|Org41_Gene2955#

MANVQYSAVTQRYPGADAPTVDNLDLDIADGEFLVLVGPSGCGKSTTLRVLAGLEPIESGRISIGDVDVTHLPPRARDVAMVFQNYALYPNMTVAANMGFALRNAGMSRADTRRRVLEVADMLELTDLLDRKPAKLSGGQRQRVAMGRAIVRRPRVFCMDEPLSNLDAKLRVSTRSQISGLQRRLGTTTVYVTHDQVEAMTMGDRVAVLKDGVLQQVDTPRALYDDPVNTFVATFIGAPAMNLIDAAVAHGVVRAPDLAIPVPDPAAERVLVGVRPESWDVASIGTPGSLTVHVELVEELGFESFVYATPVDQRGWSSRAPRIVFRTDRRTAVRVGESLAIVPHSQEVRLFNSRTETRLR

>CORE_REP|Org289_Gene1824#

MVDPTATDSPKVSIVSISYNQEEYIREALDGFAAQRTEFPVEVIIADDASTDATPRIIGEYAARYPQLFRPILRQTNIGVHANFKDVLSAARGEYLALCEGDDYWTDPLKLSKQVKYLDRHPETTVCFHPVRVIYEDGAKDSEFPPLSWRRDLSVDALLARNFIQTNSVVYRRQPSYDDIPANVMPIDWYLHVRHAVGGEIAMLPETMAVYRRHAHGIWHSAYTXXRKFWETRGHGMAATLEAMLDLVHGHREREAIVGEVSAWVLREXXXTPGXQGRALLLKSIADHXRMTMLSLQHRWAQTPWRRFKRRLSTELSSLAALAYXTRRRALEGRDGGYRETTSPPTGRGRNVRGSHA

>CORE_REP|Org180_Gene2467#

MTSRETRAADAAGARQADAQVRSSIDVPPDLVVGLLGSADENLRALERTLSADLHVRGNAVTLCGEPADVALAERVISELIAIVASGQSLTPEVVRHSVAMLVGTGNESPAEVLTLDILSRRGKTIRPKTLNQKRYVDAIDANTIVFGIGPAGTGKTYLAMAKAVHALQTKQVTRIILTRPAVEAGERLGFLPGTLSEKIDPYLRPLYDALYDMMDPELIPKLMSAGVIEVAPLAYMRGRTLNDAFIVLDEAQNTTAEQMKMFLTRLGFGSKVVVTGDVTQIDLPGGARSGLRAAVDILEDIDDIHIAELTSVDVVRHRLVSEIVDAYARYEEPGSGLNRAARRASGARGRR

>CORE_REP|Org404_Gene4037#

MKNLWNDPNMLDDGAIGRGDPSVRHHFRDSVSDTMRITDLAAPRKIPPGTGWRKFVYSVSFHKINPGESPRERHYRNLQGRIRRQYVITVVSGKGGVGVTTMAACIGGVFRECRPENVIAIDAVPSFGTLADRIDESPPGDYAAIINDTDVQGYADIREHLGQNTVGLDVLAGNRTSDQPRPLVPAMFSAVLSRLRRTHTVIVIDTSPDLEHDVMKAVLQSTDTLVFVSGITADRSRPVLRAVDYLRAQGYHELVSRSTVILNHTDSITDKDALAYLTERFTKVGAIVEAMPFDPHLAKGGIIDTVHELNKKSRLRLFEITAGLADKYVPDAERAAQ

>CORE_REP|Org327_Gene4095#

MTAPPVHDRAHHPVRDVIVIGSGPAGYTAALYAARAQLAPLVFEGTSFGGALMTTTDVENYPGFRNGITGPELMDEMREQALRFGADLRMEDVESVSLHGPLKSVVTADGQTHRARAVILAMGAAARYLQVPGEQELLGRGVSSCATCDGFFFRDQDIAVIGGGDSAMEEATFLTRFARSVTLVHRRDEFRASKIMLDRARNNDKIRFLTNHTVVAVDGDTTVTGLRVRDTNTGAETTLPVTGVFVAIGHEPRSGLVREAIDVDPDGYVLVQGRTTSTSLPGVFAAGDLVDRTYRQAVTAAGSGCAAAIDAERWLAEHAATGEADSTDALIGAQR

>CORE_REP|Org54_Gene3628#

MRLLVTGGAGFIGTNFVHSAVREHPDDAVTVLDALTYAGRRESLADVEDAIRLVQGDITDAELVSQLVAESDAVVHFAAESHVDNALDNPEPFLHTNVIGTFTILEAVRRHGVRLHHISTDEVYGDLELDDRARFTESTPYNPSSPYSATKAGADMLVRAWVRSYGVRATISNCSNNYGPYQHVEKFIPRQITNVLTGRRPKLYGAGANVRDWIHVDDHNSAVRRILDRGRIGRTYLISSEGERDNLTVLRTLLRLMDRDPDDFDHVTDRVGHDLRYAIDPSTLYDELCWAPKHTDFEEGLRTTIDWYRDNESWWRPLKDATEARYQERGQ

>CORE_REP|Org155_Gene1578#

MNAHTSVGPLDRAARVYIAGHRGLVGSALLRTFAGAGFTNLLVRSRAELDLTDRAATFDFVLESRPQVVIDAAARVGGILANDTYPADFLSENLQIQVNLLDAAVAARVPRLLFLGSSCIYPKLAPQPIPESALLTGPLEPTNDAYAIAKIAGILAVQAVRRQHGLPWISAMPTNLYGPGDNFSPSGSHLLPALIRRYDEAKASGAPNVTNWGTGTPRRELLHVDDLASACLYLLEHFDGPTHVNVGTGIDHTIGEIAEMVASAVGYSGETRWDPSKPDGTPRKLLDVSVLREAGWRPSIALRDGIEATVAWYREHAGTVRQ

>CORE_REP|Org235_Gene120#

MKVWITGAGGMMGSHLAEMLLAAGHDVYATYCRPTIDPSDLQFNGAEVDITDWCSVYDSIATFRPDAVFHLAAQSYPAVSWARPVETLTTNMVGTAIVFEALRRVRPHAKIIVAGSSAEYGFVDPSEVPINERRELRPLHPYGVSKAATDMLAYQYHKSYGMHTVVARIFNCTGPRKVGDALSDFVRRCTWLEHHPEQSAIRVGNLKTKRTIVDVRDLNRALMLMLDKGEAGADYNVGGSIAYEMGDVLKQVIAACKRDDIVPEVDPALLRPTDEKIIYGDCSKLAAITGWQQEICLTQTIADMFDYWRSKSESALMV

>CORE_REP|Org170_Gene1533#

MDRCCQRATAFACALRPTKLIDYEEMFRGAMQARAMVANPDQWADSDRDQVNTRHYLSTSMRVALDRGEFFLVYQPIIRLADNRIIGAEALLRWEHPTLGTLLPGRFIDRAENNGLMVPLTAFVLEQACRHVRSWRDHSTDPQPFVSVNVSASTICDPGFLVLVEGVLGETGLPAHALQLELAEDARLSRDEKAVTRLQELSALGVGIAIDDFGIGFSSLAYLPRLPVDVVKLGGKFIECLDGDIQARLANEQITRAMIDLGDKLGITVTAKLVETPSQAARLRAFGCKAAQGWHFAKALPVDFFRE

>CORE_REP|Org380_Gene1860#

MDQQSTRTDITVNVDGFWMLQALLDIRHVAPELRCRPYVSTDSNDWLNEHPGMAVMREQGIVVNDAVNEQVAARMKVLAAPDLEVVALLSRGKLLYGVIDDENQPPGSRDIPDNEFRVVLARRGQHWVSAVRVGNDITVDDVTVSDSASIAALVMDGLESIHHADPAAINAVNVPMEEMLEATKSWQESGFNVFSGGDLRRMGISAATVAALGQALSDPAAEVAVYARQYRDDAKGPSASVLSLKDGSGGRIALYQQARTAGSGEAWLAICPATPQLVQVGVKTVLDTLPYGEWKTHSRV

>CORE_REP|Org160_Gene816#

MRPALSDYQHVASGKVREIYRVDDEHLLLVASDRISAYDYVLDSTIPDKGRVLTAMSAFFFGLVDAPNHLAGPPDDPRIPDEVLGRALVVRRLEMLPVECVARGYLTGSGLLDYQATGKVCGIALPPGLVEASRFATPLFTPATKAALGDHDENISFDRVVEMVGALRANQLRDRTLQTYVQAADHALTRGIIIADTKFEFGIDRHGNLLLADEIFTPDSSRYWPADDYRAGVVQTSFDKQFVRSWLTGSESGWDRGSDRPPPPLPEHIVEATRARYINAYERISELKFDDWIGPGA

>CORE_REP|Org143_Gene3941#

MSDPHHPHIQTHNAWVEFPIFDAKSRSLKKAVLGKAGGTIGRNNSNVVVIEALRDITMELNLGDRVGLVGHNGAGKSTLLRLLSGIYEPTRGWAKVTGRVAPVFDLGIGMDPEISGYENIIIRGLFLGQTRKQMQAKVDEIAEFTELGEYLSMPLRTYSTGMRVRLAMGVVTSIDPEILLLDEGIGAVDADFLRKAQSRLQNLVERSGILVFASHSNEFLARLCKTAIWIDHGVIRLAGGIEEVVRAYEGEDAARHVREVLAETQADRQNVQG

>CORE_REP|Org50_Gene1540#

MTILEIKDLHVSVENPAEADHEIPILRGVDLTVKSGETHALMGPNGSGKSTLSYAIAGHPKYHVTSGTITLDGADVLAMSIDERARAGLFLAMQYPVEVPGVSMSNFLRSAATAIRGEPPKLRHWVKEVKAAMAALDIDPAFAERSVNEGFSGGEKKRHEILQLELLKPKIAILDETDSGLDVDALRVVSEGVNRYAESQHGGILLITHYTRILRYIHPEYVHVFVGGRIVESGGSELADELDQNGYVRFSPASGRYPHQPAPTGA

>CORE_REP|Org289_Gene1816#

MVSRARGNGSAMRLARRARXILRXNGIEVSRYFAELDWERNFLRQLQSHRVSAVLDVGANSGQYARGLRGAGXXXRIVSFEXXPGPFAVLQRSASTDPLWECRRCALGDVDGTISINVAGNEGASSSVLPMLKRHQDAFPPANYVGAQRVPIHRLDSVAADVLRPNDIAFLKIDVQGFEKQVIAGGDSTVHDRCVGMQLELSFQPLYEGGMLIREALDLVDSLGFTLSGLQPGFTDPRNGRMLQADGIFFRGSD

>CORE_REP|Org365_Gene857#

MELLLLTSELYPDPVLPALSLLPHTVRTAPAEASSLLEAGNADAVLVDARNDLSSGRGLCRLLSSTGRSIPVLAVVSEGGLVAVSADWGLDEILLLSTGPAEIDARLRLVVGRRGDLADQESLGKVSLGELVIDEGTYTARLRGRPLDLTYKEFELLKYLAQHAGRVFTRAQLLHEVWGYDFFGGTRTVDVHVRRLRAKLGPEHEALIGTVRNVGYKAVRPARGRPPAADPDDEDADPGRDGMQEPLVDPLRSQ

>CORE_REP|Org365_Gene512#

MTCADDDAERSDEVGAPPVALMTSVLIVEDEESLADPLAFLLRKEGFEATVVTDGPAALAEFDRAGADIVLLDLMLPGMSGTDVCKQLRARSSVPVIMVTARDSEIDKVVGLELGADDYVTKPYSARELIARIRAVLRRGGDDDSEMSDGVLESGPVRMDVERHVVSVNGDTITLPLKEFDLLEYLMRNSGRVLTRGQLIDRVWGADYVGDTKTLDVHVKRLRSKIEADPANPVHLVTVRGLGYKLEG

>CORE_REP|Org266_Gene1023#

MNRQPIVQLSNLSWTFREGETRRQVLDHITFDFEPGEFVALLGQSGSGKSTLLNLISGIEKPTTGDVTINGFAITQKTERDRTLFRRDQIGIVFQFFNLIPTLTVLENITLPQELAGVSQRKAAVVARDLLEKVGMADRERTFPDKLSGGEQQRVAISRALAHNPMLVLADEPTGNLDSDTGDKVLDVLLDLTRQAGKTLIMATHSPSMTQHADRVVNLQGGRLIPAVNRENQTDQPASTILLPTSYE

>CORE_REP|Org300_Gene3196#

MIITLDHVTKQYKSSARPALDDINVKIDKGEFVFLIGPSGSGKSTFMRLLLAAETPTSGDVRVSKFHVNKLRGRHVPKLRQVIGCVFQDFRLLQQKTVYDNVAFALEVIGKRTDAINRVVPEVLETVGLSGKANRLPDELSGGEQQRVAIARAFVNRPLVLLADEPTGNLDPETSRDIMDLLERINRTGTTVLMATHDHHIVDSMRQRVVELSLGRLVRDEQRGVYGMDR

>CORE_REP|Org184_Gene3246#

MVKVFLVDDHEVVRRGLVDLLGADPELDVVGEAGSVAEAMARVPAARPDVAVLDVRLPDGNGIELCRDLLSRMPDLRCLILTSYTSDEAMLDAILAGASGYVVKDIKGMELARAVKDVGAGRSLLDNRAAAALMAKLRGAAEKQDPLSGLTDQERTLLGLLSEGLTNKQIADRMFLAEKTVKNYVSRLLAKLGMERRTQAAVFATELKRSRPPGDGP

>CORE_REP|Org53_Gene366#

MTISFSSSNLRDDATSGNGDYRLDKLPETTPSTSVFDRADVTYRQFTELHGQARDTRREAHVVELESKTGERARCAPMHALEQLADYGFAWRDIARVVGVSVPAITKWRKGAGVTGENRLKIARLLALIDMLSDRFIGEPASWLEMPIQAGVGITRMDLLERGRYDLVLALASTHTGDGTVEYVLNETDKDWRETVVDNAFESYTAEDGVISIRPKR

>CORE_REP|Org110_Gene1928#

MKLTPVEQEKLLIFAAGELAKQRKARGVLLNYPEAAAYITCFIMEGARDGKGVAELMEAGRHVLTEKDVMEGVPEMLDSIQVEATFPDGVKLVTVHQPISAEVKS
